# Structure−Activity Relationships among Inhibitors of *Acinetobacter baumannii* and *Klebsiella pneumoniae* 1-Deoxy-d-xylulose 5-Phosphate Reductoisomerase (DXR/IspC) − A Promising Target for Antibiotic Development

**DOI:** 10.1021/acsinfecdis.5c00875

**Published:** 2026-03-12

**Authors:** Misgina Girma, Meagan Belcher Dufrisne, Archi Sehgal, Allyson Dailey, Kenneth Heidel, Darean Bague, Xu Wang, Logan Bartholomew, Richard Beck, Samuel Kirby, Haley Ball, Mosufa Zainab, Soo Hyeon Lee, Iswarduth Soojhawon, Schroeder M. Noble, Cynthia S. Dowd, Robin D. Couch

**Affiliations:** † George Mason University, Chemistry and Biochemistry, 10920 George Mason Circle, Fairfax, Virginia 22030-4444, United States; ‡ Walter Reed Army Institute of Research, Bacterial Diseases Branch, Wound Infections Department, 503 Robert Grant Ave, Silver Spring, Maryland 20910, United States; § 8367 The George Washington University, Washington, District of Columbia 20052-0086, United States; ∥ Department of Chemistry and Biochemistry, George Mason University, Manassas, Virginia 20110, United States

**Keywords:** *A. baumannii*, *K. pneumoniae*, MEP pathway, DXR/IspC, fosmidomycin analogs, Ab-IspC-inhibitor crystals

## Abstract

Antimicrobial resistance (AMR) is a critical global health crisis, responsible for nearly 5 million deaths in 2019 and projected to impose up to $100 trillion in economic costs by 2050. To address this threat, we are developing new antibiotics targeting the methylerythritol phosphate (MEP) pathway in *Acinetobacter baumannii* and *Klebsiella pneumoniae*, major nosocomial pathogens. Our efforts focus on 1-deoxy-d-xylulose 5-phosphate reductoisomerase (IspC/DXR), the committed enzyme in MEP-mediated isoprenoid biosynthesis and absent in humans, making it an attractive therapeutic target. Natural products such as fosmidomycin (FOS) and FR900098 (FR) inhibit IspC but exhibit poor bioavailability. To improve drug-like properties, we synthesized FOS analogs and analyzed their structure−activity relationships (SARs) against recombinant Ab- and Kp-IspC, alongside in vitro susceptibility assays. The most potent analogs, α,β-unsaturated compounds **4b** and **16b**, inhibited Ab-IspC (AbIspC) with IC_50_ values of 0.047 μM and 0.029 μM, and Kp-IspC (KpIspC) with 0.194 μM and 0.054 μM, respectively. These showed MICs of 64 μg/mL for *A. baumannii* and 512 μg/mL for *K. pneumoniae*. A prodrug series (**1d**−**8d**) demonstrated enhanced activity, with compound **3d** exhibiting MICs from 0.25 to 32 μg/mL against *A. baumannii*. X-ray crystallography of Ab-IspC with selected analogs (**2b**, **3b**, **4b**) at 2.0 Å resolution provides structural insights to guide future IspC inhibitor optimization.

## Graphical abstract

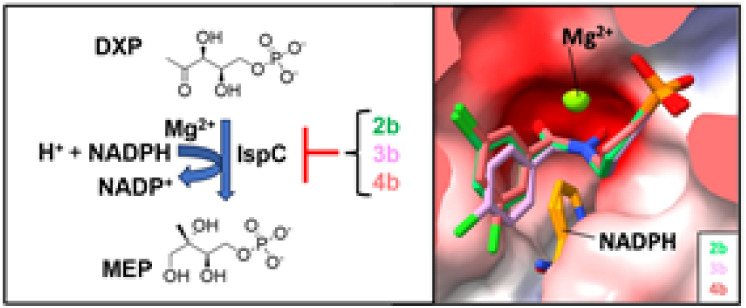

According to the World Health Organization (WHO), antimicrobial resistance (AMR) is among the greatest threats to global health.^1^ Antimicrobial-resistant pathogens cause illnesses that are difficult to treat, and consequently kill millions of people annually across the globe; nearly 5 million AMR-associated deaths were reported in 2019 alone.^2^ According to a 2016 World Bank Group report, if not addressed, AMR will become an economic catastrophe, estimated to cost the global economy a staggering $100 trillion by 2050.^3^ Clearly, effective AMR countermeasures are urgently needed.

*Acinetobacter baumannii* (*Ab*) and *Klebsiella pneumoniae* (*Kp*) are two members of the so-called ESKAPE pathogens (which also include *Enterococcus faecium*, *Staphylococcus aureus*, *Pseudomonas aeruginosa*, and *Enterobacter* species). Collectively, the ESKAPE pathogens are of particular concern because they exhibit extensive drug resistance and are responsible for a significant proportion of nosocomial infections worldwide.^4^ *Ab* is a Gram-negative coccobacillus that can survive for prolonged periods on environmental surfaces.^5^ It is an opportunistic pathogen that primarily causes healthcare-associated infections, including ventilator-associated pneumonia, bloodstream infections, and wound infections.^6^ *Kp* is a Gram-negative, encapsulated, nonmotile bacillus that is a common cause of urinary tract infections, pneumonia, and septicemia.^7^ Immunocompromised patients, those with invasive medical devices, and individuals with prolonged hospital stays are especially vulnerable to infections caused by these pathogens.^8^ Alarmingly, *Ab* and *Kp* have developed resistance to multiple classes of antibiotics, including carbapenems, which are often considered drugs of last resort.^9–11^ A 2019 global study on the burden of bacterial AMR revealed that *Ab* and *Kp* were responsible for ca. 630,000 and 810,000 deaths worldwide, respectively.^12^

To help address this issue, we have turned our efforts toward the methylerythritol phosphate (MEP) pathway, a promising drug target found in many human pathogens (Figure 1). The anabolic MEP pathway leads to isopentenyl pyrophosphate (IPP) and dimethylallyl pyrophosphate (DMAPP), 5-carbon building blocks used for isoprene biosynthesis.^9,13–18^ Isoprenoids are essential to core cellular functions such as electron transport (quinones), cell wall biosynthesis (dolichols), signal transduction (prenylated proteins), and the regulation of membrane fluidity (hopanoids and cholesterol). Accordingly, disruption of IPP and DMAPP biosynthesis is lethal to the pathogen.^16,17,19–23^ Since humans utilize the mevalonate (MVA) pathway rather than the MEP pathway for IPP and DMAPP biosynthesis, the MEP pathway is an attractive target for developing novel antibiotics.^9,13–18,24^

**Figure 1:**
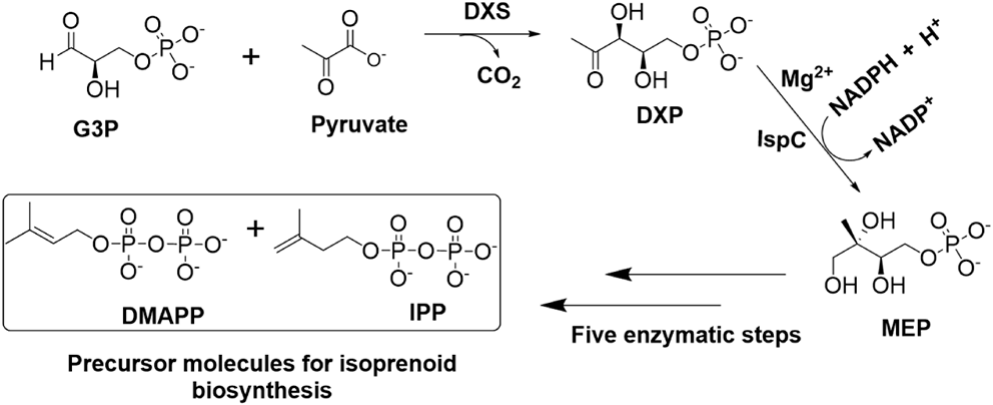
Methylerythritol phosphate (MEP) pathway of isoprenoid biosynthesis. The anabolic MEP pathway consists of seven enzymes. The first enzyme, DXS, converts G3P and pyruvate into DXP. IspC (also known as DXR) then catalyzes DXP into MEP, requiring NADPH and Mg^2+^ as cofactors. Five additional sequential enzymatic steps lead to the production of IPP and DMAPP.^13,25–27^ Abbreviations—G3P: glyceraldehyde 3-phosphate; DXS: DXP synthase; IspC/DXR: 1-deoxy-d-xylulose 5-phosphate reductoisomerase; DXP: 1-deoxy-d-xylulose 5-phosphate; MEP: 2-*C*-methyl-d-erythritol 4-phosphate; DMAPP: dimethylallyl pyrophosphate; IPP: isopentenyl pyrophosphate.

The enzyme 1-deoxy-d-xylulose 5-phosphate reductoisomerase (IspC or DXR) catalyzes the first committed step in the MEP pathway.^9,25–27^ Mechanistically, NADPH binds the enzyme first, thereby inducing a conformational change that facilitates the subsequent binding of DXP (Figure 2A).^28–31^ Upon binding, the C3 and C4 hydroxyl groups of DXP coordinate with the Mg^2+^ cofactor, and the enzyme transitions to a “closed” conformation. The C3−C4 bond undergoes heterolytic cleavage, followed by an aldol reaction, resulting in the 2-*C*-methyl-d-erythrose 4-phosphate intermediate (step 4 in Figure 2A). Final reduction via a hydride leads to the formation and release of MEP.

**Figure 2:**
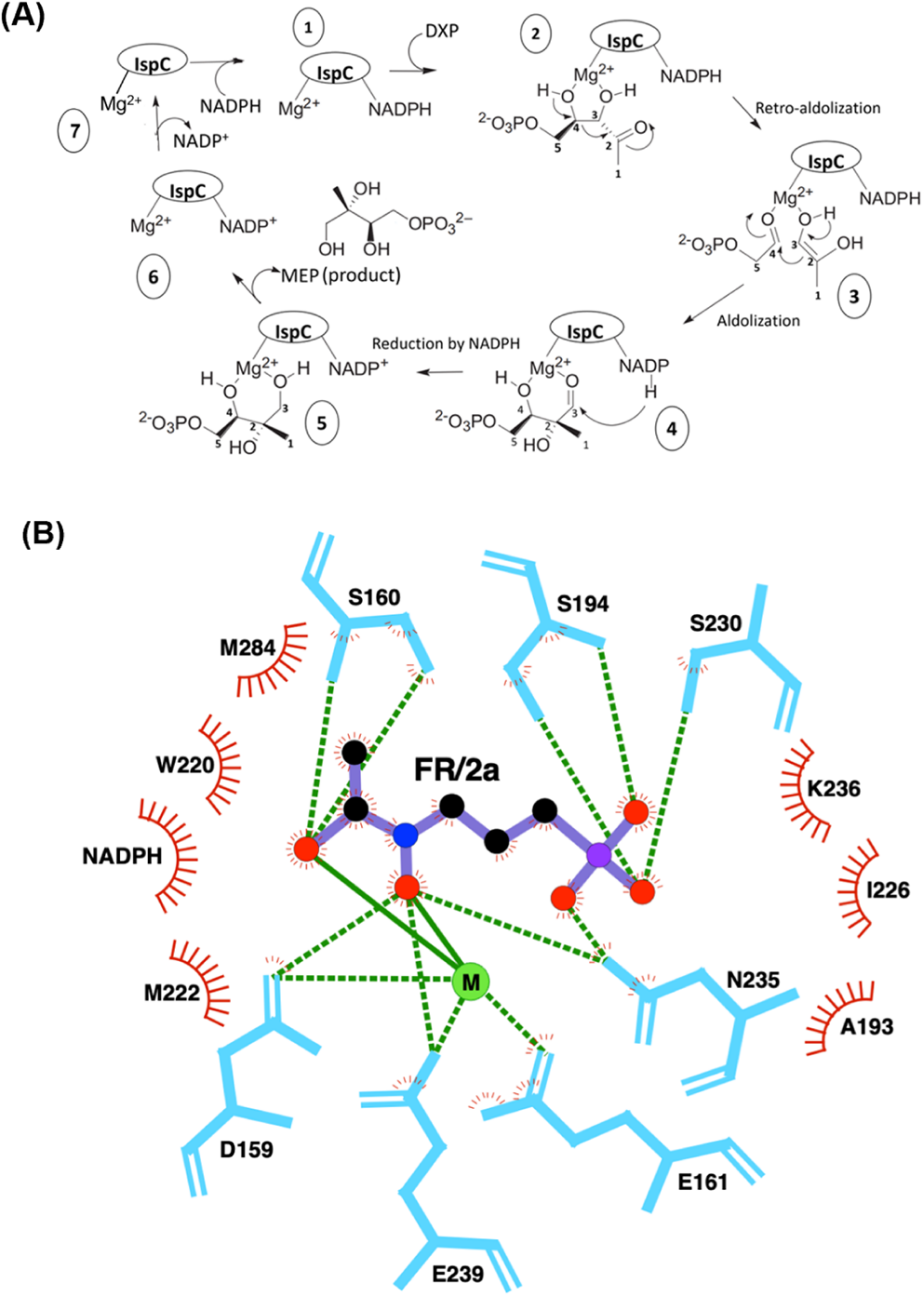
IspC catalyzed formation of MEP from DXP. (A) Key mechanistic steps include **(1)** NADPH binds to the active site of the Mg^2+^-bound enzyme; **(2)** DXP subsequently binds, coordinating Mg^2+^ via the C3 and C4 hydroxyl groups; **(3)** the C3−C4 bond is cleaved; **(4)** an aldol reaction; **(5)** reduction; and then **(6)** MEP and **(7)** NADP^+^ are released from the active site.^28–31^ Figure adapted from Li et al.^31^ (B) Fosmidomycin (FOS, **1a**) and FR900098 (FR, **2a**) are well-known competitive inhibitors of IspC, structurally mimicking the substrate DXP.^25,27,32–34^ We previously resolved a crystal structure of Ab IspC in complex with the inhibitor FR900098 (FR/**2a**; PDB ID 7S04).^9^ As shown here, the phosphonate moiety of FR/**2a** forms hydrogen bonds with S194, S230, and N235. The carbonyl oxygen of FR/**2a** forms hydrogen bonds with S160 and coordinates with the active site Mg^2+^ (M, highlighted in green). The oxygen binding to the nitrogen atom in FR/**2a** forms hydrogen bonds with D159, E239, and N235 and coordinates the active site Mg^2+^. The active site Mg^2+^ also coordinates E161 and E239, in addition to D159 and the two oxygen atoms from the inhibitor. M222, W220, and M284 shield FR/**2a** from the bulk solvent.^9,25,32–34^ FR/**2a** atoms are colored as follows: carbon = black, oxygen = red, nitrogen = blue, and phosphorus = purple.

Fosmidomycin (FOS; **1a**) and FR900098 (FR; **2a**) are small-molecule metabolites from Streptomyces *lavendulae*lavendulae and *S. rubellomurinus*, respectively, and both are phosphono-hydroxamic acids (**1a** is an *N*-formylated hydroxylamine; **2a** is its *N*-acetyl analog; see Figure 3).^35^ Despite limited bioavailability and short serum half-life in preclinical and clinical studies, **1a** and **2a** are potent in vitro inhibitors of IspC, establishing them as compelling lead scaffolds for medicinal chemistry optimization. Their validated target engagement, clear structure−activity relationship, and tractable polarity handle (phosphonate/hydroxamate) make them well-suited for prodrug and analog design aimed at improving exposure and drug-like properties while retaining on-target potency.^27,35^

**Figure 3:**
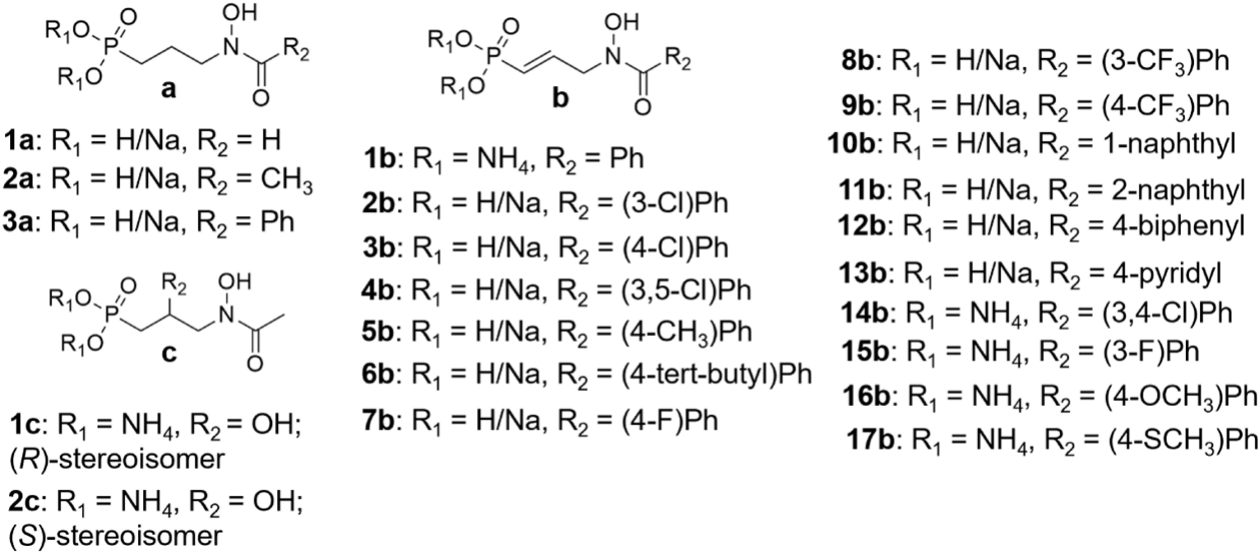
Saturated (**a** and **c**) and unsaturated *N*-acyl FOS analogs (**b**) were evaluated as inhibitors of *A. baumannii* and *K. pneumoniae* IspC. Compound **1a** = FOS and **2a** = FR900098. Compounds **1c** and **2c** are enantiomers.

We previously reported the kinetic characterization of recombinant *A. baumannii* and *K. pneumoniae* IspC and detailed the resolved crystal structure of AbIspC bound to **2a** (PDB ID: 7S04).^9^ Herein, we evaluate a suite of **1a** and **2a** analogs to define inhibitor structure−activity relationships, designing the IspC inhibitors to act as competitive inhibitors, consistent with the parent molecules (**1a**/**2a**). In addition, we performed antimicrobial susceptibility tests with these compounds to determine the minimum inhibitory concentrations (MICs) when assayed against in vitro cultures of *A. baumannii* and *K. pneumoniae*. We also determined the crystal structures of AbIspC in complex with compounds **2b**, **3b,** and **4b**.

## Results and Discussion

### Inhibitor Activity

To evaluate SARs, we screened saturated (**a** and **c**; Figure 3) and unsaturated (**b**) *N*-acyl FOS analogs against KpIspC and AbIspC using enzyme inhibition assays. At 100 μM, most compounds reduced enzyme activity by ≥90% (Figure 4). In contrast, compound **6b**, which bears a bulky (4-*tert*-butyl)­phenyl substituent on the *N*-acyl carbon, showed weak inhibition, leaving ∼47% and ∼50% residual activity for AbIspC and KpIspC, respectively, at the screening concentration. This reduced potency is consistent with a steric clash in the active site. These results indicate that smaller substituents—such as the phenyl group in **1b**—are better accommodated and yield improved inhibition (Figure 4).

Compounds **1c** and **2c** are β-substituted (relative to phosphorus) enantiomers analogs of **2a**. Screening at 100 μM highlighted the importance of the stereochemistry of the β-hydroxyl group for inhibitory activity (Figure 4): the (*R*)-enantiomer **1c** inhibited purified AbIspC and KpIspC more strongly than did the (*S*)-enantiomer **2c**.

**Figure 4:**
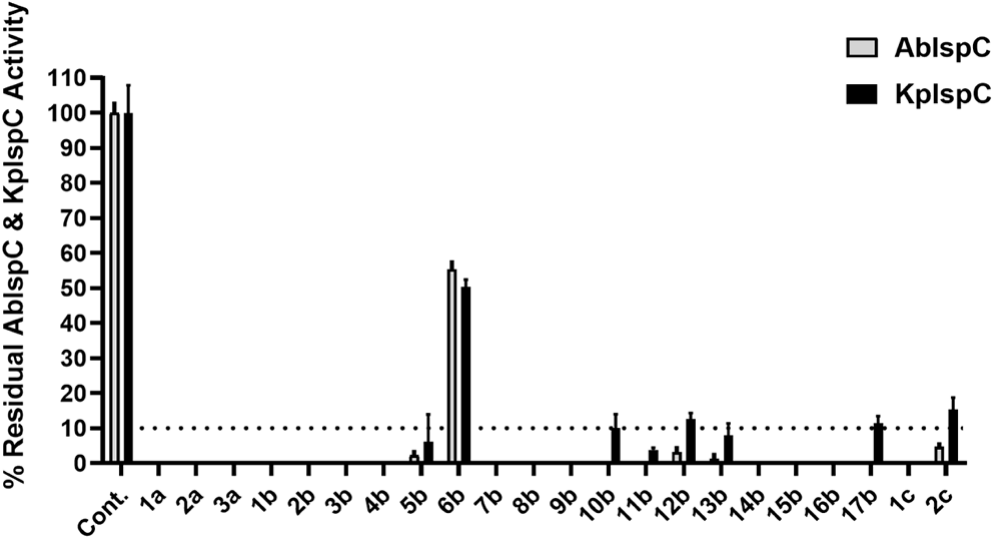
Enzyme assays to evaluate inhibitory activity. The assays were performed with each inhibitor at 100 μM, as described in Materials and Methods. Cont. = no inhibitor (vehicle control). As seen in the plot, most of the tested compounds inhibit ≥90% of the native enzyme activity. See text for further discussion.

To obtain further insight into the SARs of the most potent inhibitors (compounds achieving ≥90% inhibition in the initial screen), we determined IC_50_ values in follow-on assays (Table 1). Fosmidomycin (**1a**) and its *N*-acetyl analog FR900098 (**2a**) served as reference inhibitors. Against AbIspC and KpIspC, **1a** resulted in IC_50_ values of 0.047 μM and 0.020 μM, respectively, while **2a** gave rise to IC_50_ values of 0.024 μM and 0.023 μM (Table 1).^9^ As a side note, the methyl group in **2a** enhances inhibition of *Plasmodium falciparum* IspC relative to **1a** by forming favorable hydrophobic contacts in the active site, as observed in crystallographic analyses (e.g., PfIspC, PDB 3AUA) where the methyl is seen to engage Trp296 and Met298 similar to the C1 position of the natural substrate DXP.^33,36^ Consistent with these interactions, the equivalent Trp and Met residues in AbIspC are also seen to associate with the methyl group of **2a** (PDB 7S04), aligning with the observed 2-fold greater potency of **2a** versus **1a** against AbIspC (Table 1).^9^

**Table 1:**
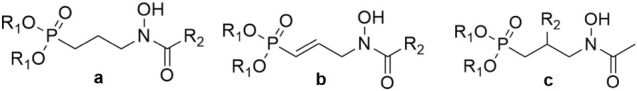
Inhibitory Activity of the FOS Analogs Shown in Figure 3. Half-Maximum Inhibitory Concentrations (IC_50_) Were Derived from In Vitro Enzyme Assays Performed with Purified Recombinant AbIspC or KpIspC^c^ ^d^

					IC_50_, μM	
Compound	R_1_	R_2_	Bacteria strain	MIC (μg/mL)	AbIspC	KpIspC
**1a** (FOS)	Na/H	H	AB5075	>512	0.047^a^	0.020^a^
			AB5711	>512		
			KP4640	>512		
**2a** (FR)	Na/H	CH_3_	Ab5075	256	0.024^a^	0.023^a^
			Ab5711	>256		
			KP4640	>256		
**3a**	Na/H	Ph	AB5075	>256	0.232	0.826
			AB5711	>256		
**1b**	NH_4_	Ph	AB5075	>512	0.459	2.65
			AB5711	>512		
**2b**	Na/H	(3-Cl)Ph	AB5075	128	0.154	0.692
			AB5711	128		
			KP4640	>512		
**3b**	Na/H	(4-Cl)Ph	AB5075	>256	0.172	0.924
			AB5711	>256		
			KP4640	>512		
**4b**	Na/H	(3,5-Cl)Ph	AB5075	64	0.047	0.194
			AB5711	64		
			KP4640	512		
**14b**	NH_4_	(3,4-Cl)Ph	AB5075	>256	2.147	3.721
			AB5711	>256		
			KP4640	>512		
**15b**	NH_4_	(3-F)Ph	AB5075	>256	0.363	0.450
			AB5711	256		
			KP4640	>512		
**5b**	Na/H	(4-CH_3_)Ph	AB5075	ND^b^	1.777	4.670
			AB5711	ND		
			KP4640	ND		
**6b**	Na/H	(4-*tert*-butyl)Ph	AB5075	ND	(53.18)^φ^	(50.39)
			AB5711	ND		
			KP4640	ND		
**7b**	Na/H	(4-F)Ph	AB5075	>128	0.245	0.323
			AB5711	>128		
			KP4640	>512		
**8b**	Na/H	(3-CF_3_)Ph	AB5075	>128	0.167	0.852
			AB5711	>128		
			KP4640	>512		
**9b**	Na/H	(4-CF_3_)Ph	AB5075	ND	4.776	8.633
			AB5711	ND		
			KP4640	ND		
**10b**	Na/H	1-naphthyl	AB5075	ND	1.432	14.74
			AB5711	ND		
			KP4640	ND		
**11b**	Na/H	2-naphthyl	AB5075	ND	3.048	31.72
			AB5711	ND		
			KP4640	ND		
**12b**	Na/H	4-biphenyl	AB5075	ND	7.353	18.090
			AB5711	ND		
			KP4640	ND		
**13b**	Na/H	4-pyridyl	AB5075	>128	6.001	27.660
			AB5711	>128		
			KP4640	>512		
**16b**	NH_4_	(4-OCH_3_)Ph	AB5075	64	0.029	0.057
			AB5711	64		
			KP4640	512		
**17b**	NH_4_	(4-SCH_3_)Ph	AB5075	>128	9.196	18.020
			AB5711	>128		
			KP4640	>512		
**1c**	NH_4_	OH (*R*)-enantiomer	AB5075	>128	0.086	0.096
			AB5711	>128		
**2c**	NH_4_	OH (*S*)- enantiomer	AB5075	128	2.332	1.986
			AB5711	128		

a We previously reported these IC_50_ values.^9^

b ND = not determined.

c Values in parentheses are the % residual enzyme activity at 100 μM concentration of the inhibitor.

d Minimum inhibitory concentrations (MICs) were derived from growth inhibition assays performed with *A. baumannii* AB5075, *A. baumannii* AB5711, or *K. Pneumoniae* KP4640. Assays were performed in triplicate. Assay details are provided in Materials and Methods.

Saturated analog **3a** (*N*(OH)-benzoyl) outperformed its unsaturated counterpart **1b**, showing inhibition of AbIspC and KpIspC with IC_50_ values of 0.232 μM and 0.826 μM, respectively, whereas **1b** required 2−3× higher concentrations to achieve comparable effects (Table 1). While this suggests that inhibitor potency is enhanced by conformational flexibility afforded by a single bond, it is notable that unsaturated compounds **4b** and **16b** each have potencies roughly an order of magnitude more potent than that obtained with saturated analog **3a**.

SAR trends indicate that adding chloro- or methoxy-substituents to the phenyl group of the retrohydroxamate generally increases inhibitory potency against AbIspC and KpIspC. Within the α,β-unsaturated series (**1b**−**17b**), **16b** is the most potent, with IC_50_ values of 0.027 μM (AbIspC) and 0.057 μM (KpIspC), representing ∼16-fold and ∼46-fold improvements over **1b** (0.459 μM and 2.65 μM, respectively; Table 1). Whereas **1b** bears a phenyl substituent, **16b** replaces it with a 4-methoxyphenyl group. The methoxy substituent likely enhances binding via favorable electronic effects and by offering an oxygen that can participate in hydrogen-bonding interactions and a methyl that can strengthen hydrophobic contacts in the active site, collectively improving inhibition relative to **1b**.^35^

Compound **4b**, which carries a 3,5-dichlorophenyl substituent, showed strong inhibition of AbIspC and KpIspC with IC_50_ values of 0.047 μM and 0.194 μM, respectively. SAR trends indicate that chlorination on the phenyl ring markedly boosts potency, with the 3,5-dichloro derivative (**4b**) outperforming both the monochlorinated (**2b**) and unsubstituted (**1b**) analogs (Table 1), a feature that is further elaborated upon in the crystallography section below. Together, these results highlight strategic chlorination as a useful handle for designing next-generation IspC inhibitors with enhanced antimicrobial potential.

Replacing the phenyl group of compound **1b** with bulky hydrophobic groups in **5b**, **6b**, **10b**, **11b**, and **12b** significantly increased resulting IC_50_ values (Table 1), presumably due to steric hindrance and suboptimal binding at the enzyme active site. While these modifications enhance cell membrane penetration (as evidenced by San Jose et al.^37^), they compromise inhibitory potency. Hence, improving cellular permeability by modifying inhibitor structure may be challenging, whereas a prodrug approach may be much more favorable.^35^ We and others have previously described an evaluation of β-substituted FOS analogs.^18,38–41^

Compounds **1c** and **2c** are β-substituted **2a** analogs, containing a hydroxyl substitution at the β position (relative to the phosphorus atom). The *R*-enantiomer compound **1c** demonstrates superior potency against AbIspC and KpIspC, with IC_50_ values of 0.083 μM and 0.096 μM, respectively, representing a 28-fold and 2-fold improvement over **2c** (Table 1). This difference in inhibitory activity underscores the importance of stereochemistry at this position, which could reflect hydrogen bonding interactions with active site residues, contributing to enhanced affinity and enzyme inhibition. Looking at Figure 2B, which depicts **2a** bound to the AbIspC active site, suggests that the β-hydroxyl group of **1c** could interact with active site residues E161, E239, N235, S194, and the Mg^2+^ ion. Efforts are underway to obtain a crystal structure with **1c** bound.

A comparative analysis of compounds **1a**, **3a**, **1b**−**5b**, **7b**−**17b**, **1c**, and **2c** reveals preferential binding to AbIspC over KpIspC (Table 1). This preferential binding cannot be attributed to differences in key active-site residues, as these amino acids are strictly conserved between the two homologues (see the multiple sequence alignment in the Supporting Information). This alludes to the occurrence of more subtle structural differences between AbIspC and KpIspC contributing toward inhibitor binding specificity. Efforts are underway to obtain a KpIspC crystal structure.

### Antimicrobial Susceptibility Test

Antimicrobial susceptibility testing (AST) was used to determine MICs of selected IspC inhibitors against *A. baumannii* and *K. pneumoniae* cultures (Table 1). Overall, all tested compounds were found to be more effective against the *A. baumannii* cultures than they were against the *K. pneumoniae* cultures. In *A. baumannii*, **4b** showed markedly improved activity with an MIC of 64 μg/mL, which is a four- to 8-fold enhancement over the parent compounds **1a** and **2a** (MIC > 512 μg/mL and >256 μg/mL, respectively). Notably, although **4b** and **1a** have identical activity against purified recombinant AbIspC (IC_50_ = 0.047 μM), **4b** delivers superior whole-cell efficacy. The same can be said about **16b**, which parallels **4b** in inhibitory activity against AB5075 and AB5711, with an MIC of 64 μg/mL toward each.

Among the chlorinated analogs, it is notable that **4b** (3,5-dichlorophenyl) demonstrates enhanced potency over **14b** (3,4-dichlorophenyl), **2b** (3-chlorophenyl), and **3b** (4-chlorophenyl), presumably because the two meta chlorines deliver strong, cumulative inductive withdrawal, higher lipophilicity and polarizability, and a symmetric aryl face that can maximize hydrophobic, halogen-bond, and shape complementarity interactions with the enzyme, and/or improve permeability/retention of the inhibitor. Additionally, at the meta position, the less electronegative chlorine shows slightly better potency than a fluorine (**2b** vs **15b**), while a fluorine at the para position has better MIC activity relative to chloro (**7b** vs **3b**; although **3b** is a more effective inhibitor of the purified enzyme). In contrast, **1b** (containing an unsubstituted phenyl) failed to inhibit *A. baumannii* growth at 512 μg/mL.

Overall, **4b** and **16b** are outstanding among the compound set, demonstrating the greatest potency in both the enzyme and growth inhibition assays. The results highlight opportunities for further optimization to achieve even greater potency against *A. baumannii*. The mechanistic basis for *A. baumannii*’s higher sensitivity relative to *K. pneumoniae* remains unresolved, however, and additional studies are needed to define the uptake, permeability, and target-access factors underlying this disparity.

### Evaluation of Prodrug Analogs of **1a** and **2a**

We also evaluated pivaloyloxymethyl (POM) and benzoyloxymethyl (BOM) prodrug analogs of **1a** and **2a** for antibacterial activity against *A. baumannii* (Tables 2–3) and *K. pneumoniae* (Table 2) by determining MICs. These prodrugs were designed to enhance cellular penetration; once inside the bacterium, endogenous esterases cleave the POM, BOM, or BOM-derived groups to release the active species and inhibit IspC. Against *A. baumannii*, the prodrugs yielded promising MICs of 0.25−16 μg/mL (Tables 2–3). The most potent series, **2d**−**5d**, showed MICs of 0.25−32 μg/mL across *A. baumannii* strains and share a common design: a BOM, 4-Me-BOM, or 4-OMe-BOM group at R_1_ and a methyl substituent at R_2_. Increasing bulk at R_2_ diminished potency—substituting methyl with phenyl (**6d**) or 3-chlorophenyl (**7d**) raised MICs to 32−128 μg/mL (Tables 2–3). Likewise, replacing the BOM group of **2d** with POM (**8d**) increased the AB5075 MIC from 4 μg/mL to 64 μg/mL, indicating BOM is the preferred moiety for anti-*A. baumannii* activity. Overall, SARs indicate that a methyl at R_2_ (**2d**, **4d**, **5d**) and para electron-donating groups (CH_3_ or OCH_3_) on the BOM ring at R_1_ (**4d**, **5d**) are key drivers of potency (Table 2). In contrast, all prodrugs were relatively weak against *K. pneumoniae* (MICs > 64 μg/mL; Table 2). These results support **1a**/**2a** BOM-type prodrugs as promising leads for *A. baumannii* infections and point to the need for further structural refinement to achieve activity against *K. pneumoniae*.

**Table 2:**
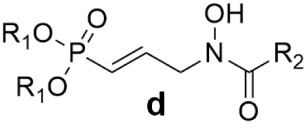
Minimum Inhibitory Concentrations (MICs) of Pivaloyloxymethyl (POM) and Benzoyloxymethyl (BOM) Prodrugs Analogs against *A. baumannii* and *K. pneumoniae* Strains

Compound	R_1_	R_2_	Assayed bacteria	MIC (μg/mL)
**1d**	BOM	H	AB5075	16
			AB5711	32
			AB19606 (ATCC)	32
			KP4640	>64
			KP43816 (ATCC)	>64
**2d**	BOM	Me	AB5075	4
			AB5711	8
			AB19606	8
			KP4640	>64
			KP43816	>64
**3d**	4-Cl-BOM	Me	AB5075	8
			AB5711	16
			AB19606	16
			KP4640	>64
**4d**	4-Me-BOM	Me	AB5075	4
			AB5711	4
			KP4640	>64
**5d**	4-OMe-BOM	Me	AB5075	4
			AB5711	4
			KP4640	>64
**6d**	BOM	Ph	AB5075	128
			KP4640	>128
**7d**	4-Cl-BOM	3-Cl-Ph	AB5075	32
			AB5711	32
			AB19606	64
			KP4640	>64
			KP43816	>64
**8d**	POM	Me	AB5075	64
			KP4640	>128
**Rifampin**	-	-	AB5075	4
			AB5711	4
			AB19606	2
			KP4640	32
			KP43816	64

**Table 3:**
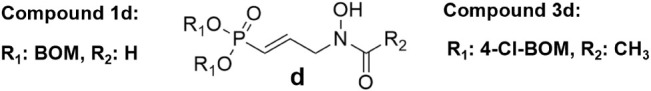
Minimum Inhibitory Concentrations (MICs) of the Benzoyloxymethyl (BOM) Prodrugs **1d** and **3d** Assayed against an *Ab* Panel

*A. baumannii* strain assayed	Compound 1d MIC (μg/mL)	Compound 3d MIC (μg/mL)	Rifampin MIC (μg/mL)
AB5075	16^a^	8^a^	4
AB5711	32^a^	16^a^	4
AB3560	32	16	8
AB3638	16	8	2
AB0087	32	8	2
AB4052	64	32	8
AB5674	32	16	4
AB0967	32	16	8
AB4795	32	32	4
AB4957	64	32	8
AB585	4	0.25	2
AB499	0.5	0.25	2

a MIC values reported in Table 2 above are included here for comparison.

### Crystal Structure of AbIspC in Complex with Mg^2**+**^, NADPH, and Inhibitors **2b**, **3b,** or **4b**

Crystals of AbIspC were grown and soaked with Mg^2+^, NADPH, and three different compounds: **2b**, **3b,** or **4b** according to methods we described previously (PDB ID: 7S04).^9^ Data were collected to 2.0 Å resolution for structure determination (resolved structures are presented in Figure 5; data collection and refinement statistics are found in Table 4). The overall architecture of the protein closely resembles previously published apo (PDB ID: 4ZN6) and FR/**2a**-bound (PDB ID: 7S04) structures of AbIspC. This structure contains a monomer in the asymmetric unit, and the biological assembly of a dimer is observed in crystallographic symmetry, as previously observed (PDB ID: 7S04).^9^ The overall fold of the monomer is identical to previous AbIspC structures with the same three-domain architecture previously described: an N-terminal NADPH-binding domain, a central catalytic domain, and a C-terminal helical domain (Figure 5). The central catalytic domain is where all three compounds **2b**−**4b** bind (Figure 5B and Figure 6).

**Figure 5:**
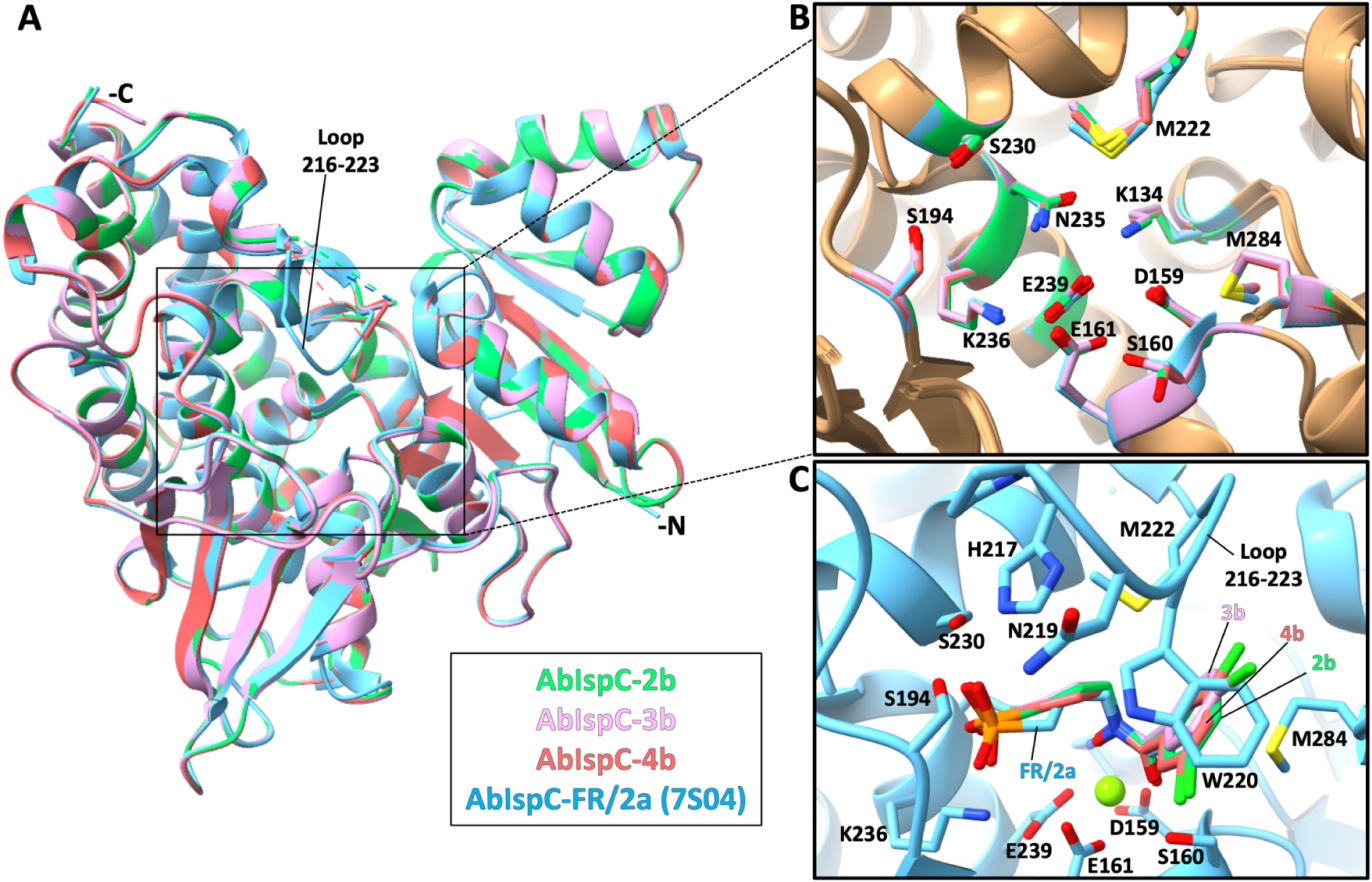
Alignment of AbIspC structures in the “closed” conformation. Resulting compound-bound structures of AbIspC were aligned and presented here. (A) Overall architecture of three previously unreported structures (**2b** in light green; **3b** in pink; **4b** in salmon) are shown in ribbon representation along with published AbIspC bound to FR900098 (FR; in light blue). (B) The residues responsible for substrate and small-molecule binding are shown in the same color scheme as (A) with the rest of the aligned structures colored tan. Residues H217 and W220 were excluded as they are unresolved in three of the structures depicted. Some loop regions were removed for clarity. (C) Aligned structures showing **2b**, **3b**, **4b,** and FR/**2a** in the catalytic domain of AbIspC (PDB ID: 7S04) showing W220 from Loop 216−223 clashing with **2b**, **3b**, and **4b**. The PDB IDs are AbIspC-**2b**: 9OZF, AbIspC-**3b**: 9OZE, AbIspC-**4b**: 9OZG.

**Figure 6:**
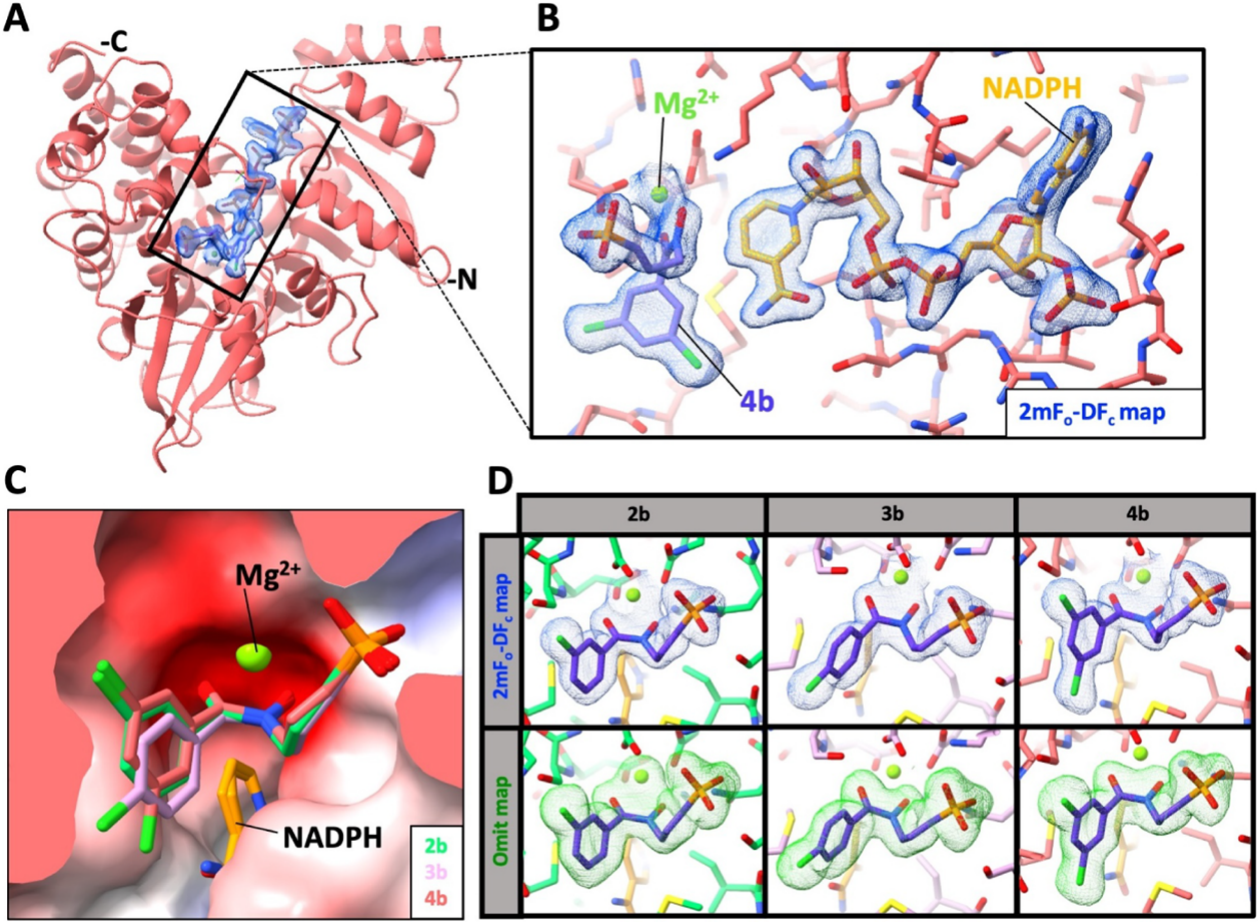
Crystallographic evidence for small molecule and substrate binding to AbIspC. (A) The overall architecture of AbIspC is shown in ribbon representation and colored salmon. Bound small molecule **4b** (colored periwinkle) and NADPH (colored gold) are shown in their respective binding sites. A more detailed representation of these molecules is shown in (B) with AbIspC in stick representation with the same coloring, and Mg^2+^ shown as a lime green sphere. 2mF_o_−DF_c_ electron density maps are all shown at σ = 1.0 in blue mesh. (C) **2b**, **3b**, and **4b** bound to aligned structures are shown in the binding pocket of AbIspC, shown in surface representation, colored by electrostatic potential (negative red, positive blue). Small molecules are colored light green (**2b**), pink (**3b**), and salmon (**4b**), respectively. (D) The 2mF_o_−DF_c_ electron density maps are shown within 1.6 Å of the bound substrate and Mg^2+^ at σ = 1.0 in blue mesh. Omit maps of bound compounds are shown within 2.5 Å of the compound at σ = 3.0 in green mesh. The structures’ PDB IDs are AbIspC-**2b**: 9OZF, AbIspC-**3b**: 9OZE, and AbIspC-**4b**: 9OZG.

**Table 4:** Crystallography Data Collection and Refinement Statistics for AbIspC Bound to NADPH, Mg^2+^, and Compounds **2b**, **3b**, and **4b**

	AbIspC:2b complex (A2)	AbIspC:3b complex (A3)	AbIspC:4b complex (A1)
Wavelength	1.03320	1.03320	1.03320
Resolution range	39.76−2.0 (2.071−2.0)	39.68−2.0 (2.072−2.0)	39.95−2.0 (2.072−2.0)
Space group	P 2 21 21	P 2 21 21	P 2 21 21
Unit cell	53.899 66.871 117.773 90 90 90	54.155 67.05 118.038 90 90 90	54.141 67.073 118.366 90 90 90
Total reflections	58980 (5756)	59558 (5808)	59730 (5846)
Unique reflections	29492 (2877)	29779 (2895)	29867 (2922)
Multiplicity	2.0 (2.0)	2.0 (2.0)	2.0 (2.0)
Completeness (%)	99.95 (99.97)	99.88 (99.69)	99.73 (99.97)
Mean I/sigma(I)	17.47 (4.15)	21.08 (3.43)	16.56 (4.00)
Wilson B-factor	24.29	27.21	24.40
R-merge	0.02693 (0.1659)	0.02161 (0.2113)	0.03028 (0.1876)
R-meas	0.03809 (0.2346)	0.03056 (0.2988)	0.04283 (0.2653)
R-pim	0.02693 (0.1659)	0.02161 (0.2113)	0.03028 (0.1876)
CC1/2	0.999 (0.929)	1 (0.933)	0.999 (0.852)
CC^a^	1 (0.981)	1 (0.983)	1 (0.959)
Reflections used in refinement	29485 (2877)	29752 (2896)	29792 (2922)
Reflections used for R-free	2902 (283)	2996 (300)	3003 (302)
R-work	0.1873 (0.2203)	0.1873 (0.2567)	0.1895 (0.2603)
R-free	0.2001 (0.2428)	0.2030 (0.2846)	0.2088 (0.2874)
CC (work)	0.960 (0.898)	0.963 (0.883)	0.964 (0.819)
CC (free)	0.957 (0.884)	0.949 (0.840)	0.961 (0.755)
Number of non-hydrogen atoms	3240	3242	3257
Macromolecules	2918	2947	2944
Ligands	94	95	84
Solvent	228	200	229
Protein residues	392	393	394
RMS (bonds)	0.012	0.014	0.008
RMS (angles)	1.41	1.67	1.03
Ramachandran favored (%)	99.48	98.71	98.21
Ramachandran allowed (%)	0.52	1.29	1.79
Ramachandran outliers (%)	0.00	0.00	0.00
Rotamer outliers (%)	1.59	1.59	1.59
Clashscore	2.49	4.29	3.31
Average B-factor	27.00	30.24	27.50
Macromolecules	26.64	30.00	27.21
Ligands	25.30	29.03	25.49
Solvent	32.43	34.40	31.96

a Statistics for the highest resolution shell are shown in parentheses.

Structures of AbIspC bound to compounds **2b** (PDB ID: 9OZF), **3b** (PDB ID: 9OZE), or **4b** (PDB ID: 9OZG) more closely resemble the “closed structure” described previously when bound to FR/**2a**.^9^ However, for structures bound to compounds **2b**, **3b**, or **4b**, much of the flexible loop region (residues 216−223) was not resolved (Figure 5A) and does not close over the active site as observed in the FR/**2a**-bound AbIspC structure (PDB ID: 7S04).^9^ In the overlaid structures, the chloro/dichloro-substituted phenyl rings of **2b**, **3b,** and **4b** clash with the ordered loop seen in the FR/**2a**-bound structure (Figure 5C). Simple steric hindrance of the bound compound may explain the flexibility observed in this loop in all three structures, preventing it from being sufficiently ordered to be resolved. There is no major difference between the protein conformation of the four “closed” structures (AbIspC bound to **2b**, **3b**, **4b** and FR/**2a**) either in the overall protein architecture (Figure 5A, Table 5) or in the residues involved in ligand binding (Figure 5B, Table 6) with the RMSD values between all closed structures being below 0.32 Å. In contrast, all compound-bound structures compared to “open” conformation of the apo structure (PDB ID: 4ZN6) had RMSD values between 2.1 and 2.3 Å when comparing the whole protein backbone (Table 5), and between 1.3 and 1.5 Å for residues involved in ligand binding (Table 6).

**Table 5:** RMSD Values (Å) Comparing CA−CA Backbone of Overlayed AbIspC Structures

	AbIspC:2b complex	AbIspC:3b complex	AbIspC:4b complex	AbIspC:FR complex (PDB ID 7S04)
AbIspC:3b complex	0.154			
AbIspC:4b complex	0.133	0.159		
FR complex (PDB ID 7S04)	0.218	0.271	0.220	
Apo (PDB ID 4ZN6)	2.223	2.153	2.177	2.209

**Table 6:** RMSD Values (Å) Comparing Invariant, Ligand Binding Residues of Overlayed AbIspC Structures − K134, D159, S160, E161, S194, M222, S230, N235, K236, E239, M284; Excluded Loop Residues H217, W220 as They Are Not Resolved in Some Structures

	AbIspC:2b complex	AbIspC:3b complex	AbIspC:4b complex	AbIspC:FR complex (PDB ID 7S04)
AbIspC:3b complex	0.287			
AbIspC:4b complex	0.117	0.293		
FR complex (PDB ID 7S04)	0.319	0.244	0.309	
Apo (PDB ID 4ZN6)	1.447	1.377	1.414	1.380

The coordination of three compounds presented here is very similar to the previously observed binding of small molecules to the central catalytic domain of IspC (Figure 5C). Two water molecules and residues S194 and N235 (shown in Figure 2B) are involved in stabilizing the phosphonate group through a hydrogen bonding network. H217 (equivalent to PfIspC H293) from flexible loop 216−223 is not observed, but additional residues S230 and K236 are involved in phosphonate binding (Figure 7). The retro hydroxamate moiety interacts with the divalent cation (modeled as Mg^2+^) coordinated by D159, E161, and E239 (PfIspC D231, E233 and E315) and forms additional hydrogen bonds with residues S160 (PfIspC S232) and K134. Compound **4b** shows extra hydrogen bonding with a water molecule stabilized by NADPH (Figure 7). Compared to **2b** and **3b**, this may explain the improved IC_50_ value of compound **4b** against both AbIspC and KpIspC enzymes and the better MIC values (Table 1) in the absence of stabilizing interactions from the flexible loop (residues 216−223) or the flexible loop shielding the competitive inhibitor from the solvent as previously observed (PDB ID 7S04).^9^

**Figure 7:**
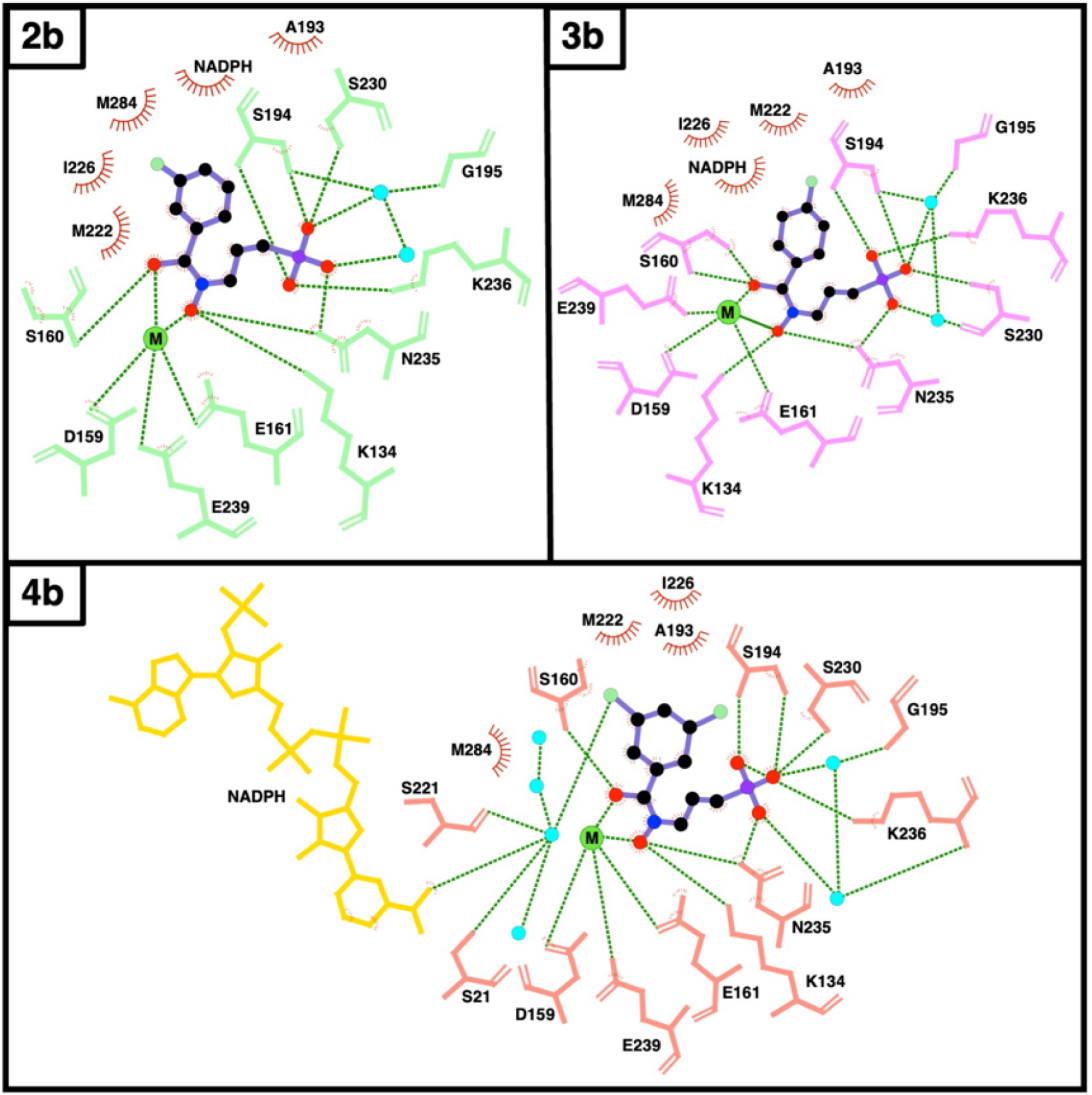
Binding mode of small molecules to AbIspC. Two-dimensional representations of small molecules bound to the catalytic domain of AbIspC are shown. Small molecules in stick representation are colored purple. NADPH is shown in stick representation, colored gold. Mg^2+^ is shown as a lime green circle, labeled “M.” Water molecules are represented as cyan circles. Hydrogen bonding amino acid residues in stick representation are colored light green (AbIspC bound to **2b**), pink (AbIspC bound to **3b**) and salmon (AbIspC bound to **4b**). Hydrogen bonds are represented as dark green dashed lines. Hydrophobic residues are shown in red semicircles.

## Conclusion

AMR represents a global health crisis, claiming millions of lives annually.^1,2^ Multidrug-resistant *A. baumannii* and *K. pneumoniae* species are common causes of nosocomial infections in hospital settings and contribute to antimicrobial resistance.^10,42,43^ Thus, novel antibiotics are needed to counter the evolving problem. The MEP pathway is an attractive drug target because it is essential in some bacteria and certain protozoa but is absent in humans.^25,27^ Significant progress has been made in developing novel fosmidomycin (**1a**) analogs in the pursuit of developing novel “MEPicides”.^44–47^ In this study, we demonstrated that several **1a** analogs, compounds **2b**, **3b**, **4b**, **8b**, **16b**, and **1c,** are potent inhibitors of IspC, particularly AbIspC. Compounds **4b**, **16b**, **2b**, and **2c** showed MICs ranging from 64 μg/mL to 128 μg/mL against *A. baumannii* strains AB5075 and AB5711. This represents a significant improvement in antibacterial activity compared to the parent compound **1a**, which showed MIC > 512 μg/mL against the same strains. We showed that the prodrug compounds **1d**−**8d** have even better anti-*A. baumannii* activity (MICs: 4−64 μg/mL, Table 2), and compound **3d** showed MICs ranging from 0.25 to 32 μg/mL against an *A. baumannii* clinical isolate panel (Table 3). We also report crystal structures of AbIspC in complex with compounds **2b**, **3b,** and **4b** at 2.0 Å resolution. Structural information provides chemical evidence for a higher degree of hydrogen bonding of compound **4b** to AbIspC independent of residues on the flexible loop (216−223), which correlates with more potent inhibition as demonstrated by lower MIC and IC_50_ values of this compound. Our findings establish the basis for optimizing additional analogs to enhance their potency against the target bacteria. This work advances MEP pathway targeting inhibitors (“MEPicides”) as potential therapeutics for *A. baumannii* and *K. pneumoniae* infections. Future research warrants optimizing the analogs to improve antibacterial efficacy and pharmacological profiles.

## Materials and Methods

### Reagents and Chemical Synthesis

#### Synthesis of the *N*-Acyl Phosphonic Acid Salts and Prodrugs

A combination of TLC and column chromatography was used to purify the compounds. Purity was evaluated using a combination of ^1^H and ^13^C NMR, along with mass spectrometry (HPLC-MS, GC-MS, HRMS). All assayed compounds were 95% pure or greater. Compounds **1c** and **2c** were stereospecifically synthesized from chiral starting materials. No chiral separation was required. Optical purity was measured using a polarimeter. A comprehensive account of the synthesis of the compounds examined in this study will be presented in a subsequent publication, while the characterization of the compounds is presented in the Supporting Information.

#### Purification of the IspC Enzymes

Our previous work reported the cloning, expression, and purification of *Ab* and *Kp* IspC enzymes.^9^ Briefly, *E. coli* BL21 CodonPlus (DE3)-RIL containing each of the AbIspC and KpIspC constructs was used as an expression strain. To express each of the *A. baumannii* and *K. pneumoniae* IspC, 10 mL overnight (approximately 18 h) seed culture of *E. coli* BL21 CodonPlus (DE3)-RIL was used to inoculate 1 L of LB media; the media contained 100 μg/mL ampicillin and 50 μg/mL chloramphenicol; cells were incubated at 37 °C with shaking at 250 rpm. Cells were grown until the OD_600_ reached 1−1.2. To induce protein production, 0.5 mM (final concentration) isopropyl β-d-thiogalactopyranoside (IPTG) was added to the culture, and then cells were incubated for 18 h at 37 °C with shaking at 250 rpm. Then, cells were harvested by centrifugation at 9300*g* for 20 min at 4 °C; the resulting cell pellets were stored at −80 °C until required to perform protein purification.

Affinity chromatography was used for purification. Bacterial cells stored at −80 °C were thawed on ice for 20 min, lysed with lysis buffer A (100 mM Tris pH 8.0, 0.032% w/v lysozyme, 3 mL per gram cell pellet), and then lysis buffer B was added (0.1 M CaCl_2_, 0.1 M MgCl_2_, 0.020% DNase, 0.3 mL per gram cell pellet). After incubating with lysis buffer for 10 min, NaCl solution was added to a final concentration of 0.1 M. To separate the supernatant containing the soluble protein from the rest of the cell debris, the cell lysate was centrifuged at 48,000*g* for 30 min at 4 °C. The supernatant was passed through a TALON immobilized metal affinity column (Clontech Laboratories, Mountain View, CA). Then, the column was washed with three different buffers one after the other: 20 column volumes of equilibrium buffer (50 mM HEPES pH 7.5, 300 mM NaCl), 10 column volumes of wash buffer A (50 mM HEPES pH 7.5, 300 mM NaCl, 10 mM imidazole), and 15 column volumes of wash buffer B (100 mM HEPES pH 7.5, 600 mM NaCl, 20 mM imidazole). To elute the protein from the column, 5 column volumes of elution buffer were used (150 mM imidazole pH 7.0, 300 mM NaCl). The protein was concentrated by centrifugation at 4000*g* and buffer exchanged by adding protein storage buffer (100 mM Tris pH 7.5, 1 mM NaCl, 5 mM DTT) to the protein concentrator (10,000 kDa molecular weight cutoff, MilliporeSigma). Advanced Protein Assay Reagent (Cytoskeleton, Denver, CO) was used for determining the protein concentration by measuring the absorbance at 600 nm; solutions of γ-globulin (Sigma-Aldrich) were used as standard. To assess the purity of the purified protein, we performed sodium dodecyl sulfate−polyacrylamide gel electrophoresis (SDS-PAGE) and then stained the gel with Coomassie Blue solution for 20 min. The gel was destained with a solution containing 10% acetic acid and 10% methanol until the required level of gel clarity was achieved.

### Enzyme Assays

As reported earlier, AbIspC and KpIspC activity was followed by monitoring NADPH oxidation at 340 nm via an Agilent 8453 UV-vis spectrophotometer with a temperature-controlled cuvette holder.^9^ Enzyme assay conditions included 100 mM Tris-HCl buffer (pH 7.8), 25 mM MgCl_2_ 150 μM NADPH, 0.5% DMSO (v/v), and either 126 μM or 155 μM DXP (Echelon Biosciences, Salt Lake City, UT) for AbIspC or KpIspC assays, respectively, and 84 ng IspC in a 120 μL final assay volume. Assays were performed at 37 °C. The addition of DXP initiated reactions. A no-substrate blank was included with all assays to measure the rate of uncatalyzed NADPH oxidation and to verify that the inhibitor absorption spectra did not overlap with that of NADPH at 340 nm. We performed the initial (screening) enzyme inhibition assays by treating AbIspC and KpIspC with 100 μM of each IspC inhibitor (compound). For the compounds showing 90% or more inhibition of enzyme activity, the half-maximal inhibitory concentration (IC_50_) value was determined using standard dose−response plots. IC_50_ values were determined iteratively by two enzymologists using at least three independent biological replicates. The first enzymologist generated a provisional IC_50_ from duplicate inhibition assays. A second enzymologist validated this value using an independent replicate with the inhibitor fixed at the provisional IC_50_. Values yielding 50 ± 5% enzyme activity were accepted; otherwise, the first enzymologist repeated the assays to refine the IC_50_, followed by revalidation until acceptance criteria were met. GraphPad Prism 8 for Windows (GraphPad Software, San Diego, CA) was used for enzyme inhibition assay data analysis and generation of IC_50_ curves.

### Antimicrobial Susceptibility Test (AST)

AST assays were performed as reported in our previous work.^9^ Clinical isolates of *Ab* strains AB5075, AB5711, AB3560, AB3638, AB0087, AB4052, AB5674, AB0967, AB4795, AB4957, AB585, AB499, and *Kp* strain KP4640 were obtained from the Multidrug-resistant Organism Repository and Surveillance Network (MRSN) at Walter Reed Army Institute of Research. AB19606 and KP43816 were obtained from the American Type Culture Collection (ATCC).^48^ To determine the compounds’ minimum inhibitory concentration (MIC), we used the broth microdilution method, following the guidelines presented by the Clinical and Laboratory Standards Institute (CLSI).^49^ Fosmidomycin and FR900098 were purchased from Sigma-Aldrich (St. Louis, MO). All the inhibitors were dissolved in DMSO. The compounds were 2-fold diluted in cation-adjusted Mueller Hinton Broth (CAMHB) in 96-well round-bottom polystyrene microtiter plates. Assays were done in triplicate, where each well contained 100 μL drug-containing broth. The growth and the sterility control wells had broth media only (i.e., without the drugs). *Ab* and Kp strains were cultured on blood agar plates at 37 °C for 16−18 h. Colonies were taken from the plate and mixed with PBS. The optical density at 600 nm was adjusted to 0.1 using a spectrophotometer to get about 1 × 10^8^ colony-forming units (CFU)/mL. The bacterial suspension was diluted by a factor of 10, and then 5 μL of the bacteria was added to each well to have an initial inoculum of approximately 5 × 10^5^ CFU/mL. The 96-well plates were incubated at 37 °C for 20 h. Then, the MIC was determined as the lowest concentration that prevented bacterial growth. Assays were performed in triplicate at each inhibitor concentration. MIC values were defined as the lowest inhibitor concentration that completely prevented bacterial growth in all three replicate wells, rather than as modal or median values from triplicate assays.

### Crystallization

Crystallization experiments were set up with 29 mg/mL AbIspC protein according to conditions reported previously by the Seattle Structural Genomics Center for Infectious Disease (SSGCID) (PDB ID: 4ZN6) and our group (PDB ID: 7S04).^9^ Crystals were grown by vapor diffusion in 24-well Limbro sitting drop plates at 16 °C. Precipitant conditions of crystals yielding structures were composed of 100 mM sodium citrate, pH 5.6, 200−225 mM ammonium sulfate, and 21−22% PEG 4000. The crystals were transferred to sitting drop solutions containing ammonium chloride instead of ammonium sulfate, with every other component being the same, to remove the sulfate, which could potentially interfere with inhibitor binding at the active site. Resulting crystals were soaked in solutions containing the precipitant condition, 10 mM compound of interest, 10 mM MgCl_2_, and 1 mM NADPH. Crystals were cryoprotected first in a solution containing the precipitant well solution plus 10% ethylene glycol and a second solution containing the precipitant well solution plus 15% ethylene glycol, then flash frozen in liquid nitrogen in preparation for data collection.

### Data Collection and Structure Determination of AbIspC Bound to Compounds **2b**, **3b**, and **4b**

Data were collected at GM/CA beamline 23-ID-D at the Advanced Photon Source at Argonne National Laboratory (Lemont, IL). Data were indexed, integrated and scaled using XDS and AIMLESS.^50,51^ Complete data collection statistics can be found in Table 4. Phases were solved by molecular replacement with PHASER^52^ using AbIspC structure bound to FR as the search model (PDB ID: 7S04)^9^ with waters and ligands removed. Data between 40 Å and 2.0 Å resolution were used for refinement for all three data sets. Multiple rounds of model building in Coot^53^ and refinement with PHENIX^54^ were carried out to the final R-work/R-free statistics shown in Table 4. The CheckMyMetal^55^ server was also used to aid model building and validation. Figures were generated with ChimeraX.^56^ Structural alignments were performed and RMSD values were calculated in ChimeraX with the Matchmaker tool. Two-dimensional small-molecule binding figures (Figure 7) were generated with LigPlot+.^57^ Diffraction data were truncated at 2.0 Å for refinement based on high-resolution shell statistics (I/σI, CC1/2, and completeness). Paired refinements with and without data beyond 2.0 Å yielded indistinguishable ligand electron-density features (for example, similar F_o_−F_c_ and 2F_o_−F_c_ maps and ligand RSCC), so the final model was refined against data to 2.0 Å.

## Supplementary Material





## Notes

Molecular formula strings (XLSX)
Multiple sequence alignments of the amino acid residues of the IspC homologues; IC_50_ curves of the IspC inhibitors; etc. (PDF)
