# Supplementary material for: Structure−Activity Relationships among Inhibitors of Acinetobacter baumannii and Klebsiella pneumoniae 1-Deoxy-d-xylulose 5-Phosphate Reductoisomerase (DXR/IspC) − A Promising Target for Antibiotic Development
Source: ACS Infect Dis. 2026 Mar 12;12(7):2203–15. doi: 10.1021/acsinfecdis.5c00875 (PMC13366575; doi:10.1021/acsinfecdis.5c00875)
Supplement: Supplementary file 2 [file id5c00875_si_002.pdf]

Supporting Information

Structure-Activity Relationships Among Inhibitors of *Acinetobacter baumannii* and *Klebsiella pneumoniae* 1-Deoxy-D-Xylulose 5-Phosphate Reductoisomerase (DXR/IspC) – a Promising Target for Antibiotic Development.

Misgina Girma, Meagan Belcher Dufrisne, Archi Sehgal, Allyson Dailey, Kenneth Heidel, Darean Bague, Xu Wang, Logan Bartholomew, Richard Beck, Samuel Kirby, Haley Ball, Mosufa Zainab, Soo Hyeon Lee, Iswarduth Soojhawon, Schroeder M. Noble, Cynthia S. Dowd, Robin D. Couch*

*Corresponding author, Department of Chemistry and Biochemistry, George Mason University, Fairfax, VA 22030-4444, United States of America. Email: rcouch@gmu.edu.

Analytical data for tested compounds:

Diammonium [(1E)-3-(N-hydroxy-1-phenylformamido)prop-1-en-1-yl]phosphonate (1b). ^{1}H NMR (400 MHz, CD_3OD) δ 7.86 – 7.36 (m, 5H), 6.65 – 6.48 (m, 1H), 6.18 – 6.02 (m, 1H), 4.57 – 4.30 (m, 2H). ^{13}C NMR (151 MHz, CD_3OD) δ 171.65, 140.20, 135.27, 131.77, 129.17, 127.44, 126.27, 53.36. HRMS (ESI+) calculated for $C_{10}H_{13}NO_5P$: 258.0531; found 258.0526 $[M+H]^+$.

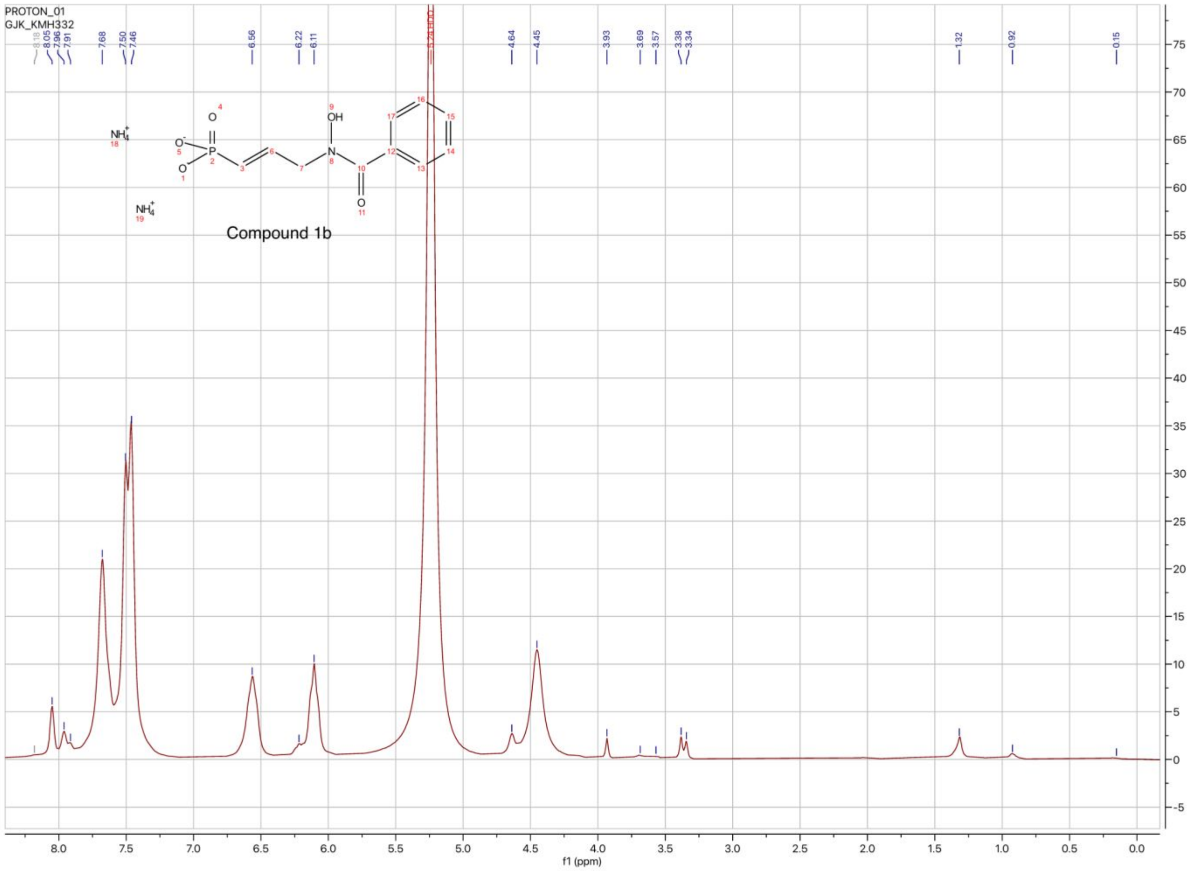

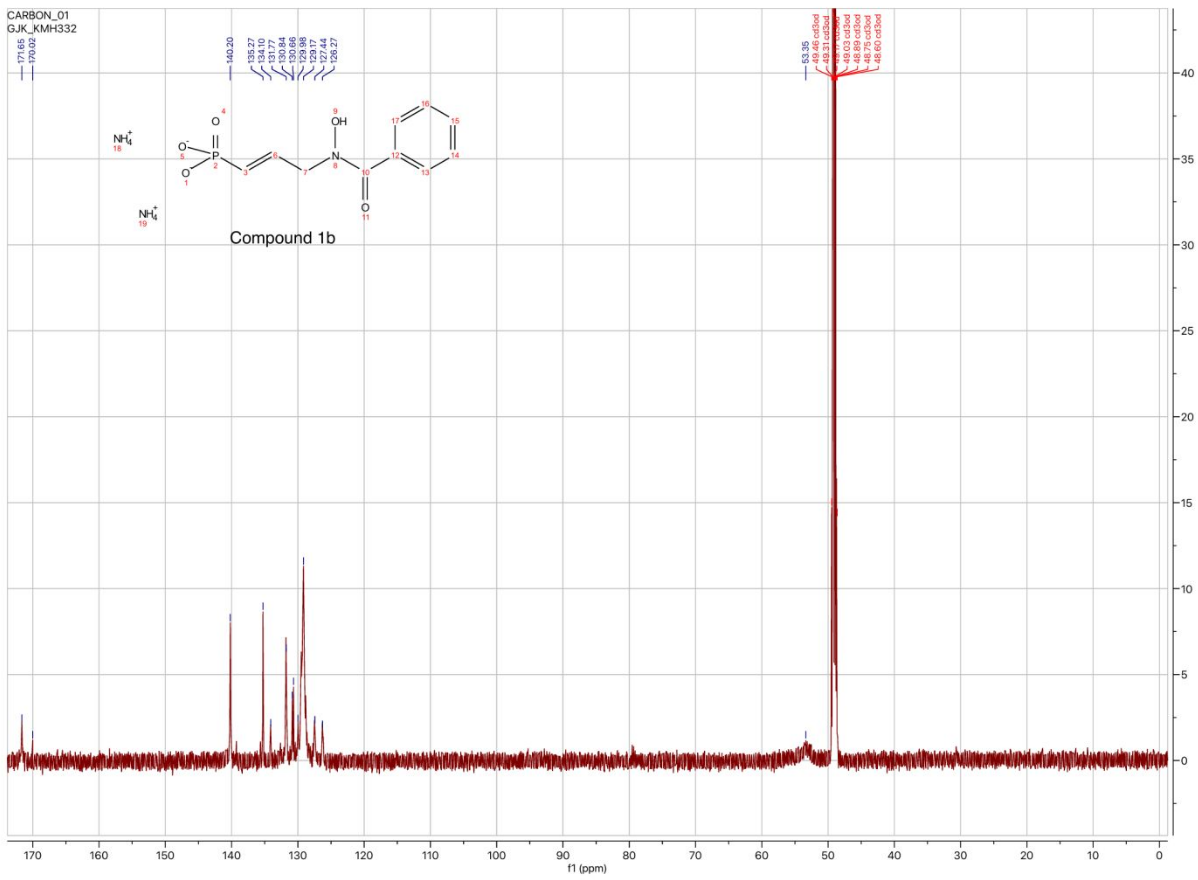

CARBON_01
GJK_KMH332
Compound 1b
f1 (ppm)

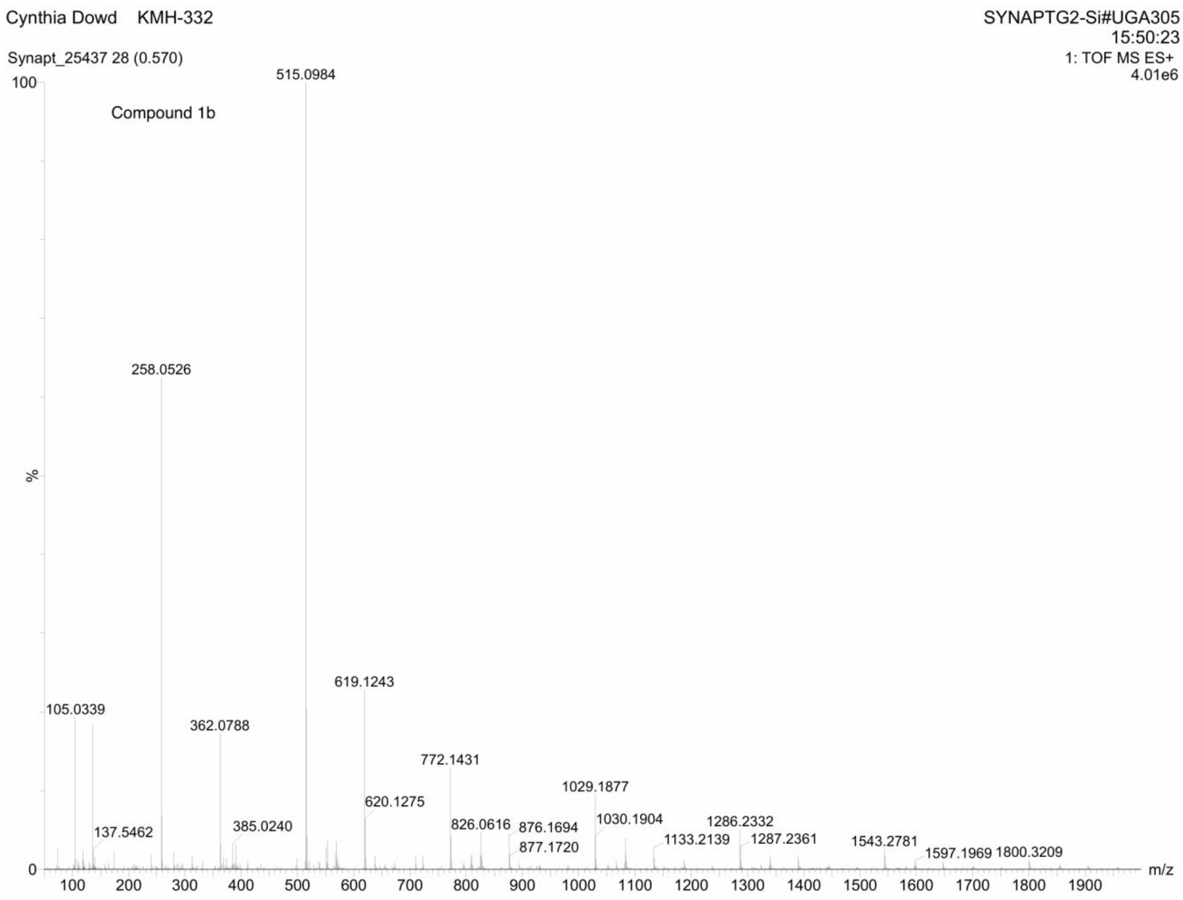

Diammonium [(2R)-2-hydroxy-3-(N-hydroxyacetamido)propyl]phosphonate (1c). ^{1}H NMR (400 MHz, CD_3OD) δ 4.32 – 4.17 (m, 1H), 3.84 – 3.57 (m, 2H), 2.12 (s, 3H), 1.91 – 1.70 (m, 2H). ^{13}C NMR (101 MHz, CD_3OD) δ 172.80 (s), 64.87 (s), 54.32 (d, J = 13.2 Hz), 33.97 (d, J = 131.4 Hz), 19.03 (s). LC-MS (ESI$^-$): 212.0 m/z [M-2NH$_4$+H]$^-$. HRMS (ESI$^-$) calculated for $C_5H_{18}N_3O_6P$, 247.0933; found, 212.0326 [M-2NH$_4$+H]$^-$. $[\alpha]_{D}^{25}$ +3.2 (c 1.2, CH_3OH).

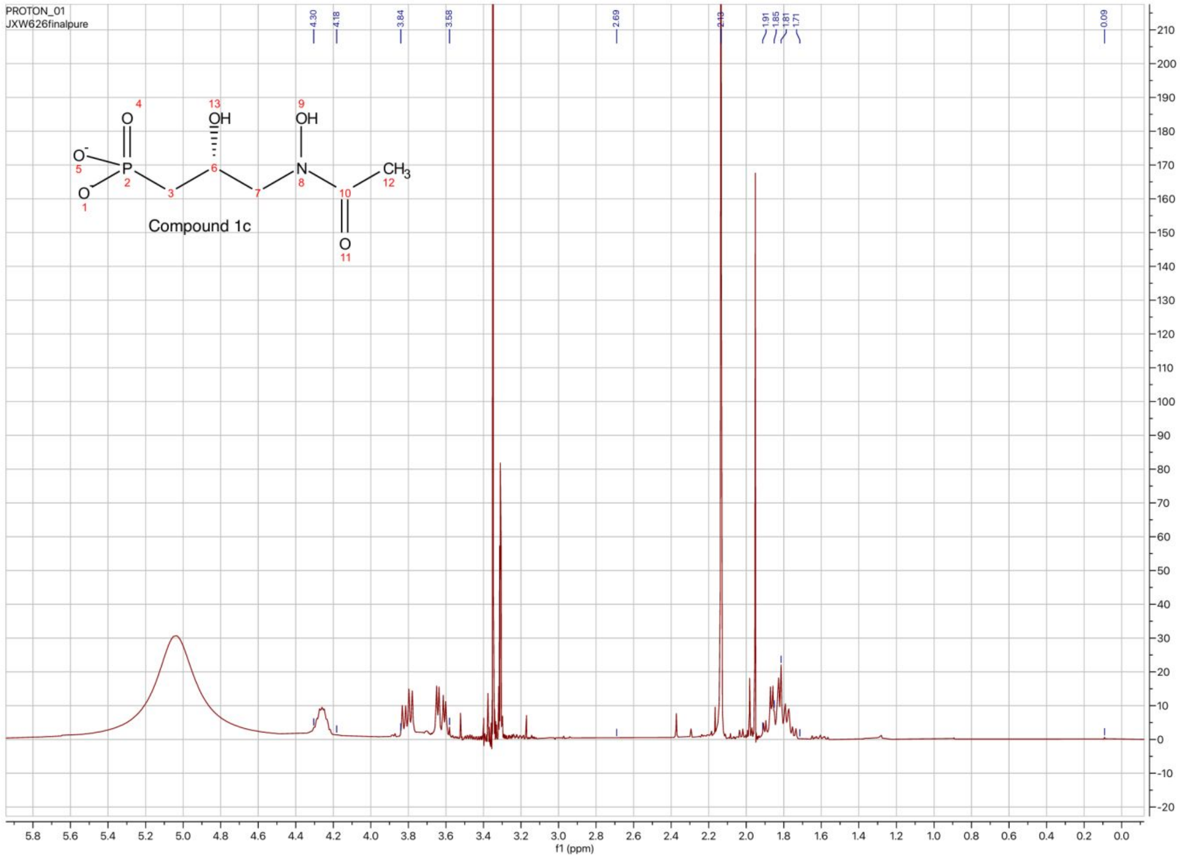
PROTON_01
JXW626finalpure
4.30
4.18
3.84
3.58
2.69
2.13
1.91
1.85
1.81
1.71
0.09
Compound 1c
f1 (ppm)

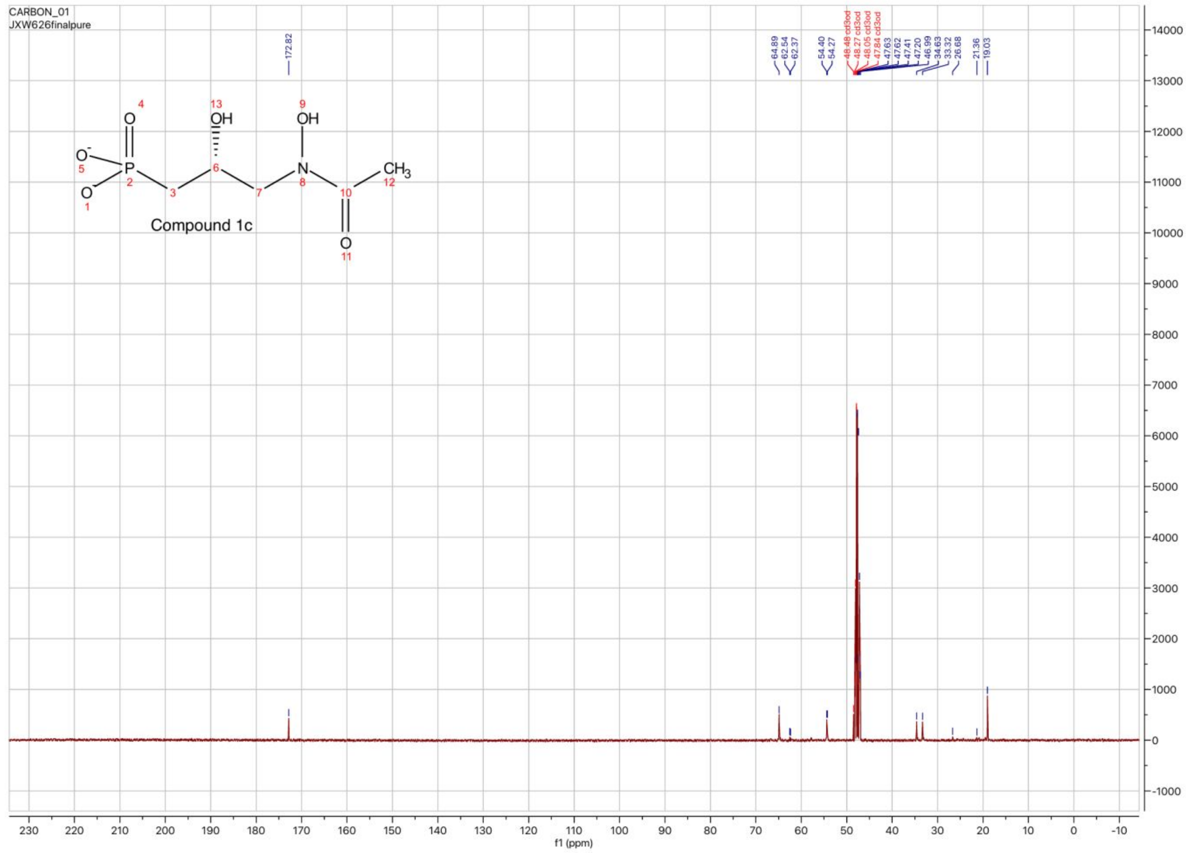

CARBON_01
JXW626finalpure
172.82
64.89
62.54
62.37
54.40
54.27
19.03
Compound 1c
f1 (ppm)

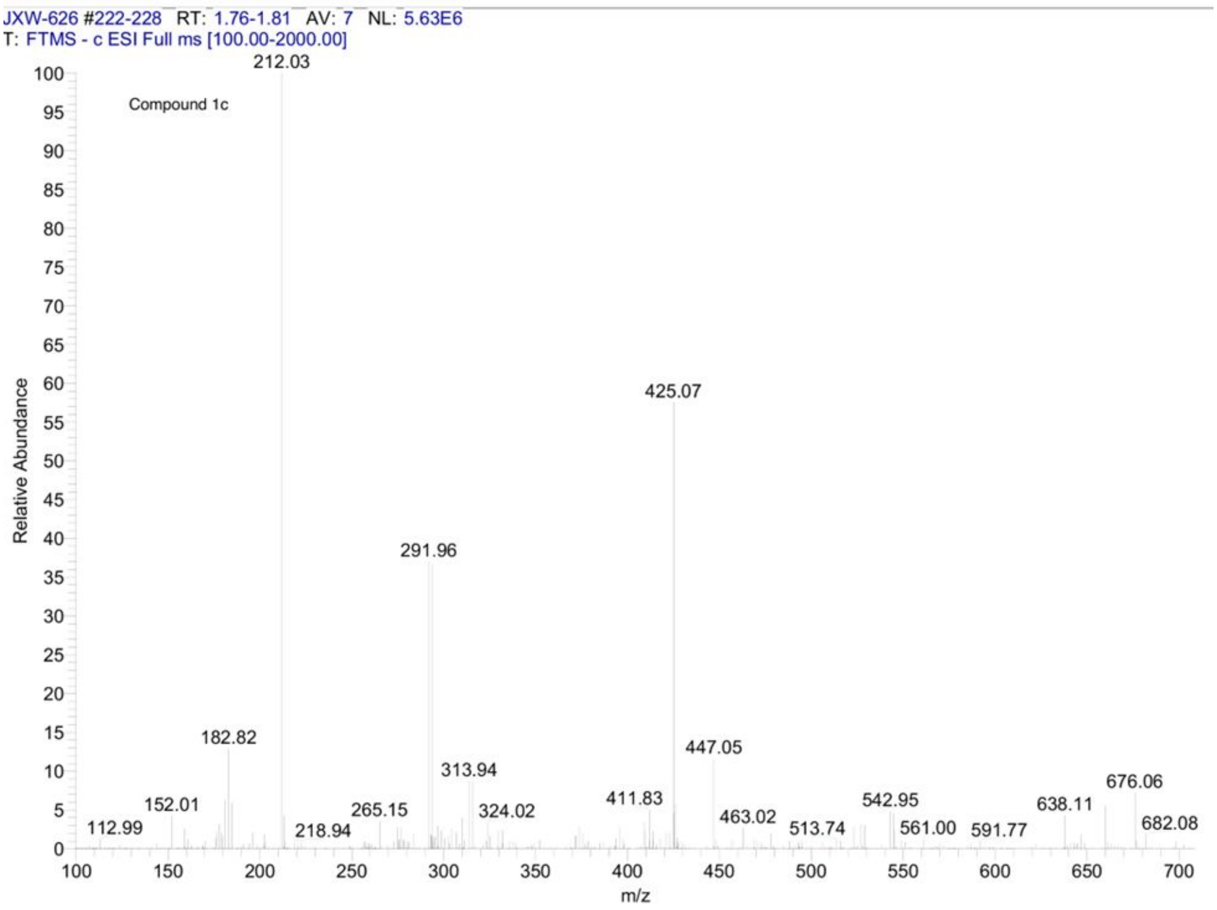

Sodium hydrogen [(1E)–3–[1–(3–chlorophenyl)–N–hydroxyformamido] prop–1–en–1–yl] phosphonate (2b). 1H NMR (400 MHz, DMSO) δ 7.93 – 7.63 (m, 2H), 7.50 – 7.34 (m, 2H), 6.40 – 6.14 (m, 1H), 5.86 (t, J = 16.1 Hz, 1H), 4.26 (s, 2H). ^{13}C NMR (101 MHz, CD_3OD) δ 169.99, 137.75, 136.84, 131.77, 131.61, 131.20, 130.44, 130.04, 128.65. LCMS (ESI^-): 290, 292 *m/z* $[M-H]^-$. HRMS (ESI^+) calculated for $C_{10}H_{10}ClNO_5P$: 289.9985; found 289.9998 $[M-H]^-$.

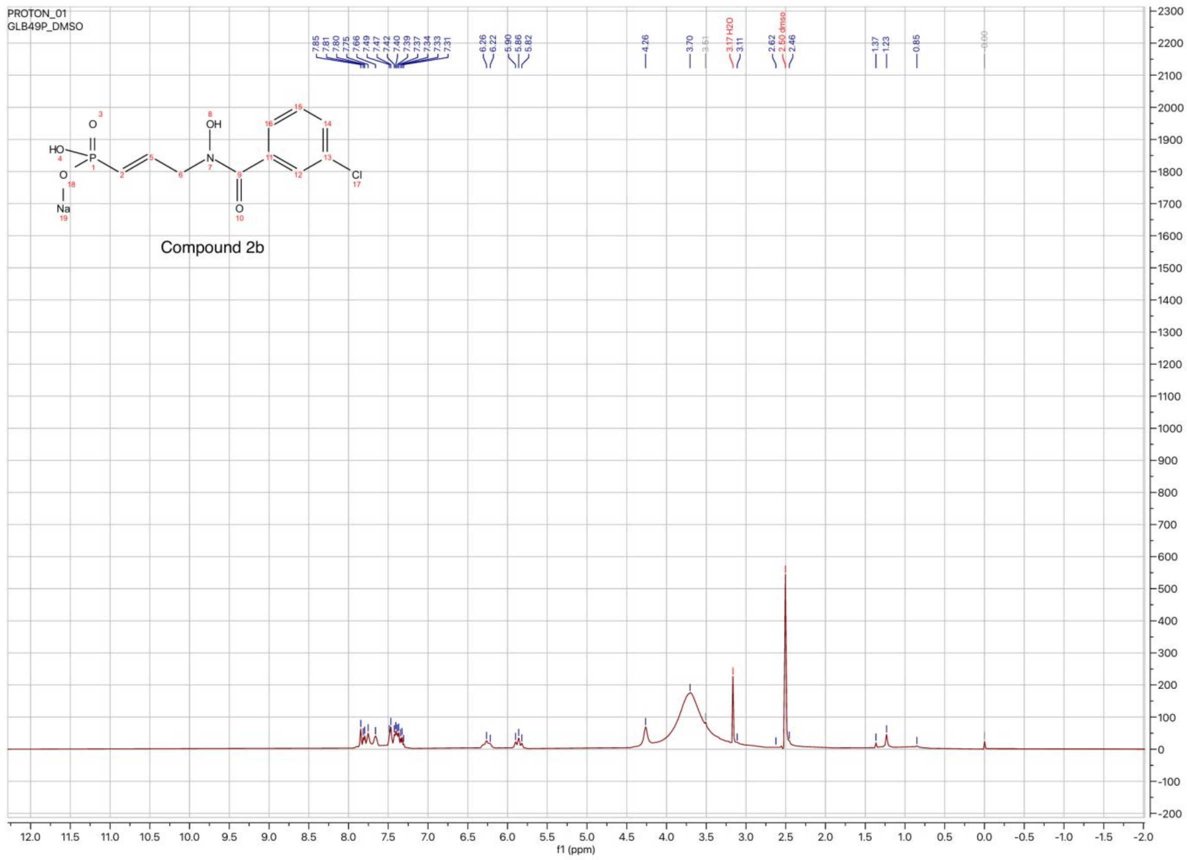
PROTON_01
GLB49P_DMSO
Compound 2b
f1 (ppm)
3.17 H2O
2.50 dmso

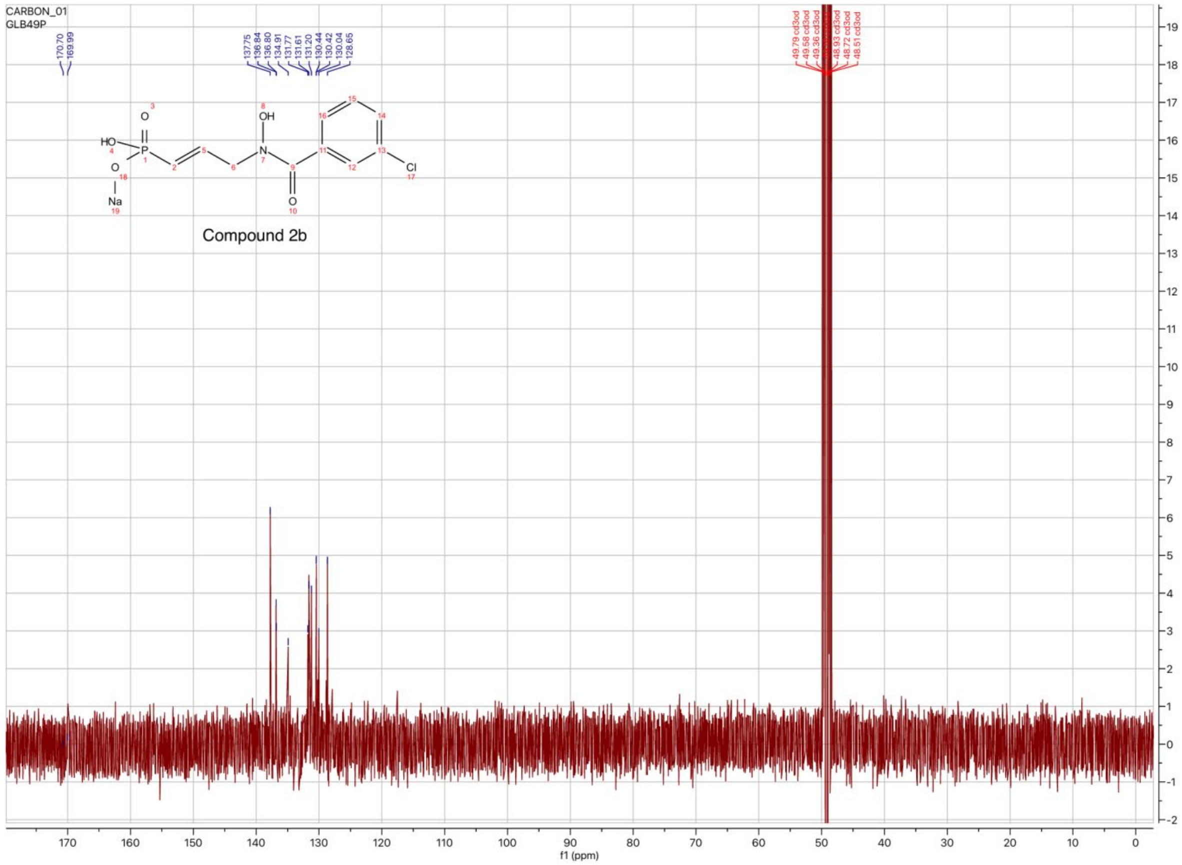
CARBON_01
GLB49P
170.70
169.99
137.75
136.84
136.80
134.91
131.77
131.61
131.20
130.44
130.42
130.04
128.65
49.79 cd3od
49.58 cd3od
49.36 cd3od
48.93 cd3od
48.72 cd3od
48.51 cd3od
Compound 2b
f1 (ppm)

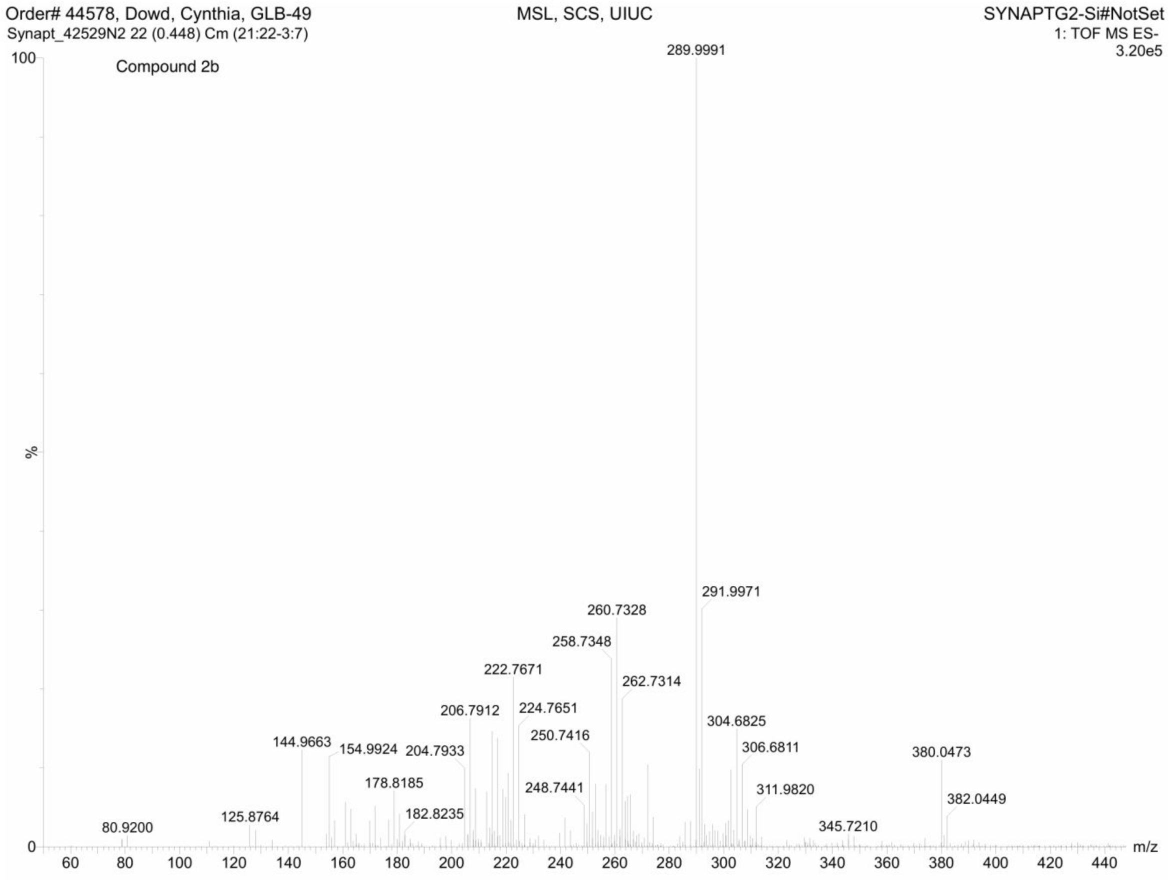

Diammonium [(2S)-2-hydroxy-3-(N-hydroxyacetamido)propyl]phosphonate (2c). ^{1}H NMR (400 MHz, CD_3OD) δ 4.32 – 4.17 (m, 1H), 3.84 – 3.57 (m, 2H), 2.12 (s, 3H), 1.91 – 1.70 (m, 2H). ^{13}C NMR (101 MHz, CD_3OD) δ 172.80 (s), 64.87 (s), 54.32 (d, J = 13.2 Hz), 33.97 (d, J = 131.4 Hz), 19.03 (s). LC-MS (ESI$^-$): 212.0 m/z HRMS (ESI$^-$) calculated for $C_5H_{18}N_3O_6P$, 247.0933; found, 212.0326 $[M-2NH_4+H]^-$. $[\alpha]_{D}^{25}$ -3.5 (c 0.7, CH_3OH).

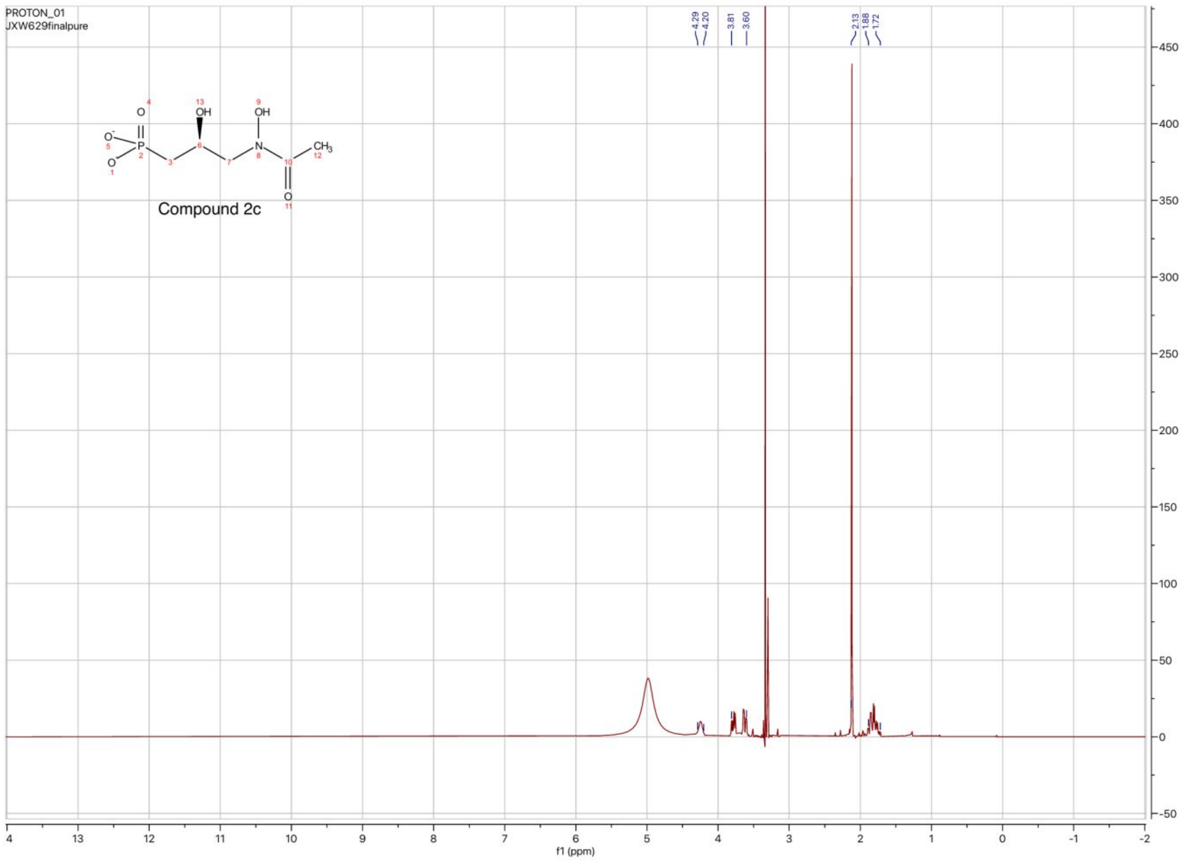
PROTON_01
JXW629finalpure
Compound 2c
4.29
4.20
3.81
3.60
2.13
1.88
1.72
f1 (ppm)

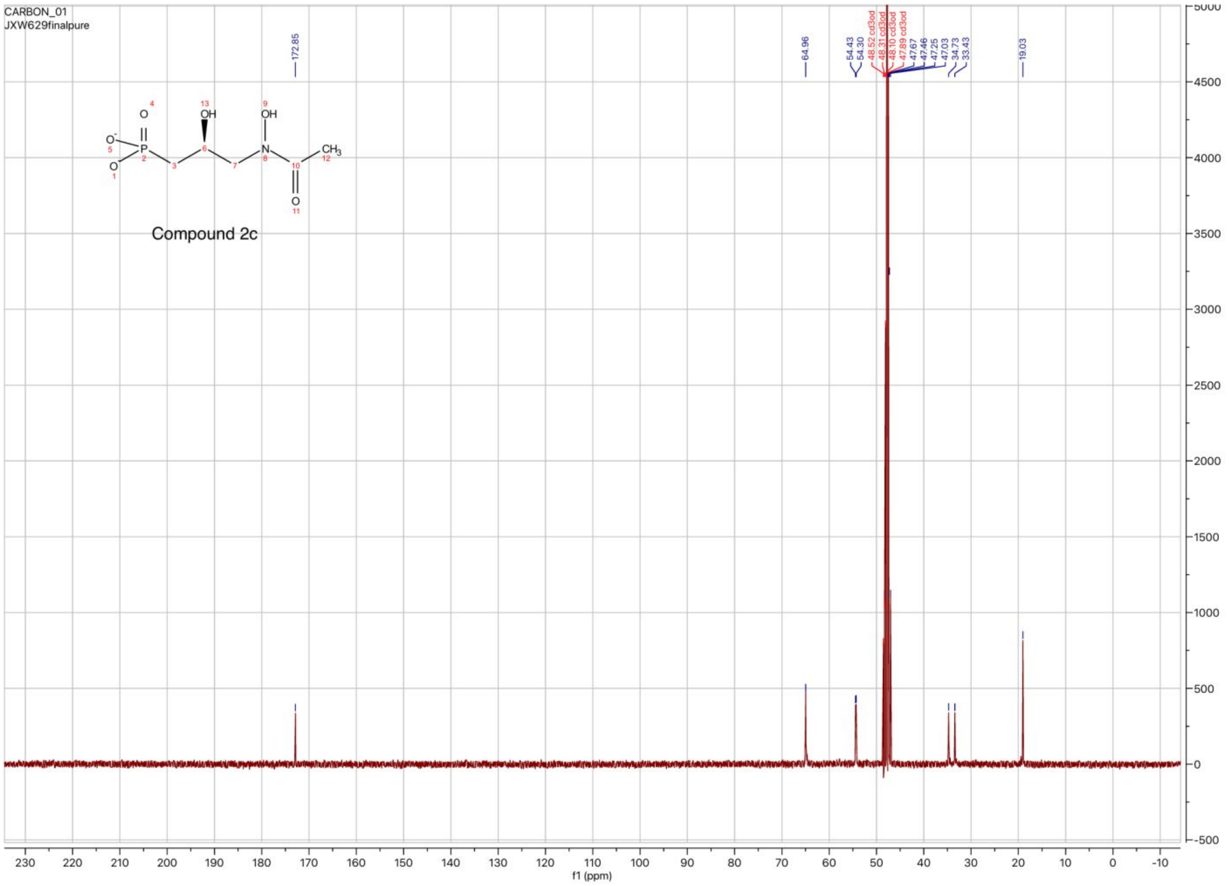
CARBON_01
JXW629finalpure
Compound 2c
172.85
64.96
54.43
54.30
48.52 cd3od
47.89 cd3od
47.67
47.46
47.25
47.03
34.73
33.43
19.03
f1 (ppm)

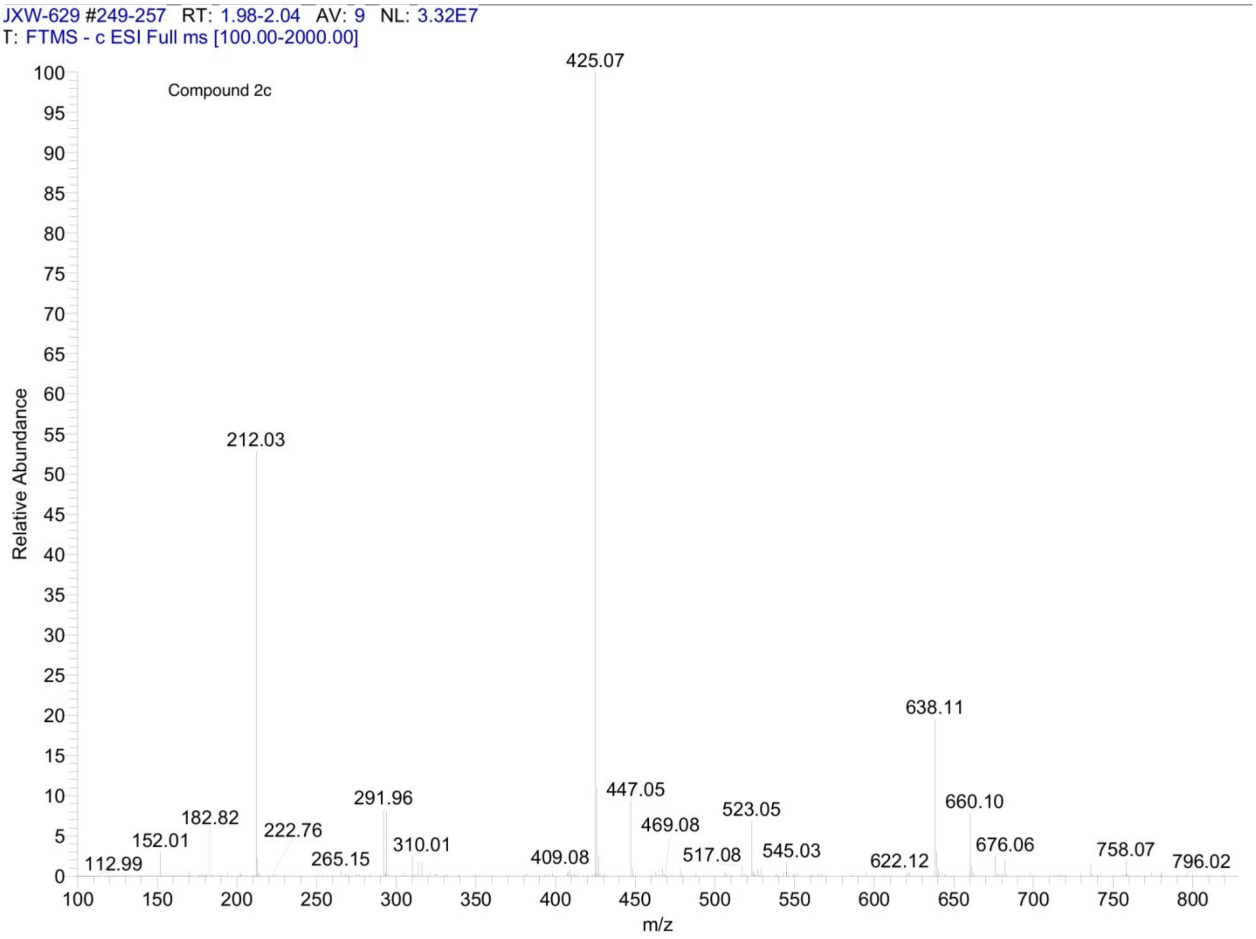

Sodium hydrogen [3-(N-hydroxy-1-phenylformamido)propyl]phosphonate (3a). ^{1}H NMR (400 MHz, D_2O) δ 7.54 – 7.29 (m, 5H), 3.85 – 3.41 (m, 2H), 1.98 – 1.73 (m, 2H), 1.71 – 1.33 (m, 2H). ^{13}C NMR (101 MHz, D_2O) δ 171.76, 133.33, 130.73, 128.49, 127.01, 49.43, 25.36, 20.95. HRMS (ESI$^+$) calculated for $C_{10}H_{15}NO_5P$: 260.0688; found 260.0685 $[M+H]^+$.

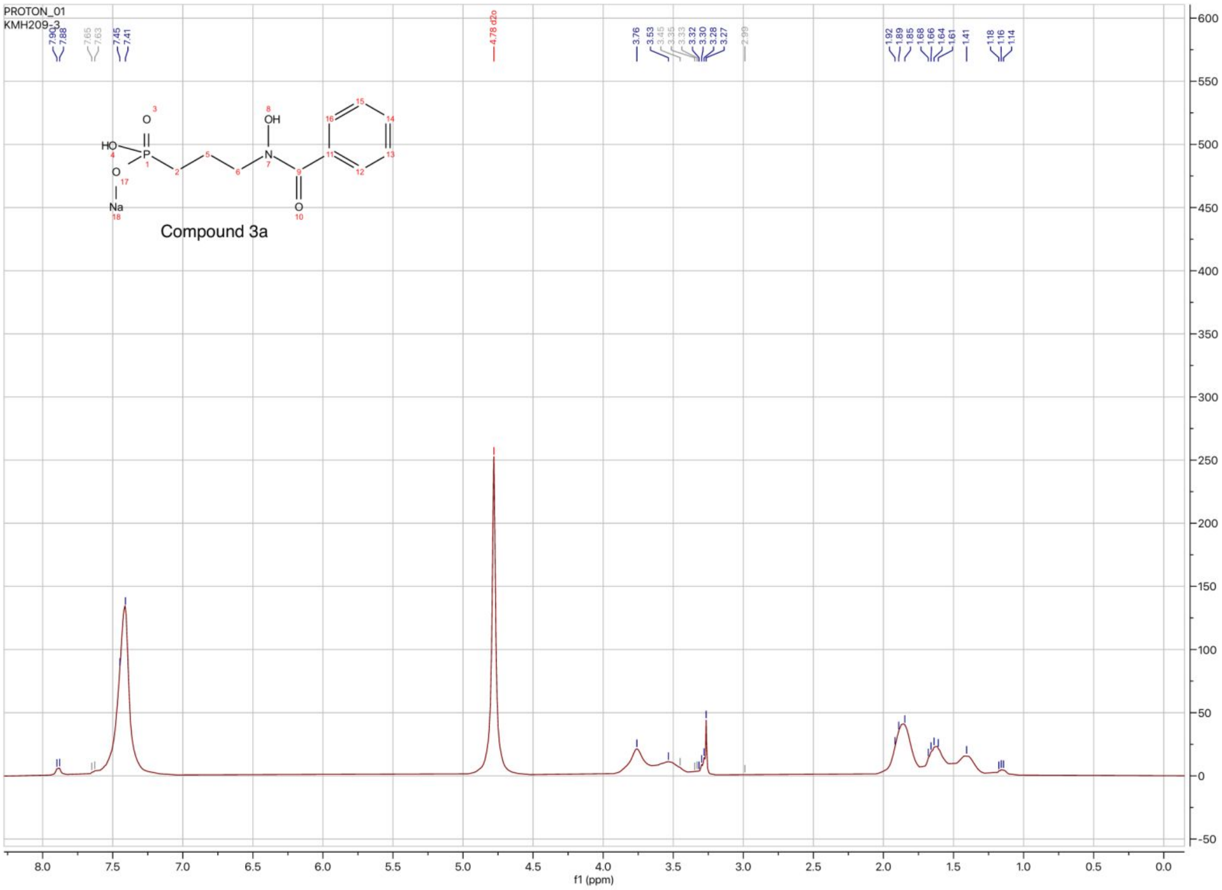
PROTON_01
KMH209-3
7.90
7.88
7.65
7.63
7.45
7.41
4.78 d2o
3.76
3.53
3.45
3.35
3.33
3.32
3.30
3.28
3.27
2.99
1.92
1.89
1.85
1.68
1.66
1.64
1.61
1.41
1.18
1.16
1.14
Compound 3a
f1 (ppm)

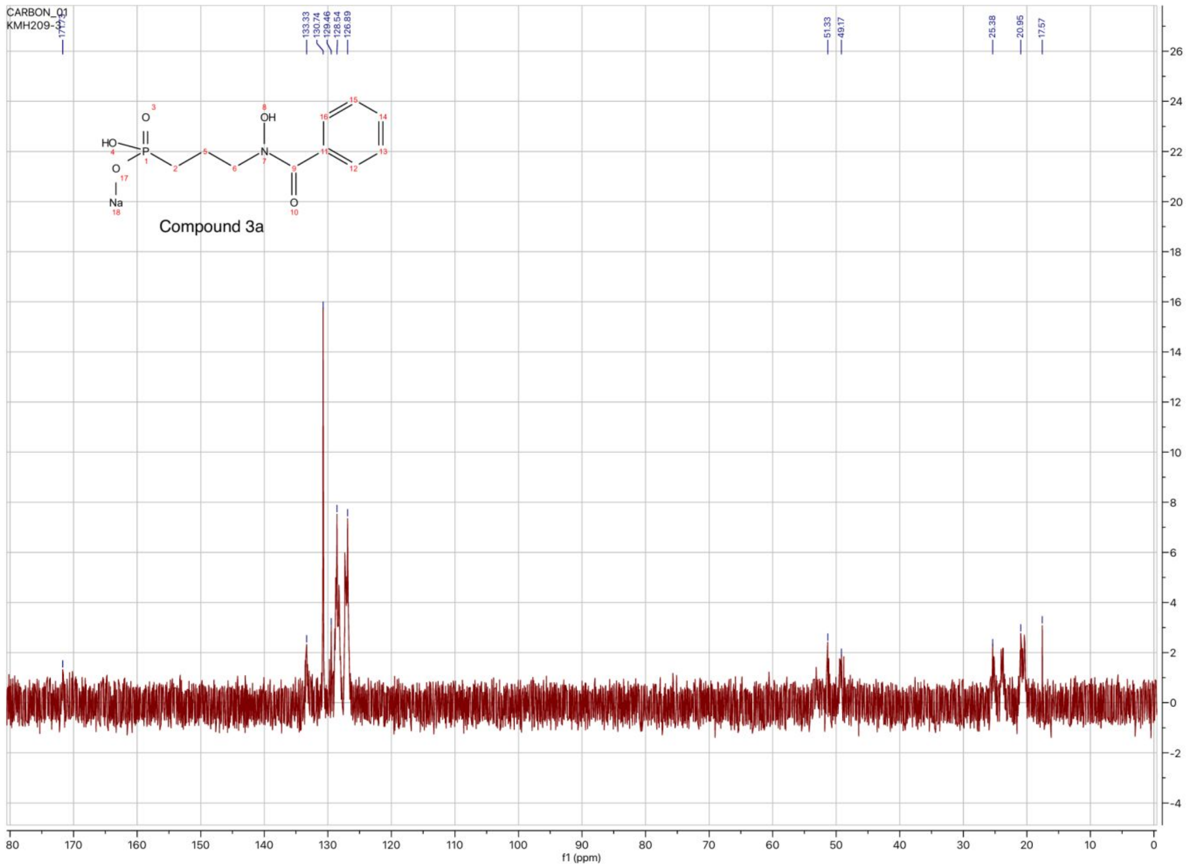
CARBON_01
KMH209-3
171.
133.33
130.74
129.46
128.54
126.89
51.33
49.17
25.38
20.95
17.57
Compound 3a
f1 (ppm)

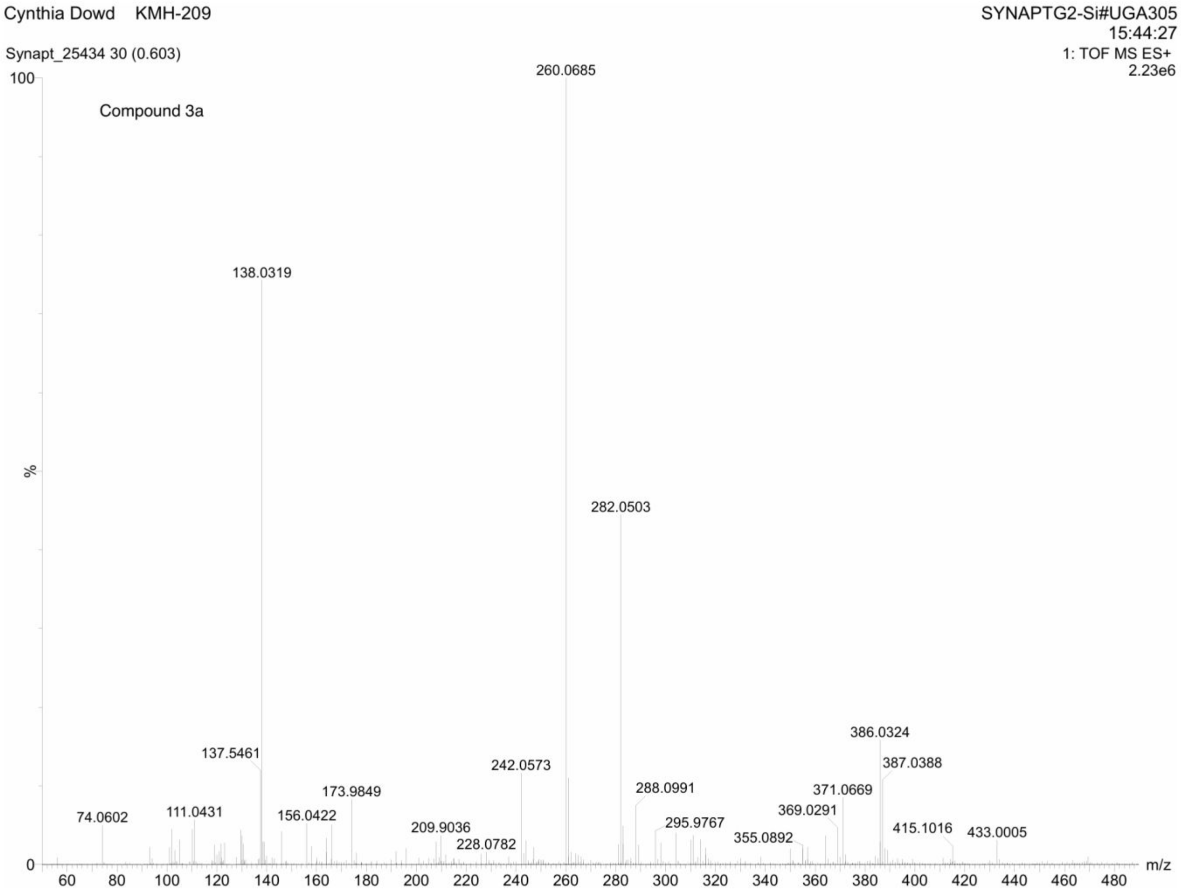

Sodium hydrogen [(1E)–3–[1–(4–chlorophenyl)–N–hydroxyformamido]prop–1–en–1–yl] phosphonate (3b). ^{1}H NMR (400 MHz, CD_3OD) δ 7.71 (d, *J* = 97.1 Hz, 2H), 7.27 (d, *J* = 16.9 Hz, 2H), 6.60 – 6.19 (m, 1H), 6.22 – 5.86 (m, 1H), 4.54 – 3.70 (m, 2H). ^{13}C NMR (101 MHz, CD_3OD) δ 170.15, 137.84, 137.46, 133.83, 132.02, 131.45, 130.34, 129.13, 128.93, 53.06. LCMS (ESI$^-$): 290, 292 *m/z* [M-H]$^-$. HRMS (ESI$^+$) calculated for $C_{10}H_{10}ClNO_5P$: 289.9985; found 289.9996 [M-H]$^-$.

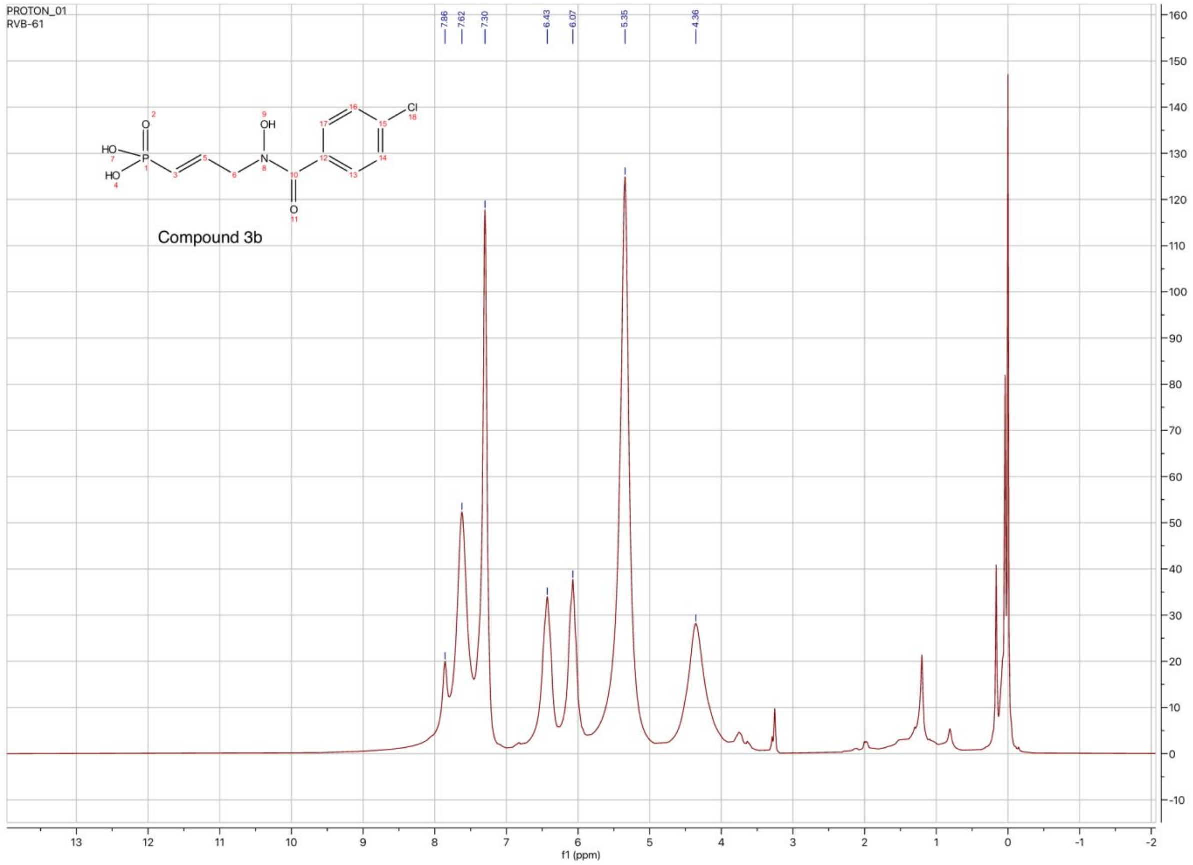

PROTON_01
RVB-61
7.86
7.62
7.30
6.43
6.07
5.35
4.36
Compound 3b
f1 (ppm)

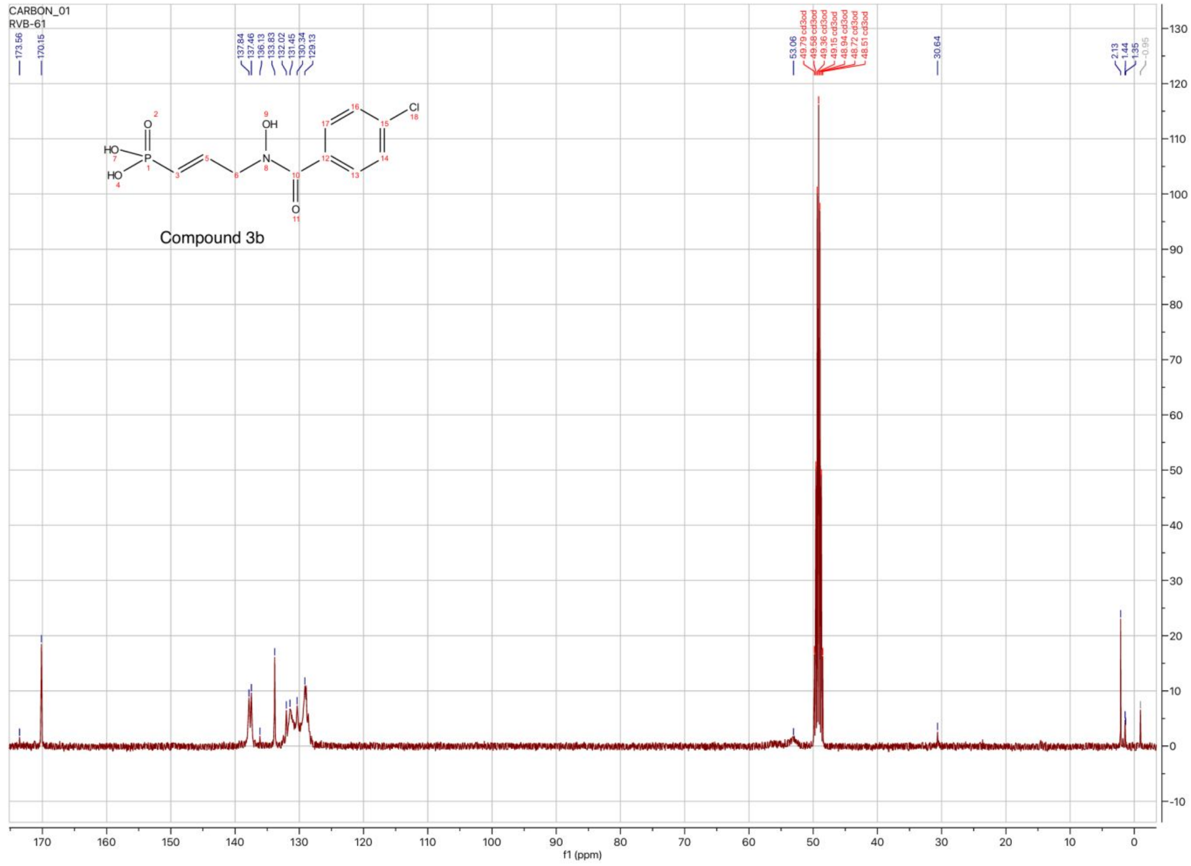

CARBON_01
RVB-61
173.56
170.15
137.84
137.46
136.13
133.83
132.02
131.45
130.34
129.13
53.06
49.79 cd3od
49.58 cd3od
49.36 cd3od
49.15 cd3od
48.94 cd3od
48.72 cd3od
48.51 cd3od
30.64
2.13
1.44
1.35
-0.95
Compound 3b
f1 (ppm)

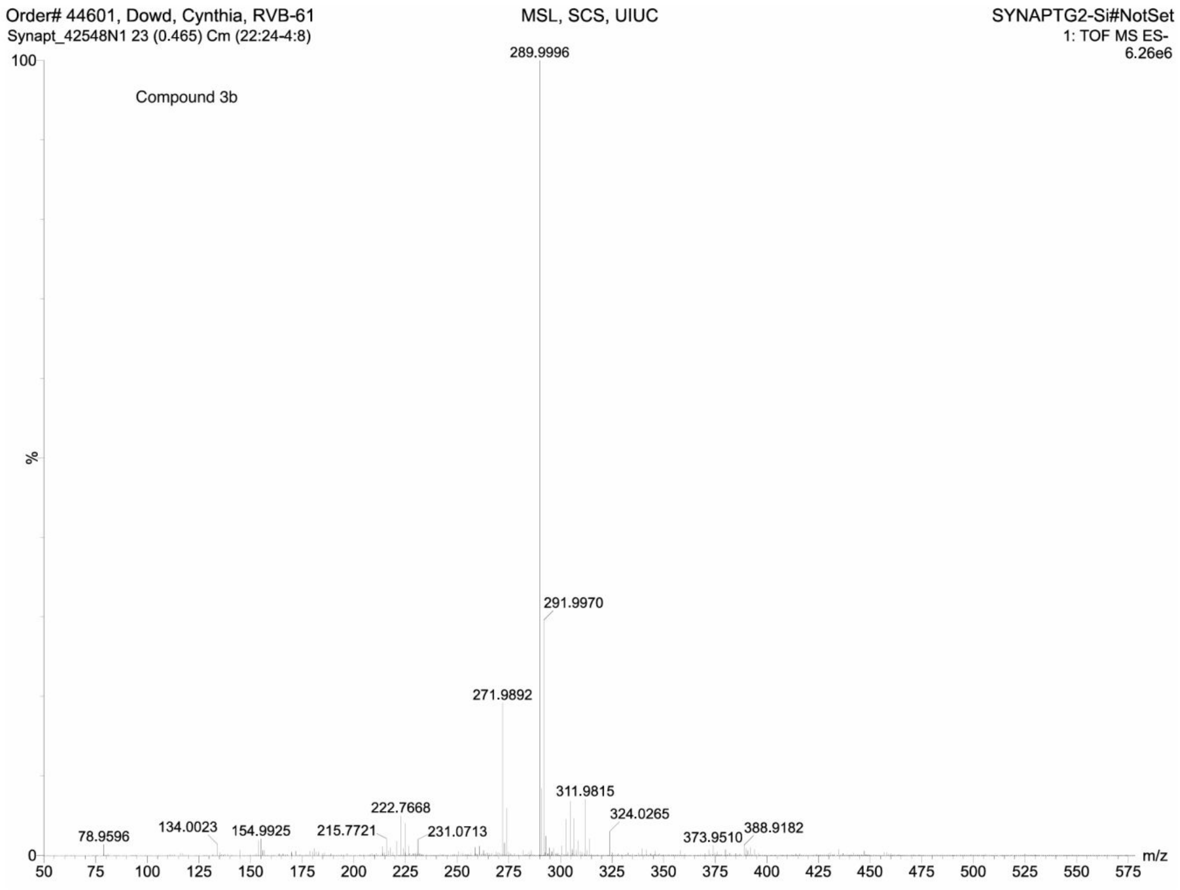

Sodium hydrogen [(1E)–3–[1–(3,5–dichlorophenyl)–N–hydroxyformamido] prop–1–en–1–yl] phosphonate (4b). ^{1}H NMR (400 MHz, DMSO) δ 7.77 (d, J = 2.0 Hz, 1H), 7.69 (s, 1H), 7.62 (s, 1H), 6.39 – 6.23 (m, 1H), 5.87 (t, J = 16.4 Hz, 1H), 4.27 (s, 2H). ^{13}C NMR (101 MHz, DMSO) δ 165.39, 138.38, 133.43, 133.32, 129.31, 127.70, 127.27, 51.09. LCMS (ESI$^-$): 324, 326 *m/z* [M-H]$^-$. HRMS (ESI$^+$) calculated for $C_{10}H_9Cl_2NO_5P$: 323.9595; found 323.9608 [M-H]$^-$.

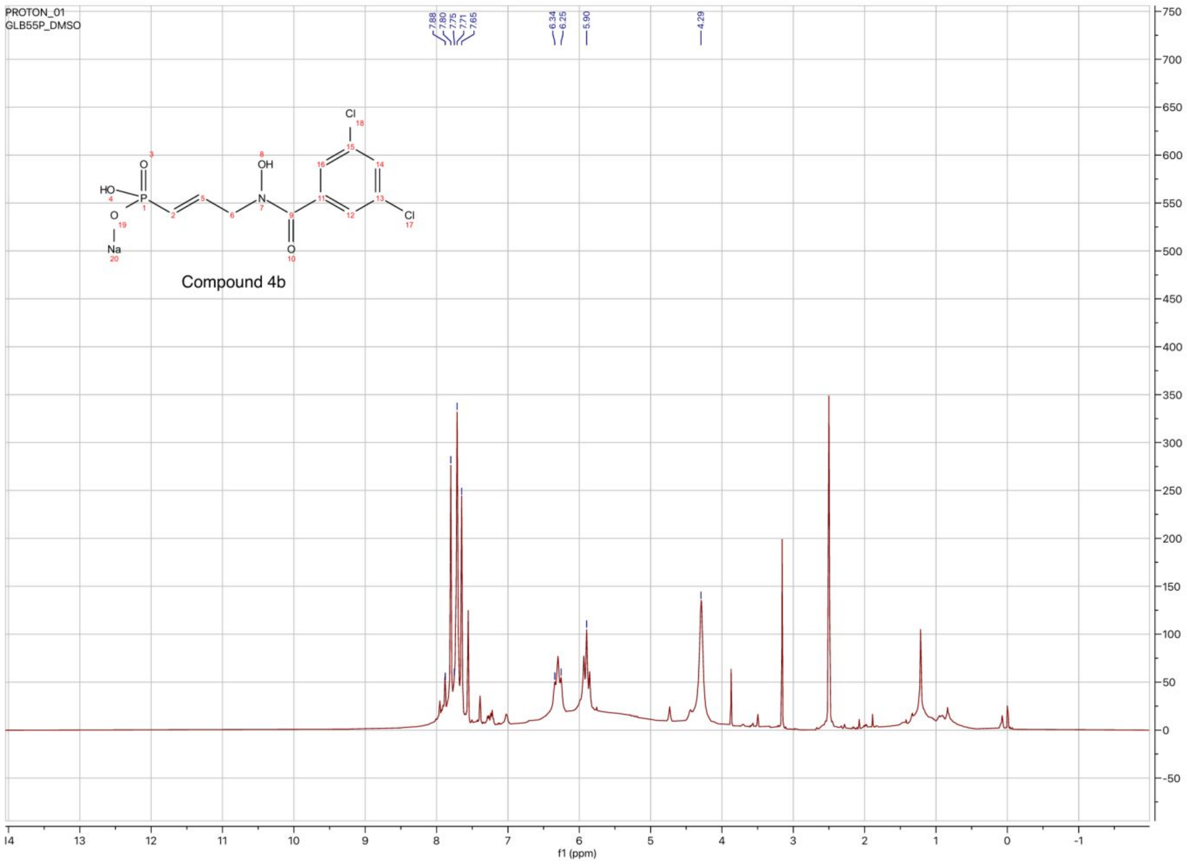

PROTON_01
GLB55P_DMSO
7.88
7.80
7.75
7.71
7.65
6.34
6.25
5.90
4.29
Compound 4b
f1 (ppm)

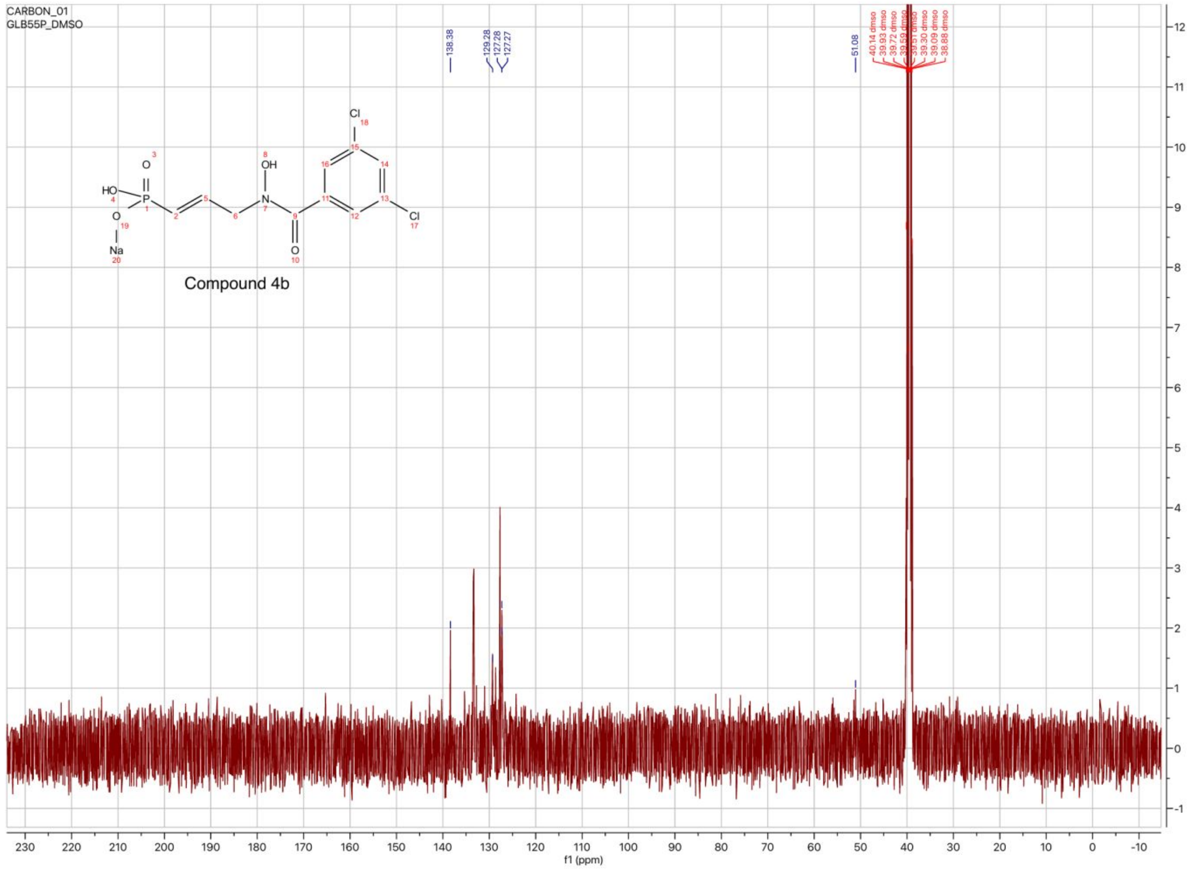

CARBON_01
GLB55P_DMSO
138.38
129.28
127.28
127.27
51.08
40.14 dmso
39.93 dmso
39.72 dmso
39.30 dmso
39.09 dmso
38.88 dmso
Cl
O
OH
HO
P
N
O
Na
Cl
O
Compound 4b
f1 (ppm)

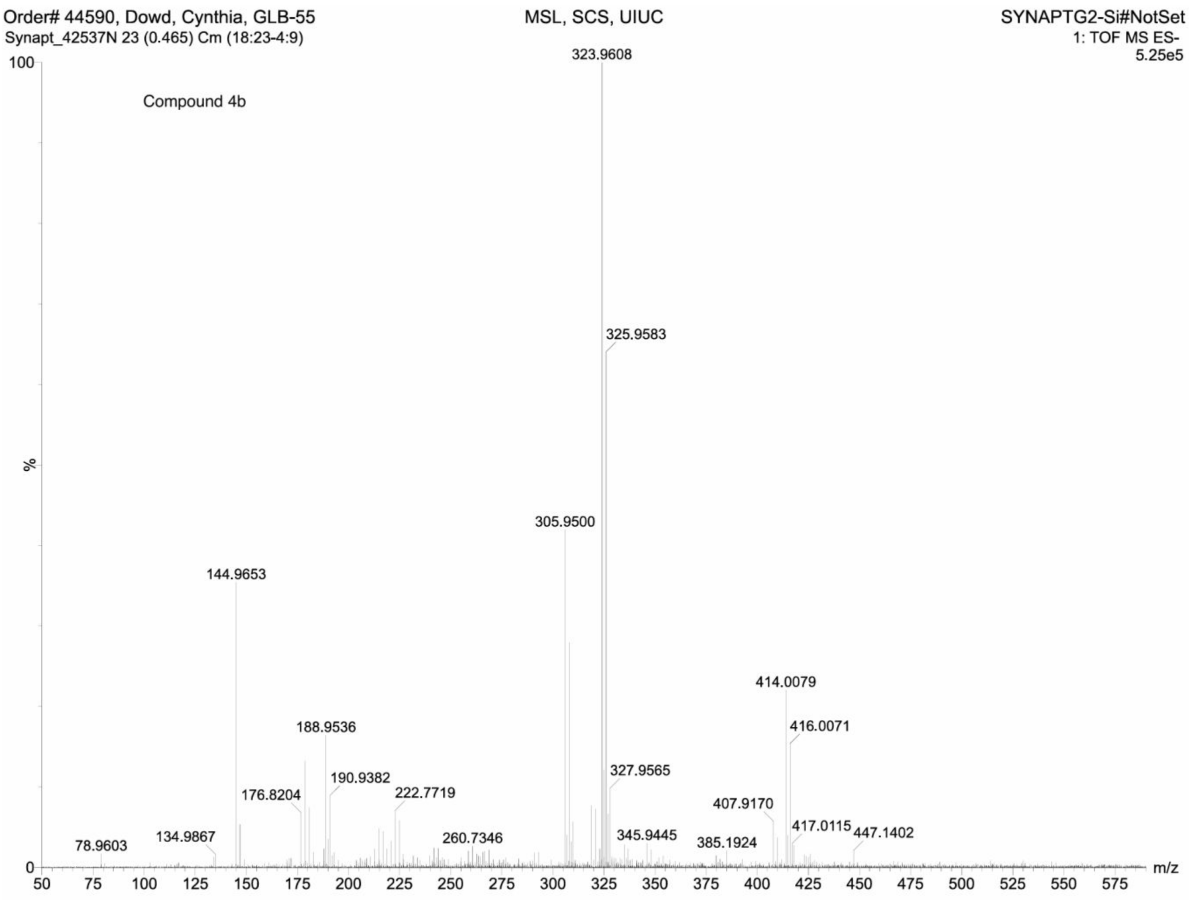

Sodium hydrogen [(1E)–3–[1–(4–methylphenyl)–N–hydroxyformamido] prop–1–en–1–yl] phosphonate (5b). ^{1}H NMR (400 MHz, DMSO) δ 7.77 (d, J = 21.2 Hz, 1H), 7.58 (s, 1H), 7.13 (s, 2H), 6.47–6.09 (m, 1H), 6.09–5.76 (m, 1H), 4.57–3.86 (m, 2H), 2.28 (s, 3H). ^{13}C NMR (101 MHz, DMSO) δ 168.56, 139.71, 132.00, 129.41, 128.72, 128.41, 128.20, 51.98, 21.06. HRMS (ESI^{+}) calculated for $C_{10}H_{13}NO_5P$: 270.0531; found 270.0544 [M-H]$^{-}$.

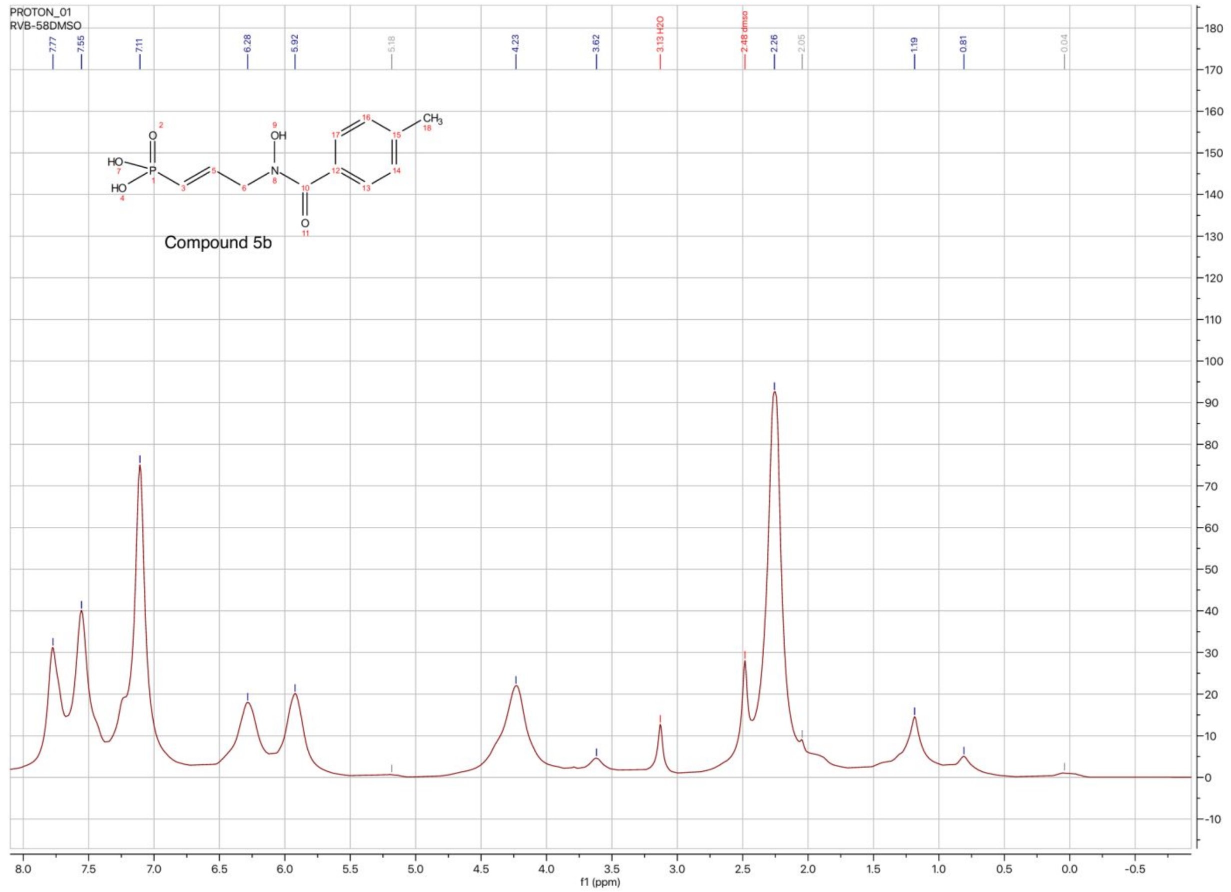
PROTON_01
RVB-58DMSO
7.77
7.55
7.11
6.28
5.92
5.18
4.23
3.62
3.13 H2O
2.48 dmso
2.26
2.05
1.19
0.81
0.04
Compound 5b
f1 (ppm)

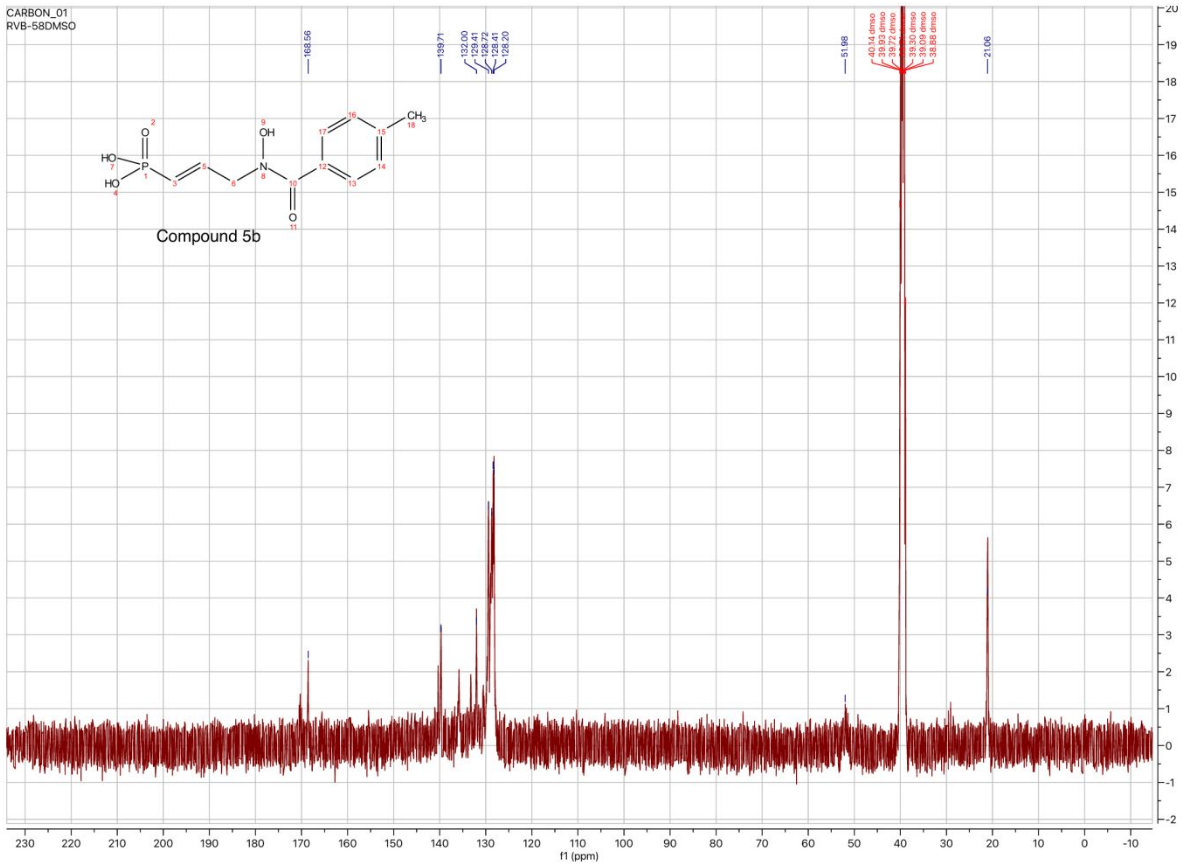

CARBON_01
RVB-58DMSO
168.56
139.71
132.00
129.41
128.72
128.41
128.20
51.98
40.14 dmso
39.93 dmso
39.72 dmso
39.30 dmso
39.09 dmso
38.88 dmso
21.06
Compound 5b
f1 (ppm)

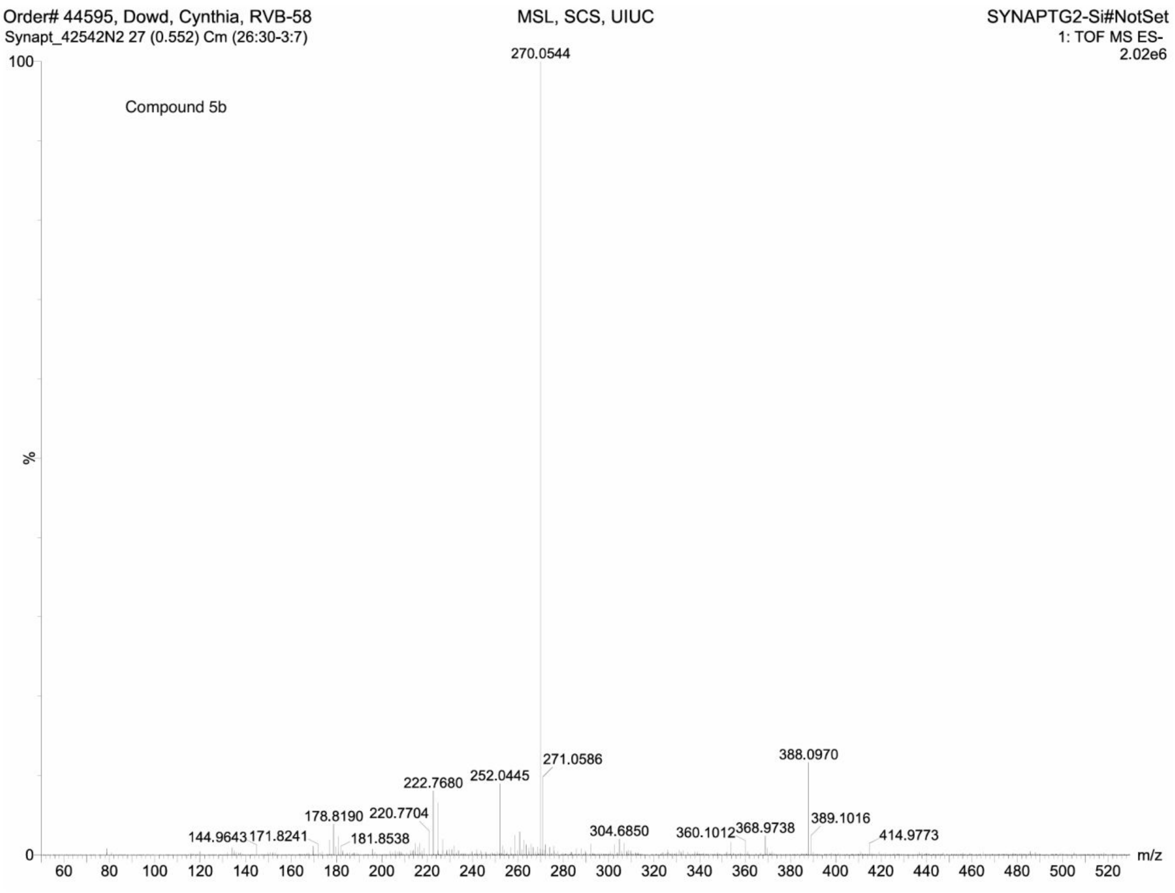

Sodium hydrogen [(1E)–3–[1–(4–tert–butylphenyl)–N–hydroxyformamido] prop–1–en–1–yl] phosphonate (6b). ^{1}H NMR (400 MHz, CD_3OD) δ 7.88 – 7.85 (m, 1H), 7.41 – 7.35 (m, 3H), 6.52 – 6.25 (m, 1H), 6.21 – 5.87 (m, 1H), 4.56 – 3.99 (m, 2H), 1.31 (d, J = 4.3 Hz, 9H). ^{13}C NMR (151 MHz, $CDCl_3$) δ 144.70, 133.51, 129.84, 128.61, 125.24, 124.84, 62.07, 40.89, 16.35. LCMS (ESI$^-$): 331 *m/z* $[M+H_2O]^-$. HRMS (ESI$^+$) calculated for $C_{14}H_{19}NO_5P$: 312.1001; found 312.1005 $[M-H]^-$.

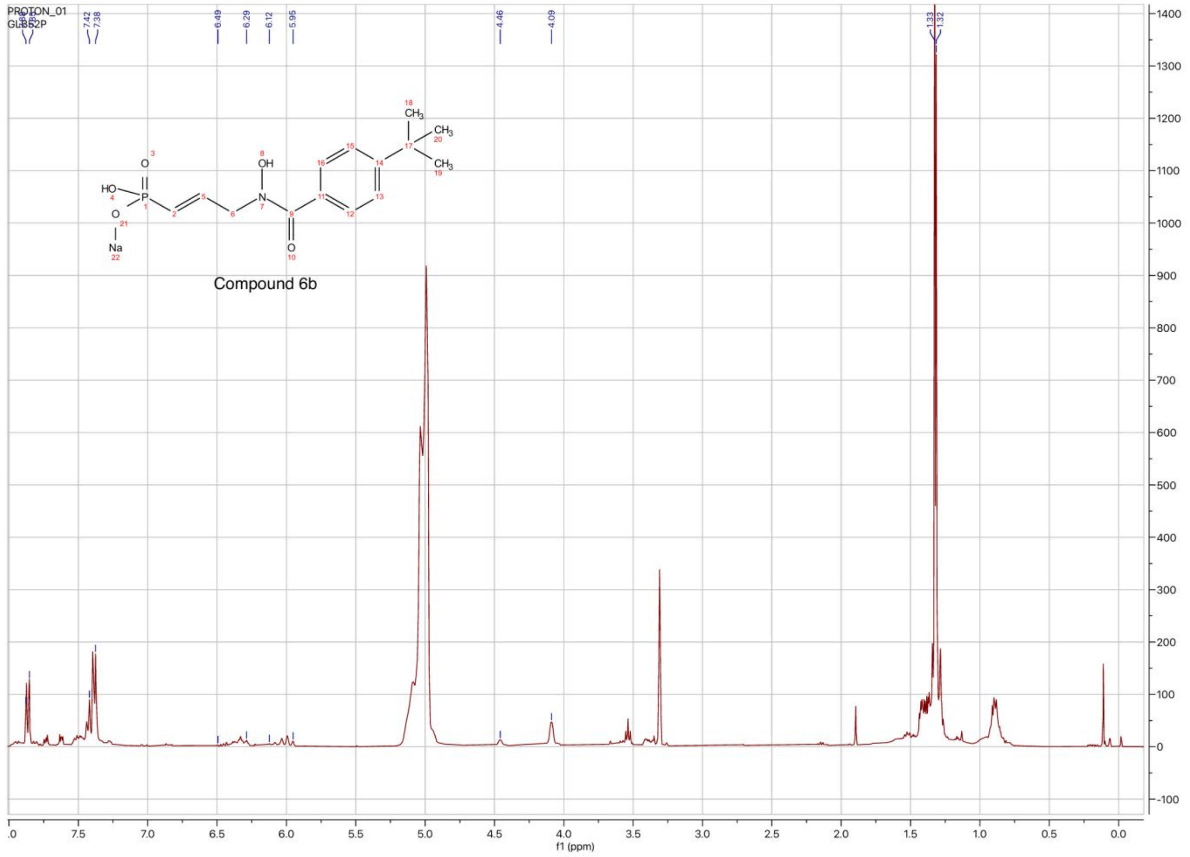

PROTON_01
GLB62P
7.42
7.38
6.49
6.29
6.12
5.95
4.46
4.09
1.33
1.32
Compound 6b
f1 (ppm)

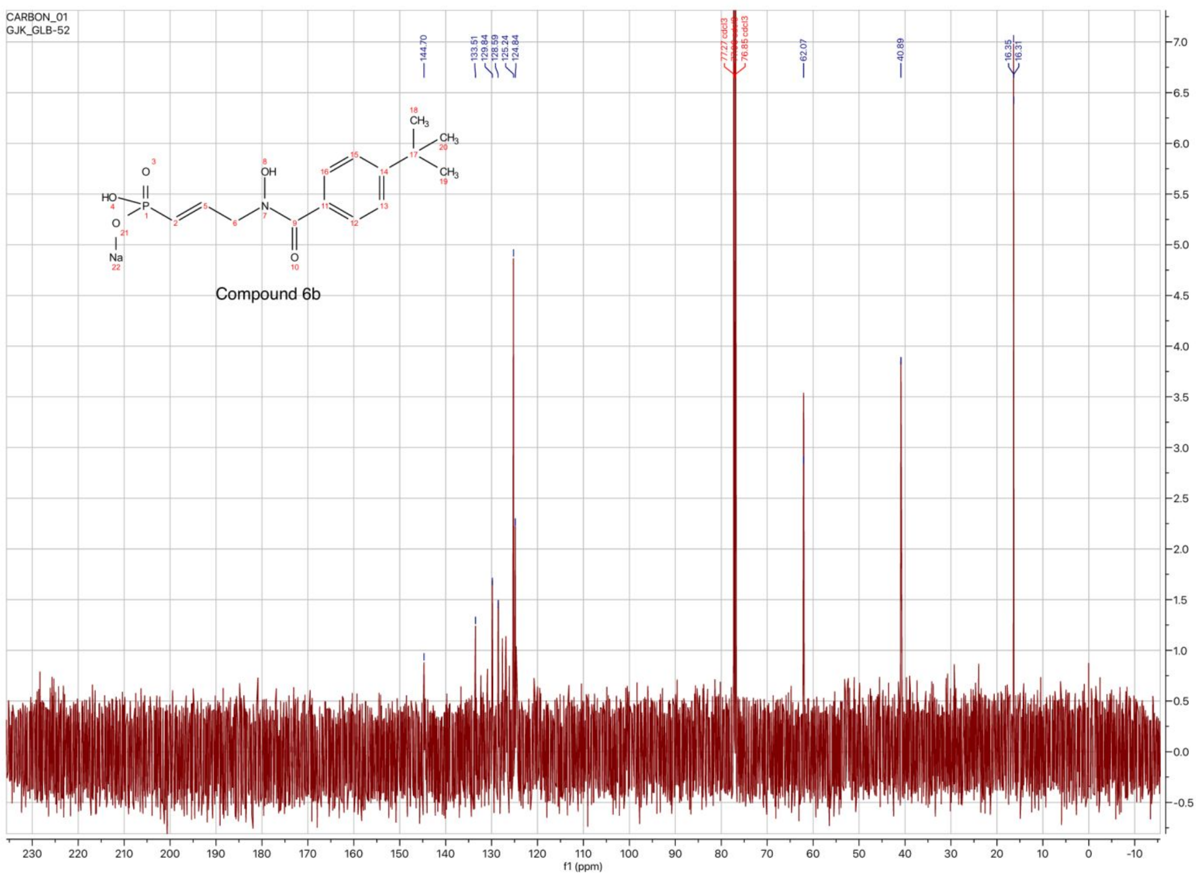
CARBON_01
GJK_GLB-52
144.70
133.51
129.84
128.59
125.24
124.84
77.27 cdcl3
76.85 cdcl3
62.07
40.89
16.35
16.31
Compound 6b
f1 (ppm)

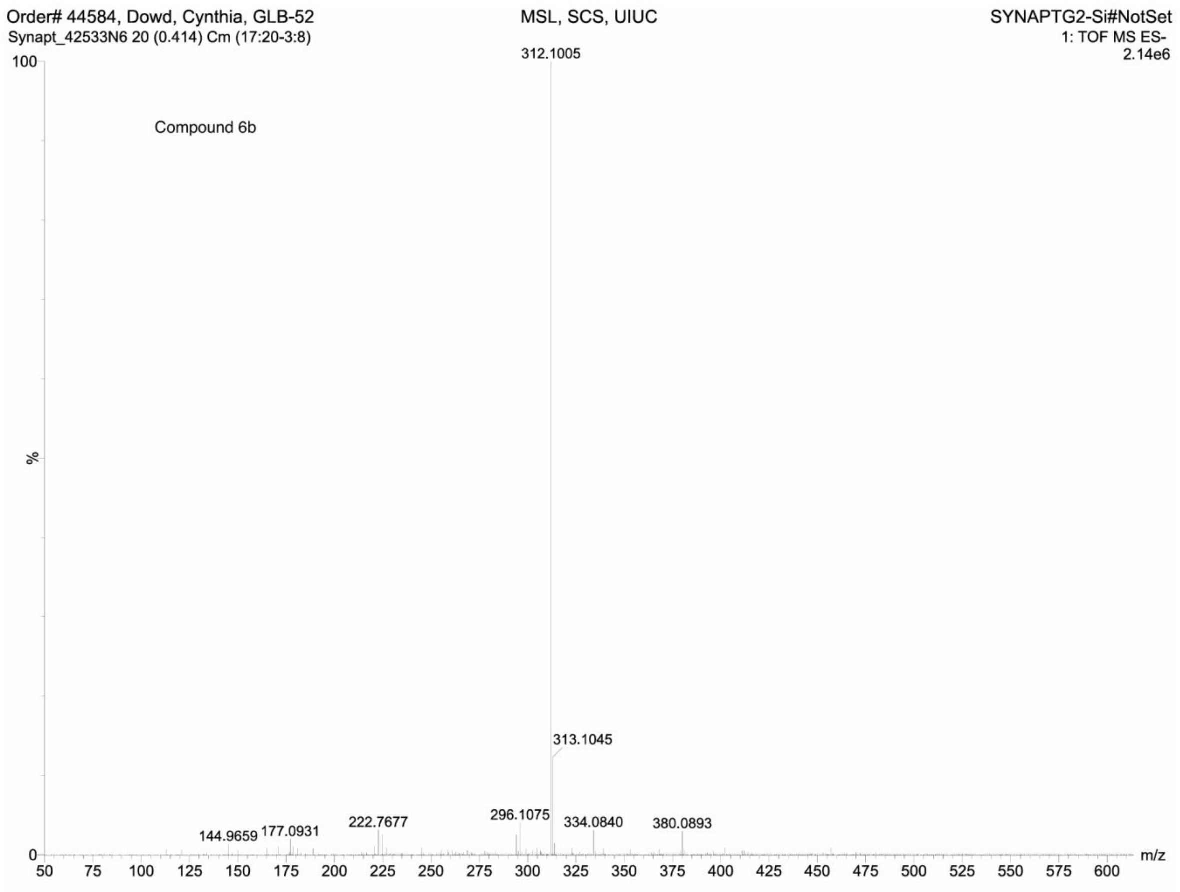

Sodium hydrogen [(1E)-3-[1-(4-fluorophenyl)-N-hydroxyformamido]prop-1-en-1-yl]phosphonate (7b). ^{1}H NMR (400 MHz, CD_3OD) δ 7.93–7.81 (m, 1H), 7.66–7.55 (m, 1H), 7.27–7.20 (m, 2H), 6.53–6.36 (m, 1H), 6.18–5.99 (m, 1H), 4.36 (d, J = 5.6 Hz, 2H), 2.53–2.43 (m, 3H).^{13}C NMR (101 MHz, CD_3OD) δ 172.31, 171.13, 137.98, 131.07, 130.60, 128.66, 125.97, 55.56. LCMS (ESI$^-$): 274 *m/z* [MH]$^-$. HRMS (ESI$^+$) calculated for $C_{10}H_{10}FNO_5P$: 274.0281; found 274.0293 [M-H]$^-$.

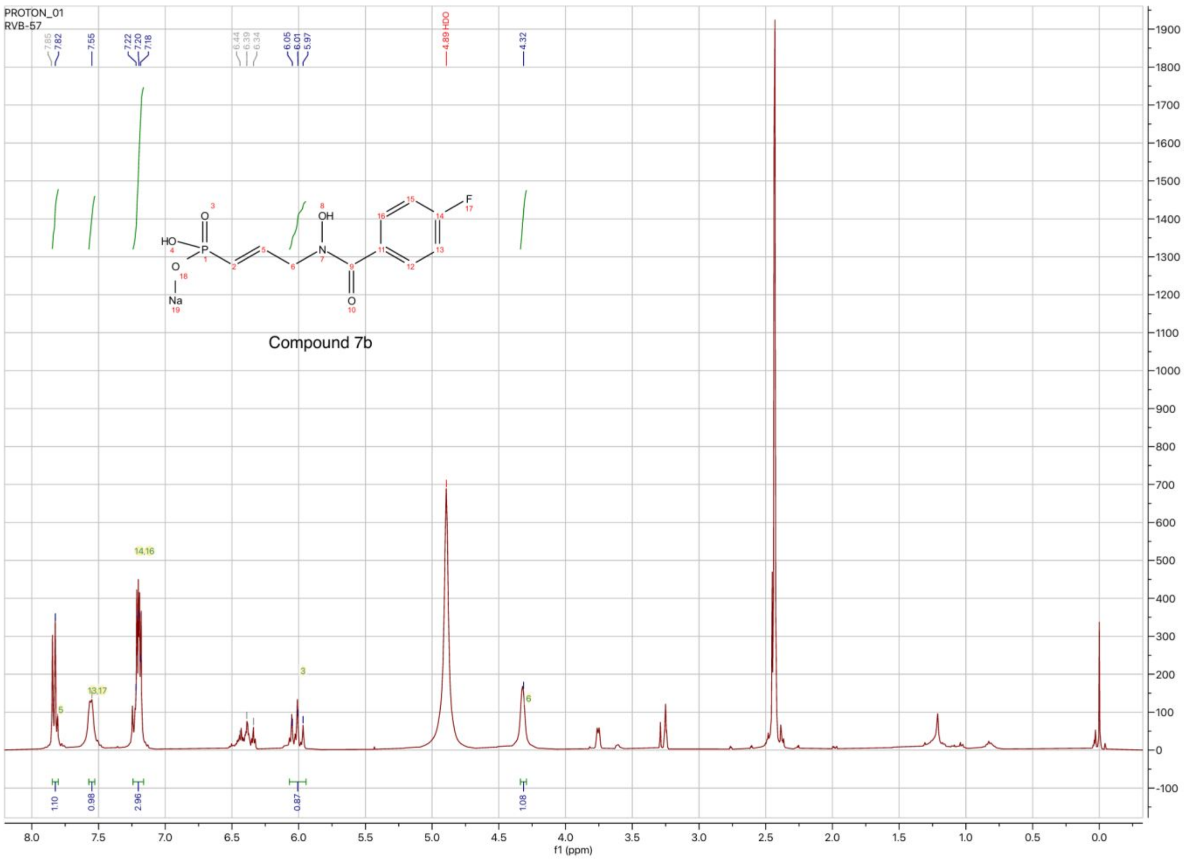
PROTON_01
RVB-57
Compound 7b
f1 (ppm)

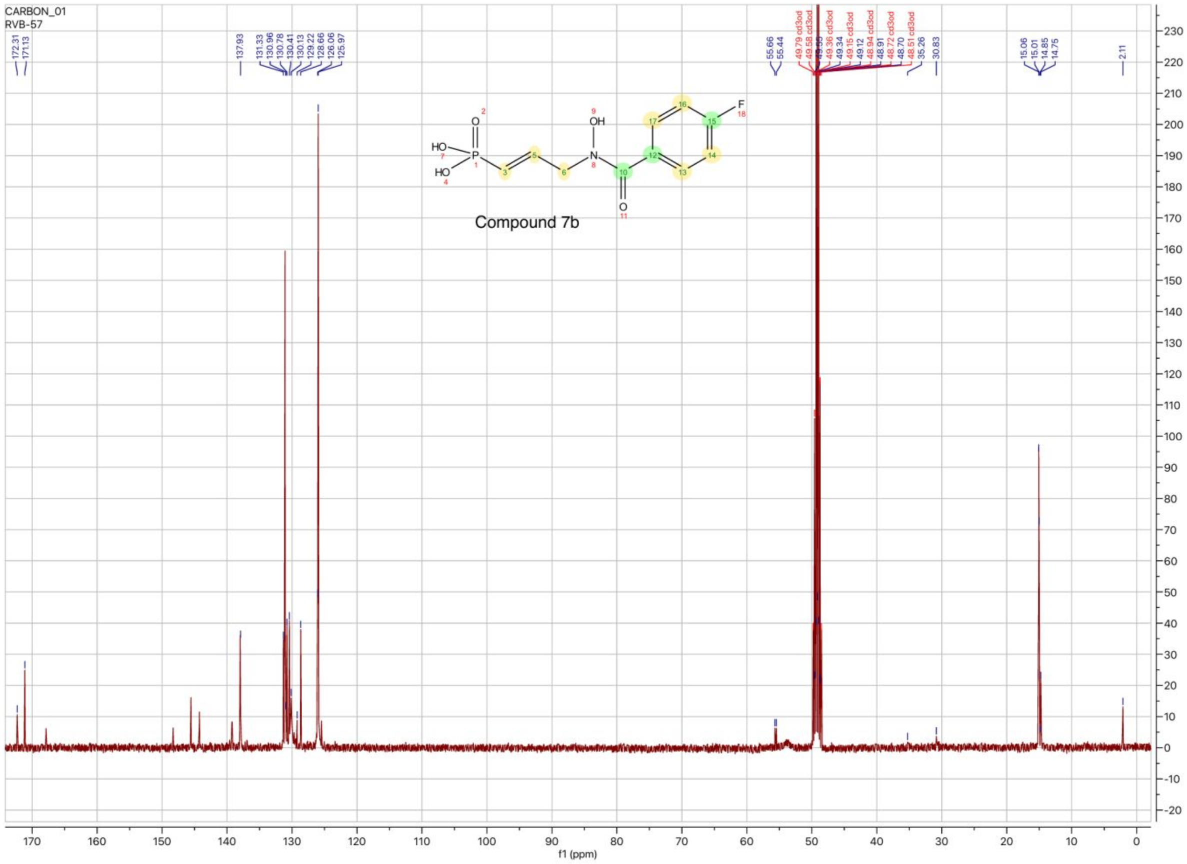

CARBON_01
RVB-57
Compound 7b
f1 (ppm)

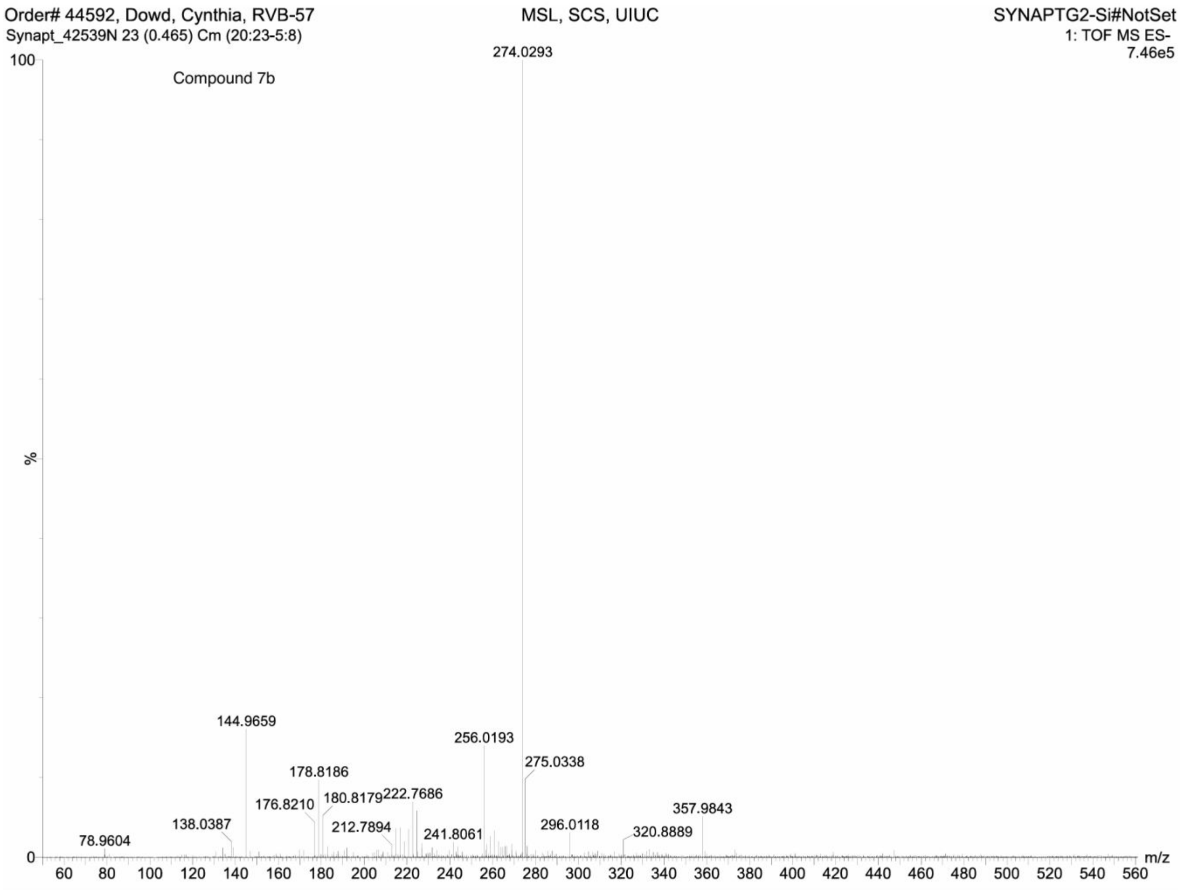

Sodium hydrogen [(1E)-3-{N-hydroxy-1-[3-(trifluoromethyl)phenyl] formamido}prop-1-en-1-yl]phosphonate (8b). ^{1}H NMR (400 MHz, CD_3OD) δ 8.19 (d, J = 17.7 Hz, 1H), 7.91 (d, J = 15.8 Hz, 1H), 7.81 – 7.49 (m, 2H), 6.58 – 6.23 (m, 1H), 6.02 (q, J = 16.5 Hz, 1H), 4.37 (s, 2H). LCMS (ESI$^-$): 324 *m/z* [M-H]$^-$. ^{13}C NMR (101 MHz, DMSO) δ 166.87, 136.03, 135.62, 133.01, 132.71, 128.96, 128.68, 126.52, 125.46, 125.15, 48.65. HRMS (ESI$^+$) calculated for $C_{11}H_{10}F_3NO_5P$: 324.0249; found 324.0255 [M-H]$^-$.

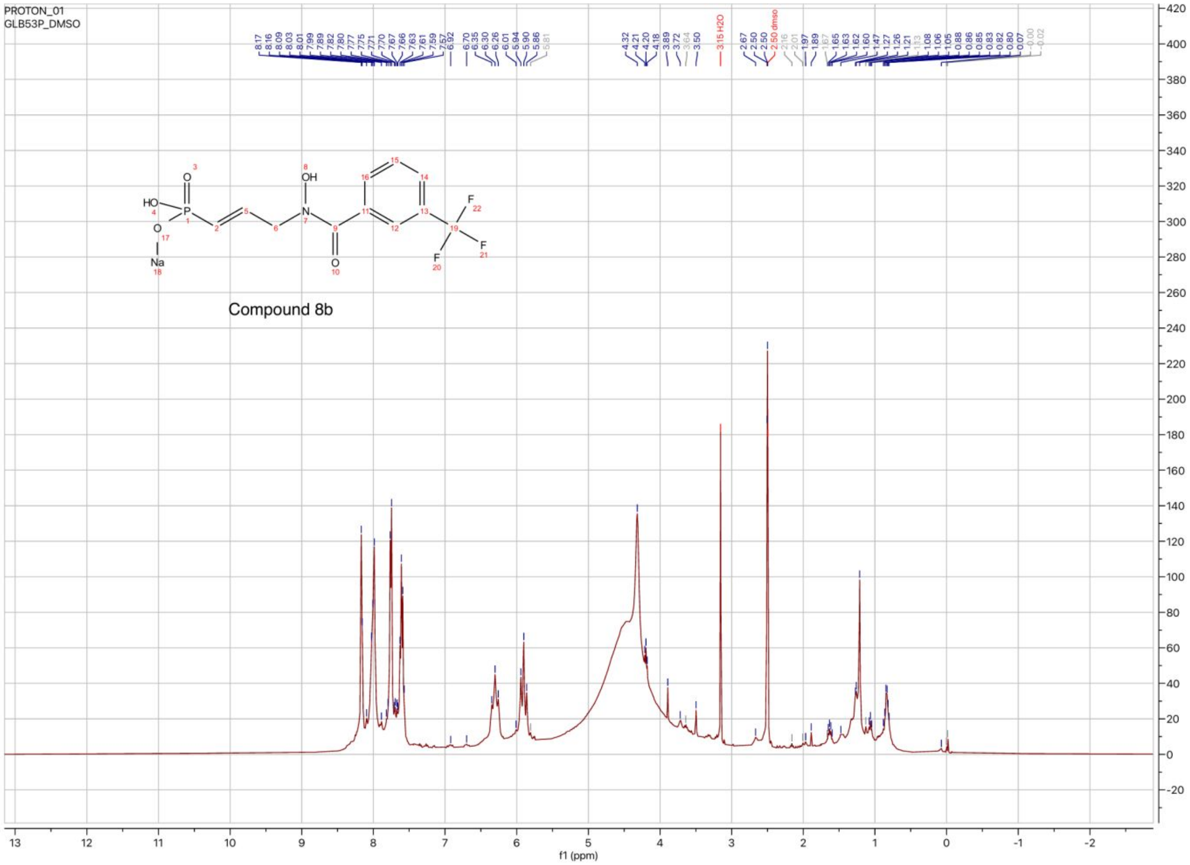
PROTON_01
GLB53P_DMSO
Compound 8b
f1 (ppm)

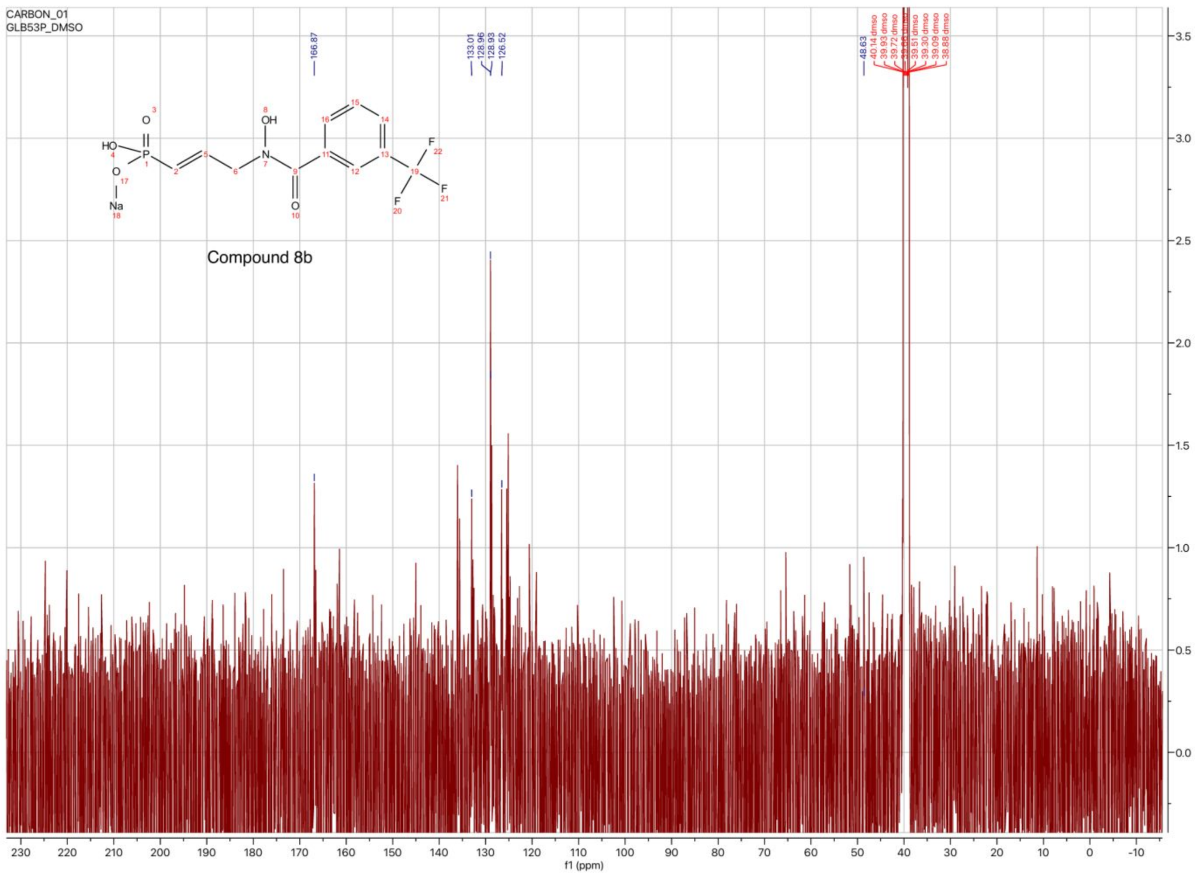

CARBON_01
GLB53P_DMSO
166.87
133.01
128.96
128.93
126.52
48.63
40.14 dmso
39.93 dmso
39.72 dmso
39.51 dmso
39.30 dmso
39.09 dmso
38.88 dmso
Compound 8b
f1 (ppm)

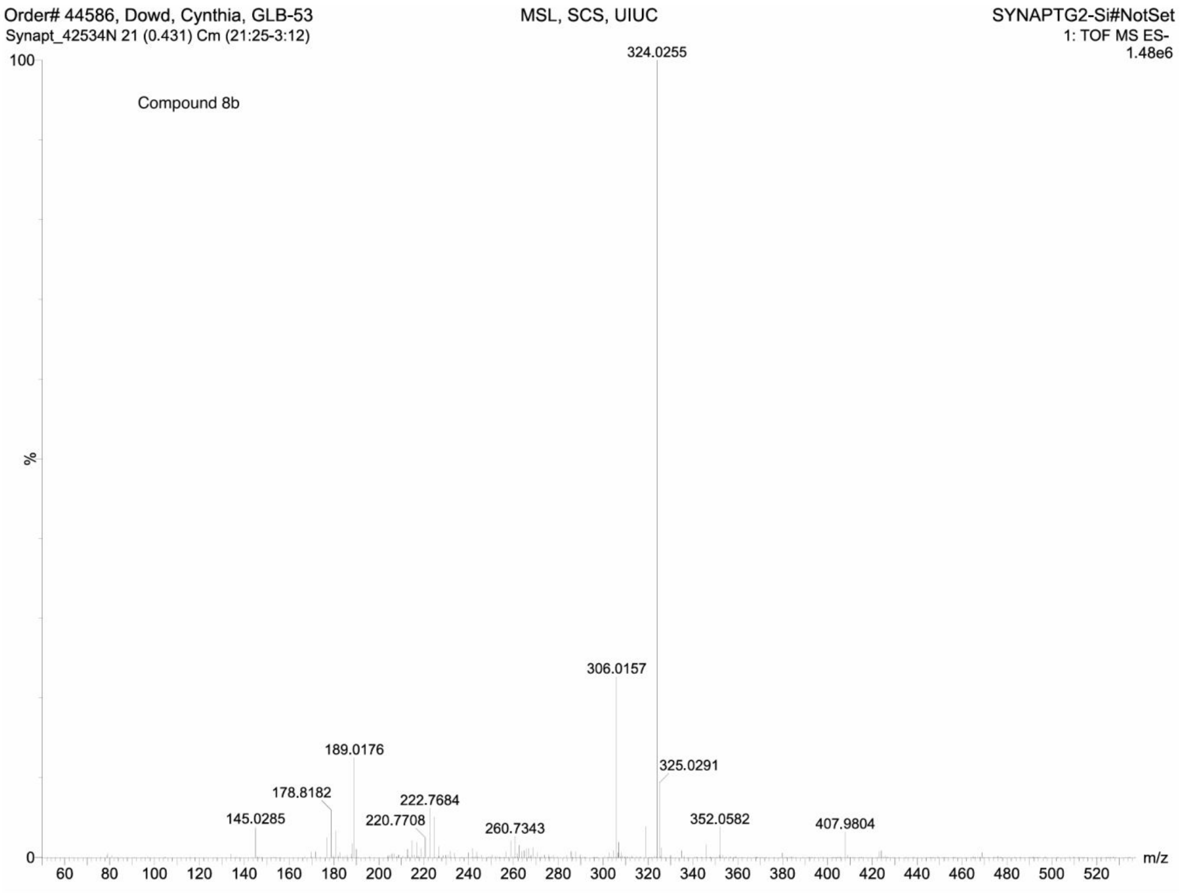

Sodium hydrogen [(1E)-3-{N-hydroxy-1-[4-(trifluoromethyl)phenyl] formamido}prop-1-en-1-yl]phosphonate (9b). ^{1}H NMR (400 MHz, CD_3OD) δ 8.18–7.75 (m, 2H), 7.69 (d, J = 30.7 Hz, 2H), 6.49 (q, J = 22.2, 18.8 Hz, 1H), 6.07 (t, J = 17.3 Hz, 1H), 4.44 (s, 2H). LCMS (ESI-): 324 m/z $[M-H]^-$. ^{13}C NMR (101 MHz, CD_3OD) δ 170.10, 139.51, 138.04, 131.22, 130.19, 128.36, 126.67, 125.84, 52.80. HRMS (ESI^+) calculated for $C_{11}H_{10}F_3NO_5P$: 324.0249; found 324.0262 $[M-H]^-$.

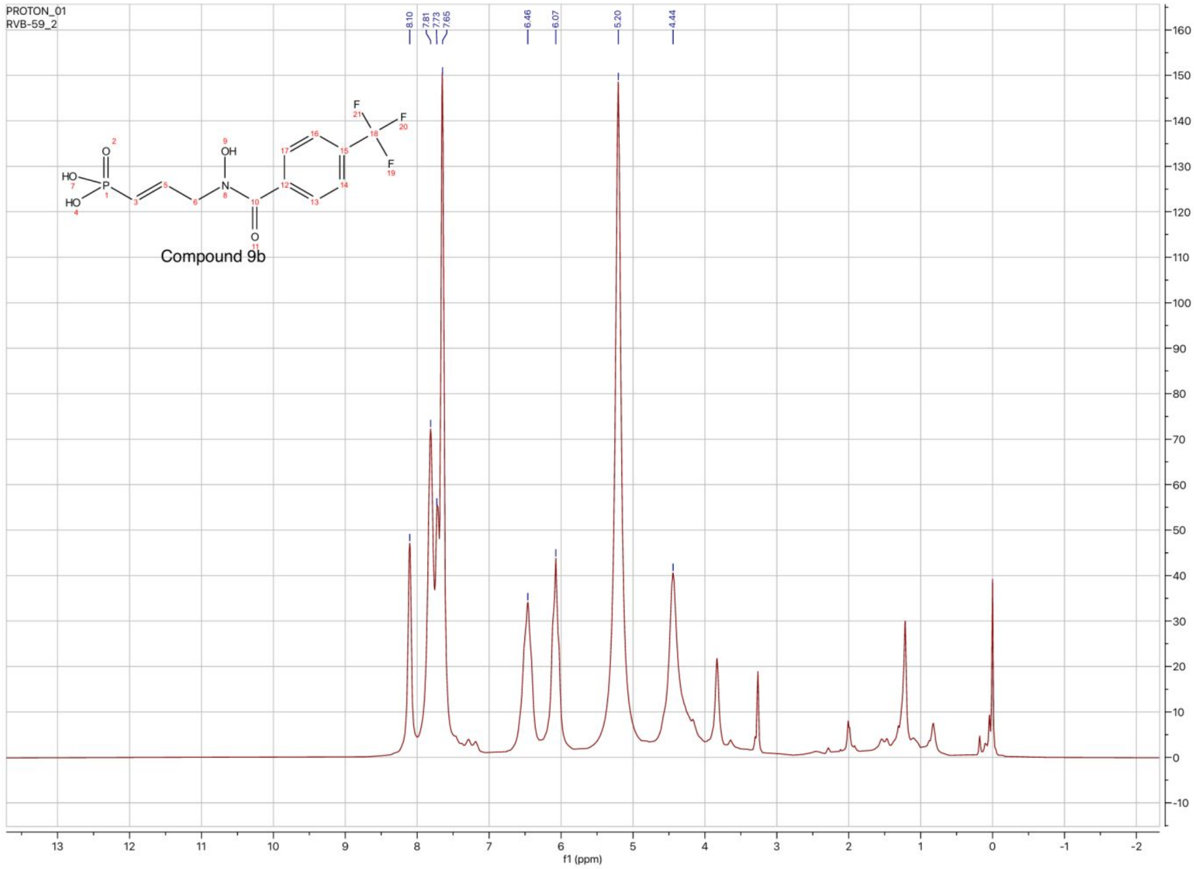

PROTON_01
RVB-59_2
8.10
7.81
7.73
7.65
6.46
6.07
5.20
4.44
Compound 9b
f1 (ppm)

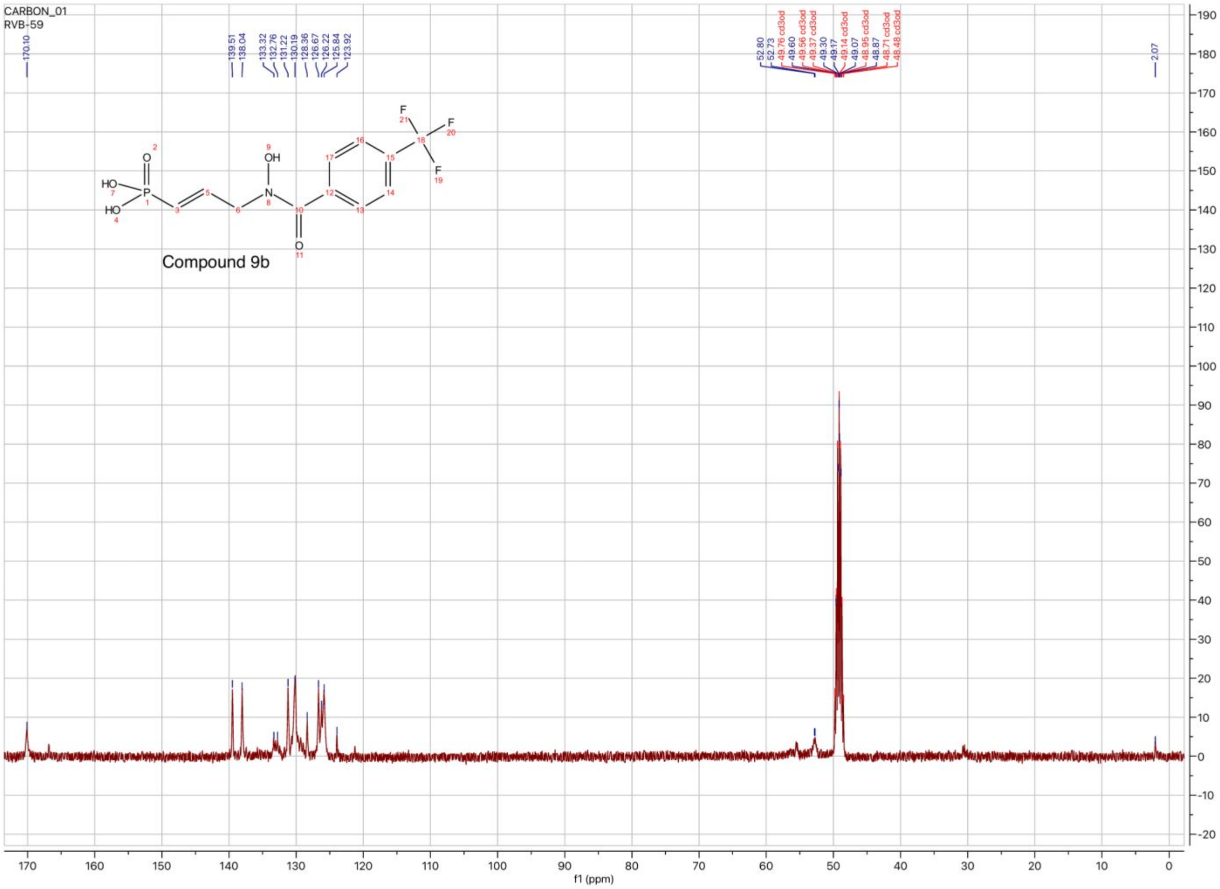
CARBON_01
RVB-59
Compound 9b
f1 (ppm)

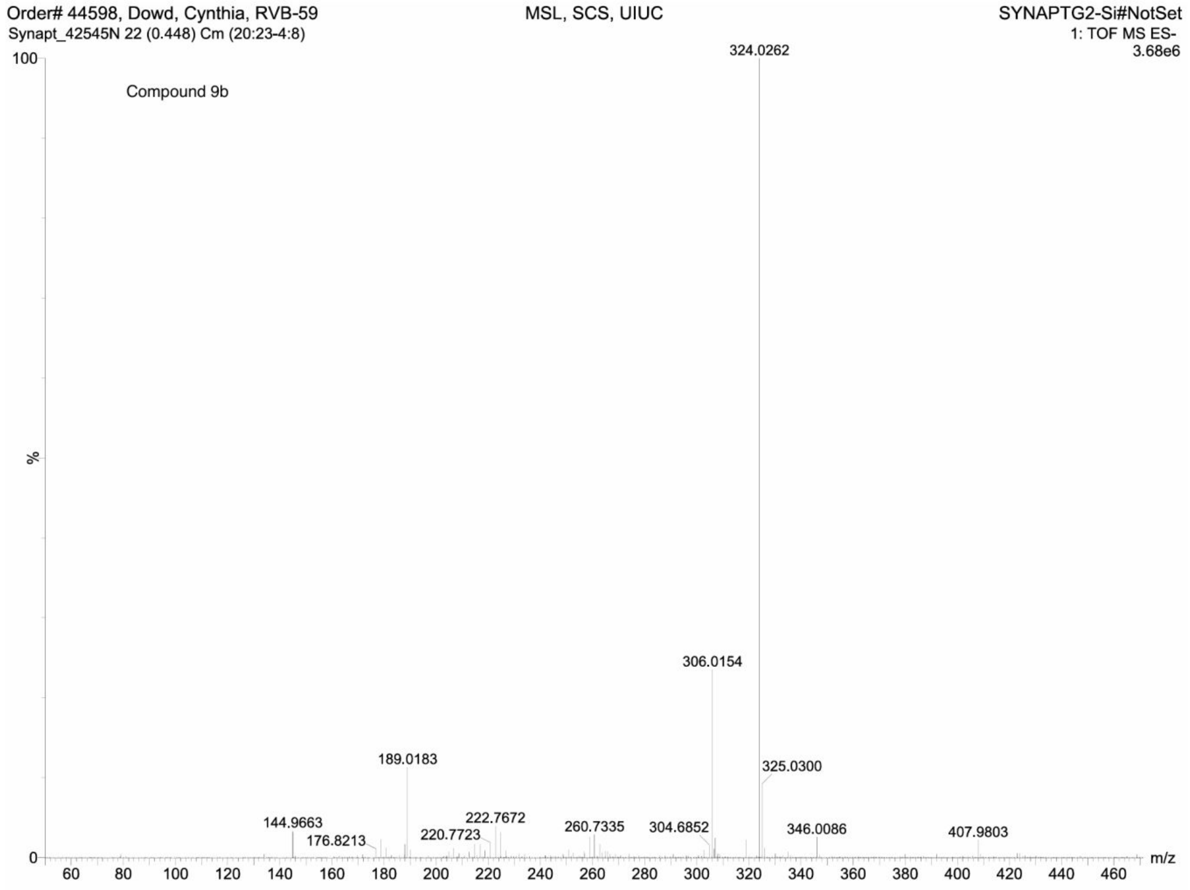

Sodium hydrogen [(1E)–3–[N–hydroxy–1–(naphthalen–1–yl)formamido]prop–1–en–1–yl] phosphonate (10b). ^{1}H NMR (400 MHz, CD_3OD) δ 7.95 – 7.66 (m, 3H), 7.56 – 7.26 (m, 4H), 6.29 – 6.03 (m, 1H), 6.03 – 5.70 (m, 1H), 3.83 (s, 2H). ^{13}C NMR (101 MHz, CD_3OD) δ 166.64, 135.46, 134.96, 134.71, 133.84, 131.22, 130.51, 129.50, 128.25, 127.66, 126.37, 126.17, 125.92, 57.40. LCMS (ESI$^-$): 306 *m/z* [M-H]$^-$. HRMS (ESI$^+$) calculated for $C_{14}H_{13}NO_5P$: 306.0531; found 306.0545 [M-H]$^-$.

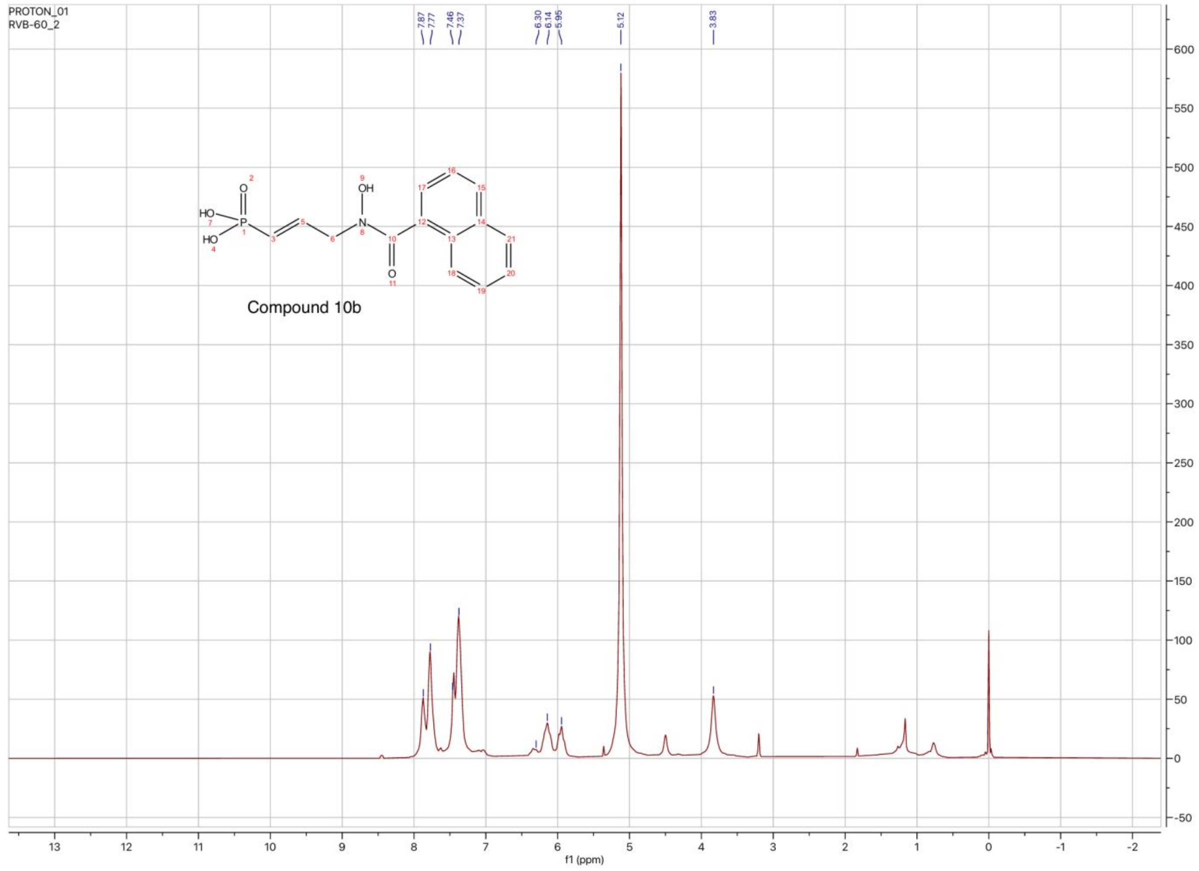
PROTON_01
RVB-60_2
7.87
7.77
7.46
7.37
6.30
6.14
5.96
5.12
3.83
Compound 10b
f1 (ppm)

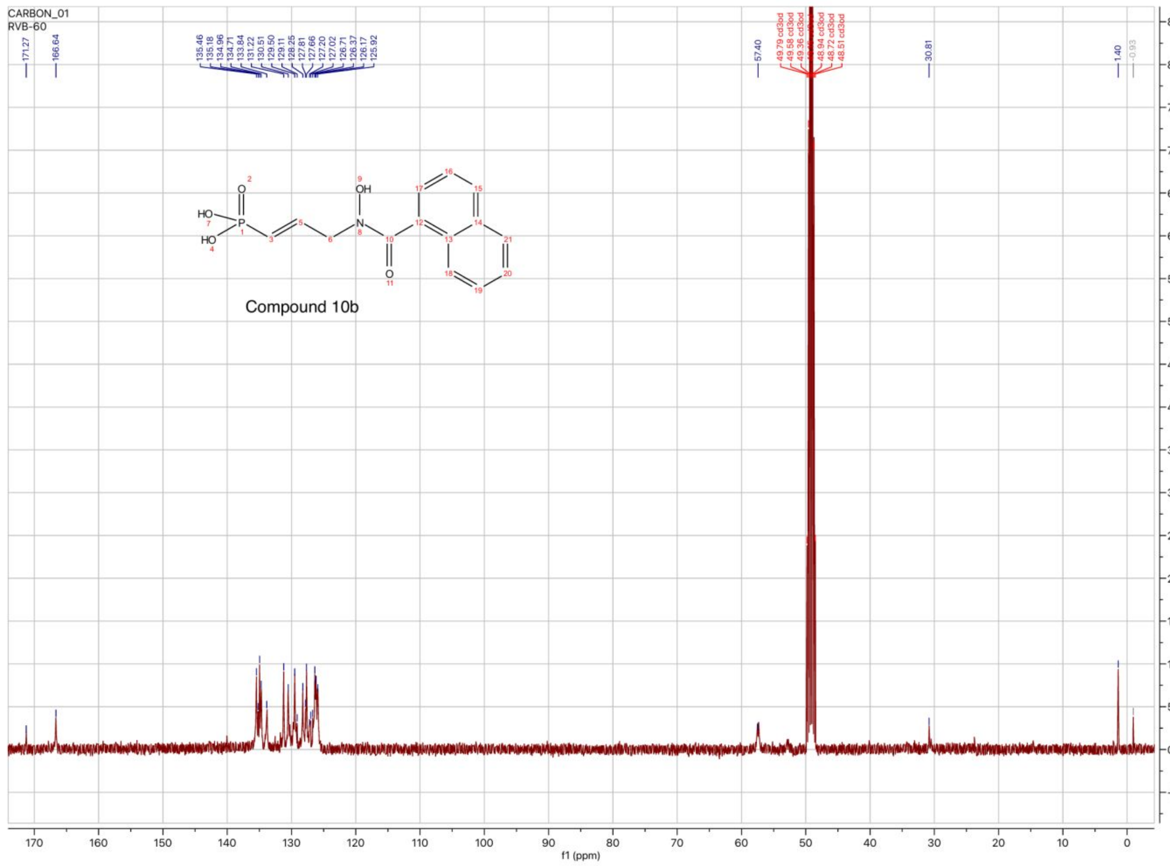
CARBON_01
RVB-60
171.27
166.64
135.46
135.18
134.96
134.71
133.84
131.22
130.51
129.50
129.11
128.25
127.81
127.66
127.20
127.02
126.71
126.37
126.17
125.92
57.40
49.79 cd3od
49.58 cd3od
49.36 cd3od
48.94 cd3od
48.72 cd3od
48.51 cd3od
30.81
1.40
-0.93
Compound 10b
f1 (ppm)

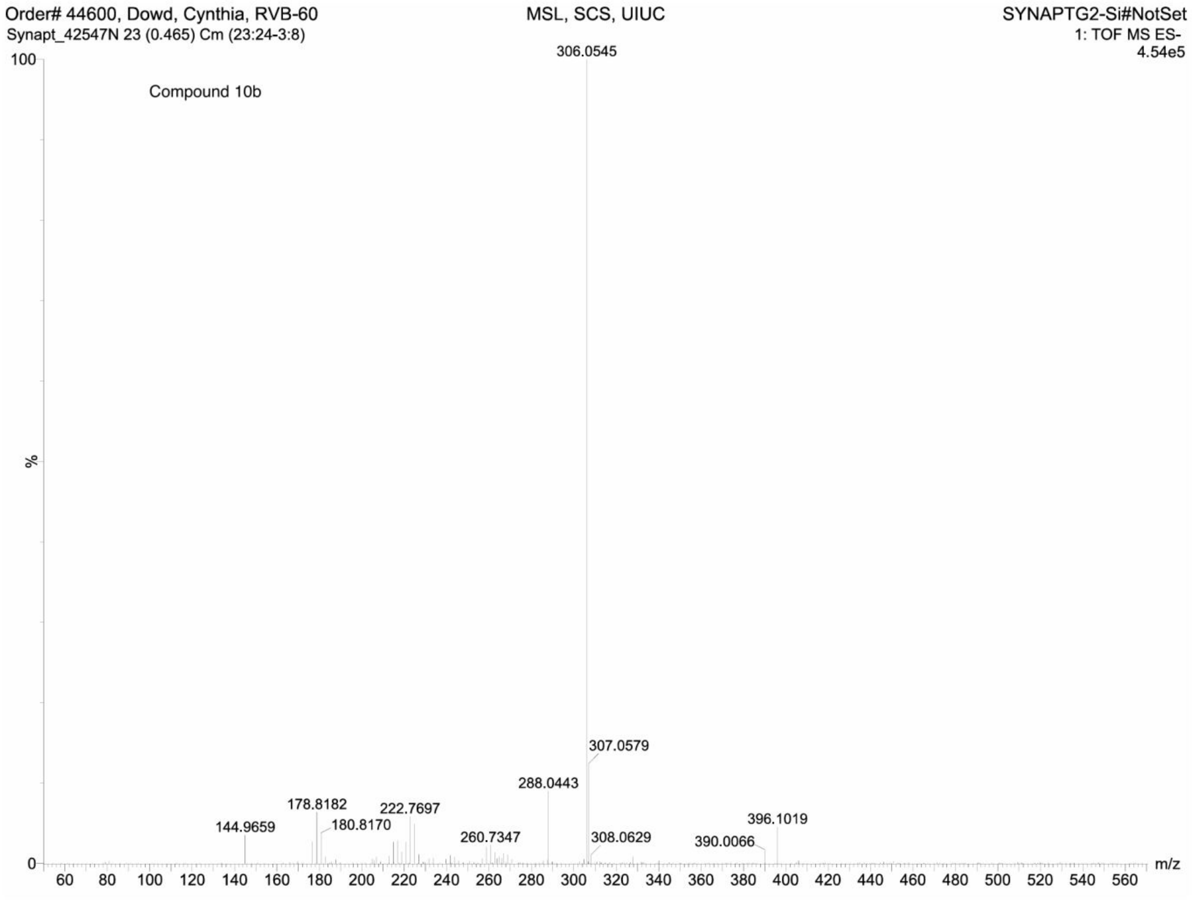

Sodium hydrogen [(1E)–3–[N–hydroxy–1–(naphthalen–2–yl)formamido]prop–1–en–1–yl]phosphonate (11b). ^{1}H NMR (400 MHz, CD_3OD) δ 8.59 (d, J = 1.7 Hz, 1H), 8.03 – 7.96 (m, 2H), 7.91 (dd, J = 10.7, 6.0 Hz, 3H), 7.62 – 7.53 (m, 2H), 6.75 – 6.41 (m, 1H), 6.34 – 5.99 (m, 1H), 4.79 – 4.33 (m, 2H). ^{13}C NMR (101 MHz, CD_3OD) δ 170.22, 137.10, 134.07, 133.94, 132.24, 130.43, 129.86, 129.51, 129.31, 129.30, 128.92, 127.94, 126.45. LCMS (ESI$^-$): 306 *m/z* [M-H]$^-$. HRMS (ESI$^+$) calculated for $C_{14}H_{13}NO_5P$: 306.0531; found 306.0537 [M-H]$^-$.

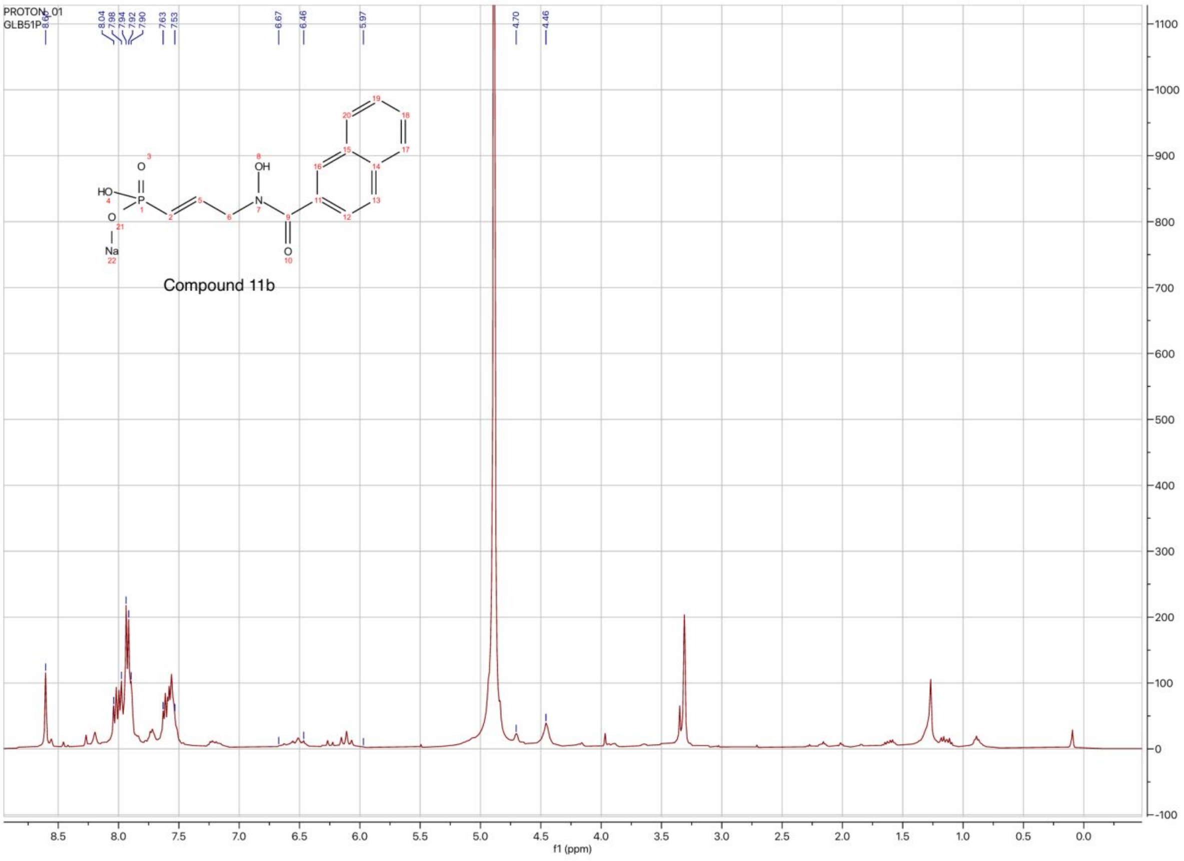
PROTON_01
GLB51P
8.60
8.04
7.98
7.94
7.92
7.90
7.63
7.53
6.67
6.46
5.97
4.70
4.46
Compound 11b
f1 (ppm)

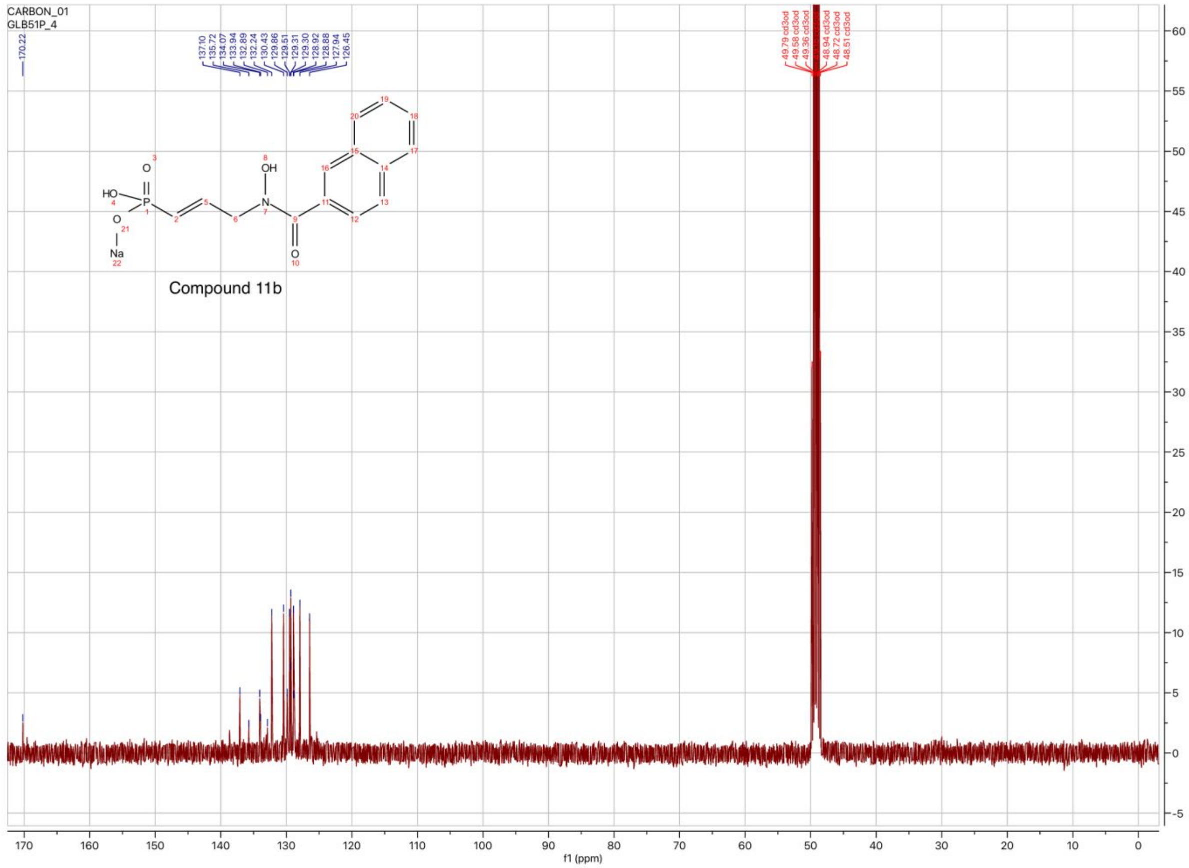

CARBON_01
GLB51P_4
170.22
137.10
135.72
134.07
133.94
132.89
132.24
130.43
129.86
129.51
129.31
129.30
128.92
128.88
127.94
126.45
49.79 cd3od
49.58 cd3od
49.36 cd3od
48.94 cd3od
48.72 cd3od
48.51 cd3od
Compound 11b
f1 (ppm)

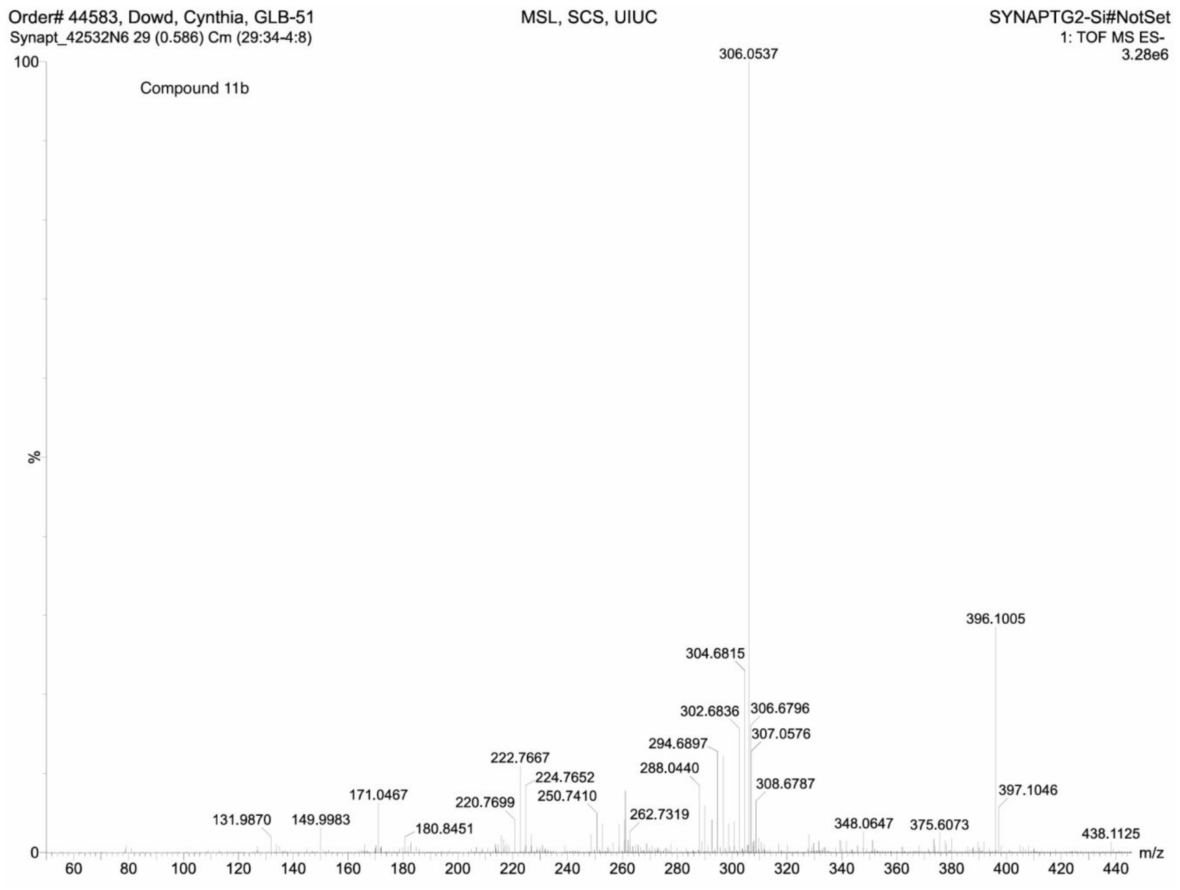

Sodium hydrogen [(1E)–3–(1–{[1,1'–biphenyl]–4–yl}–N–hydroxyformamido) prop–1–en–1–yl] phosphonate (12b). 1H NMR (400 MHz, DMSO) δ 8.02 (s, 1H), 7.79 (s, 2H), 7.68 (s, 4H), 7.51 – 7.38 (m, 3H), 6.36 (s, 1H), 5.96 (s, 1H), 4.34 (s, 2H). ^{13}C NMR (101 MHz, CD_3OD) δ 171.38, 144.79, 138.04, 134.33, 131.73, 131.45, 130.13, 129.11, 128.37, 128.19, 127.68. LCMS (ESI^-): 332 *m/z* $[M-H]^-$. HRMS (ESI^+) calculated for $C_{16}H_{15}NO_5P$: 332.0688; found 332.0689 $[M-H]^-$.

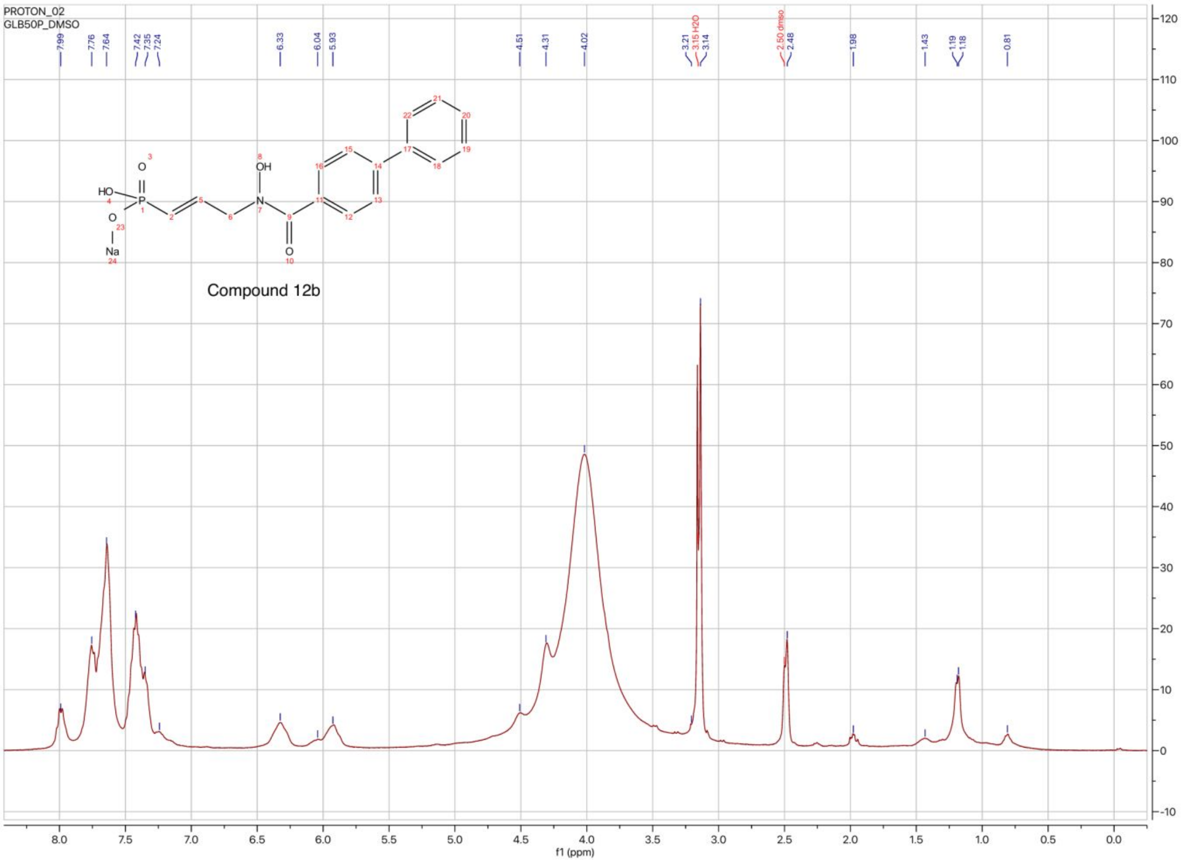
PROTON_02
GLB50P_DMSO
7.99
7.76
7.64
7.42
7.35
7.24
6.33
6.04
5.93
4.51
4.31
4.02
3.21
3.15 H2O
3.14
2.50 dmso
2.48
1.98
1.43
1.19
1.18
0.81
Compound 12b
f1 (ppm)

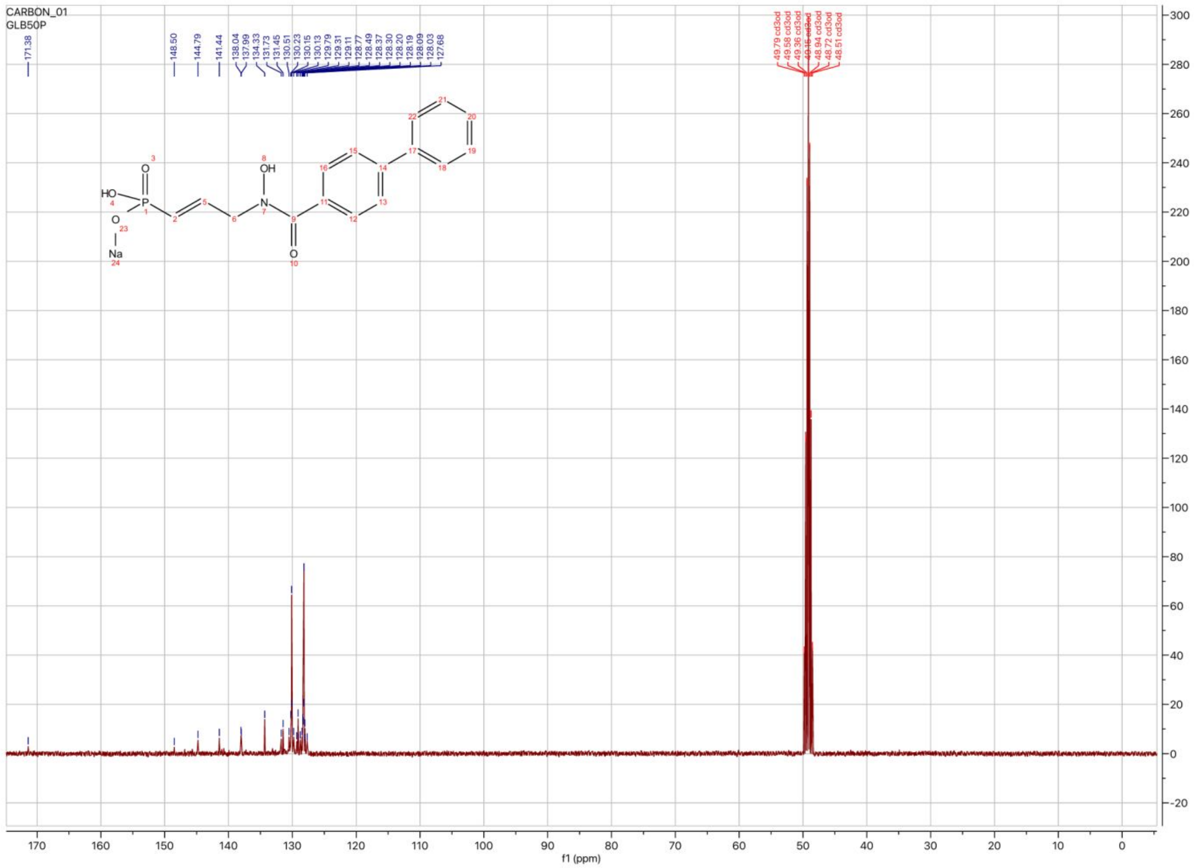

CARBON_01
GLB50P
171.38
148.50
144.79
141.44
138.04
137.99
134.33
131.73
131.45
130.51
130.23
130.15
130.13
129.79
129.31
129.11
128.77
128.49
128.37
128.30
128.20
128.19
128.09
128.03
127.68
49.79 cd3od
49.58 cd3od
49.36 cd3od
49.15 cd3od
48.94 cd3od
48.72 cd3od
48.51 cd3od
HO
O
Na
OH
N
O
f1 (ppm)

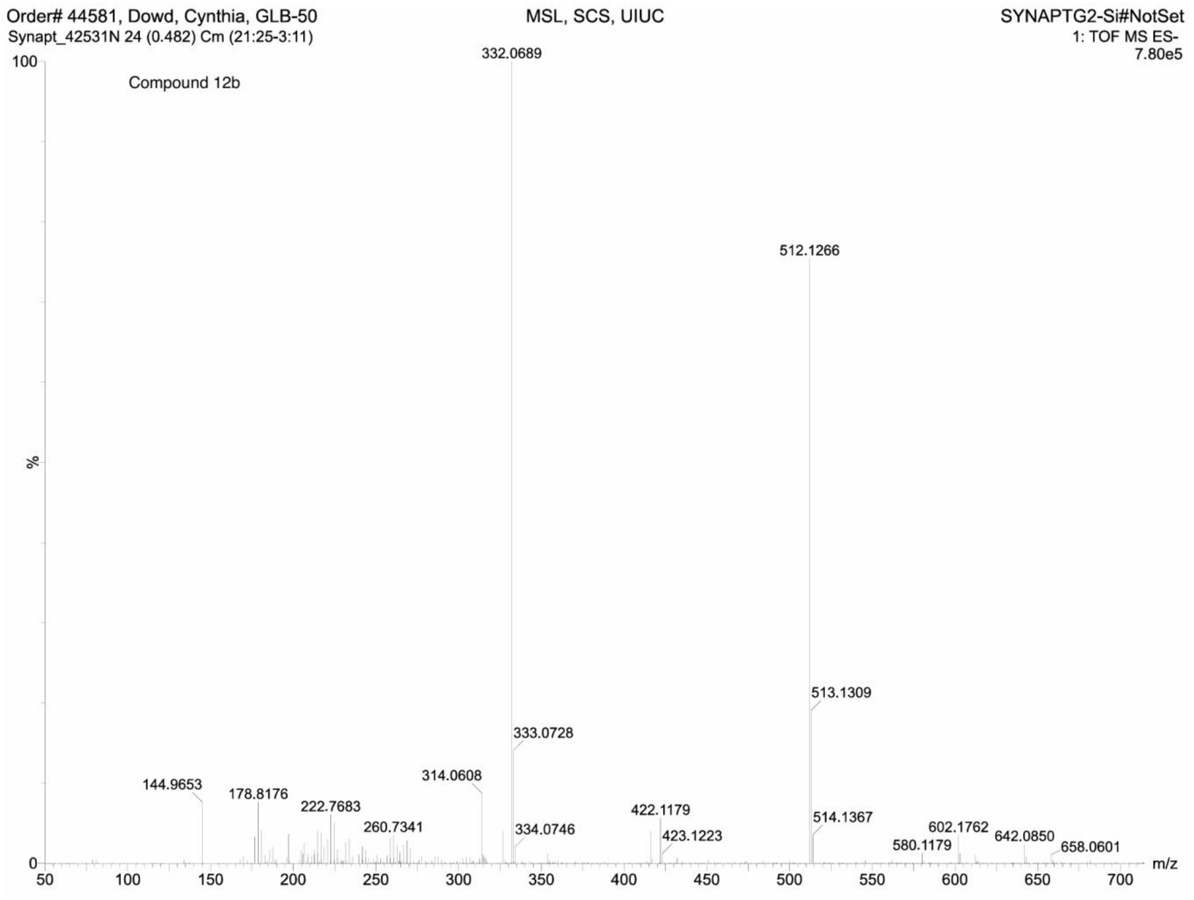

Sodium hydrogen [(1E)–3–[N–hydroxy–1–(pyridin–4–yl)formamido]prop–1–en–1–yl] phosphonate (13b). ^{1}H NMR (400 MHz, DMSO) δ 8.85 – 8.39 (m, 2H), 7.66 (s, 2H), 6.48 – 6.31 (m, 1H), 5.91 (t, J = 17.7 Hz, 1H), 4.38 (s, 2H). ^{13}C NMR (101 MHz, DMSO) δ 170.46, 150.41, 148.62, 140.10, 123.48, 122.97, 62.95. HRMS (ESI^{+}) calculated for $C_9H_{10}N_2O_5P$: 257.0327; found 257.0332 [M-H]$^{-}$.

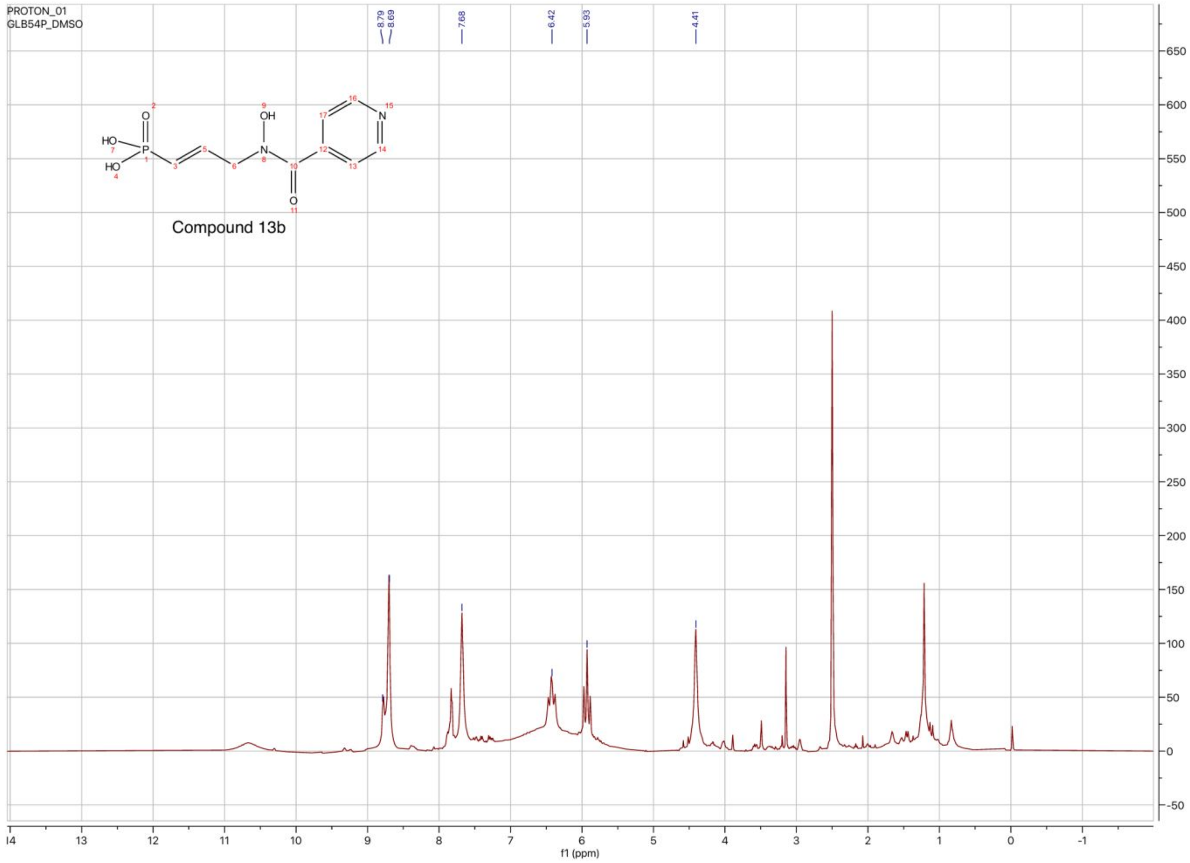

PROTON_01
GLB54P_DMSO
8.79
8.69
7.68
6.42
5.93
4.41
Compound 13b
f1 (ppm)

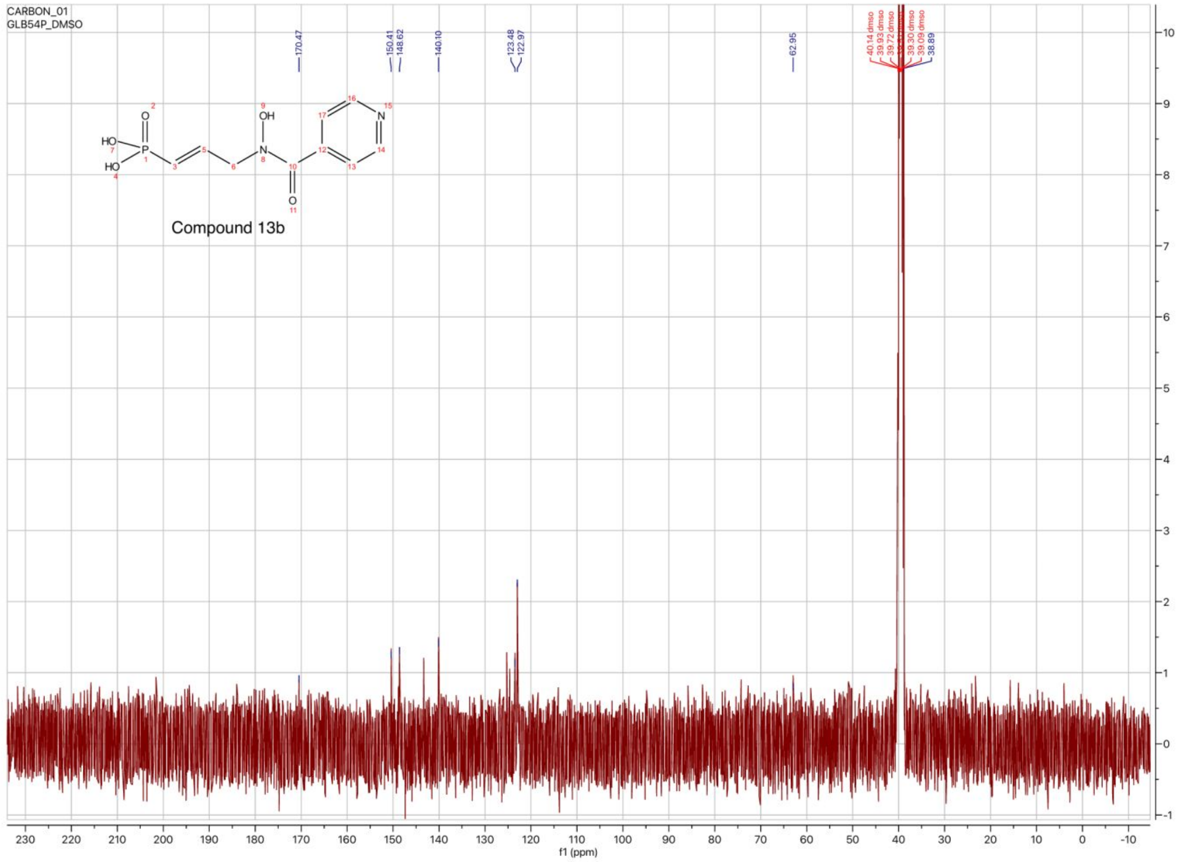
CARBON_01
GLB54P_DMSO
170.47
150.41
148.62
140.10
123.48
122.97
62.95
40.14 dmso
39.93 dmso
39.72 dmso
39.30 dmso
39.09 dmso
38.89
Compound 13b
f1 (ppm)

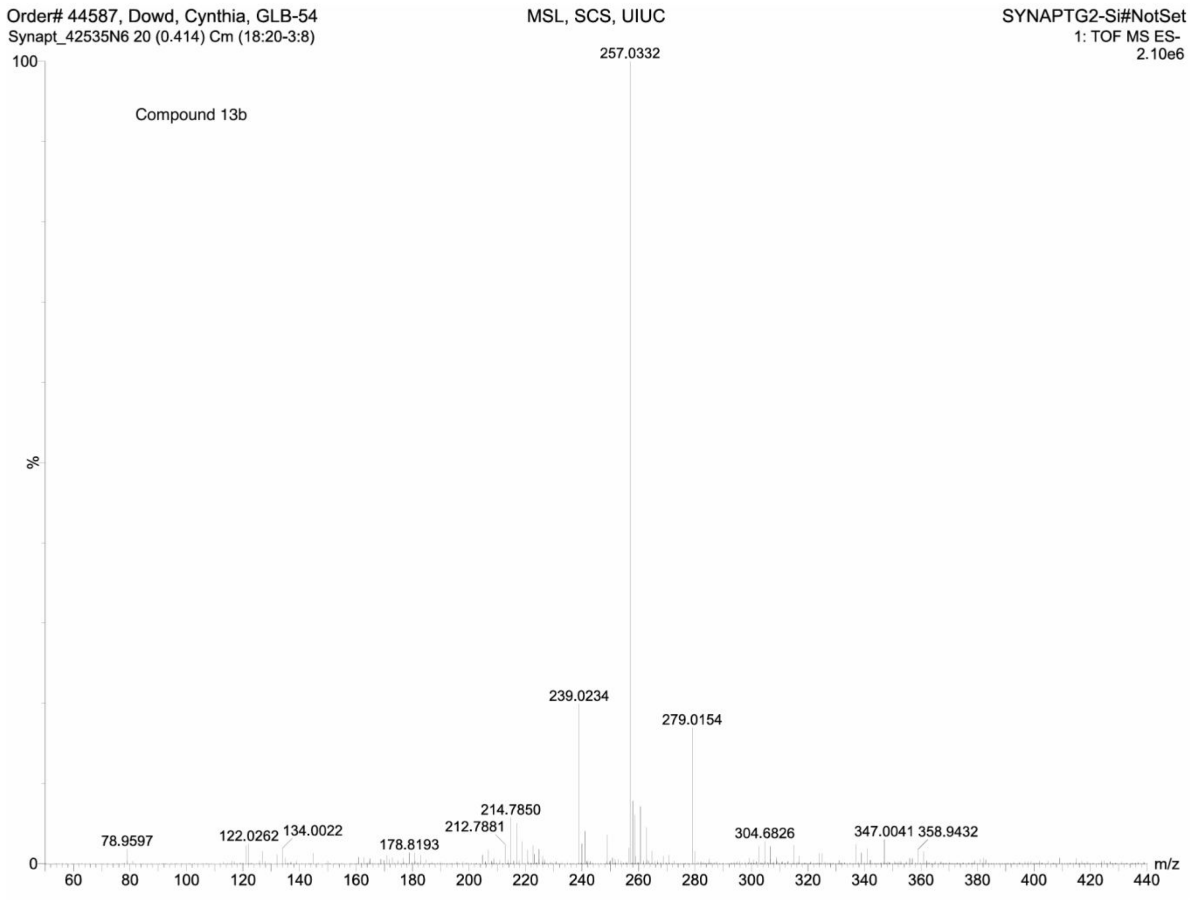

Diammonium [(1E)-3-[1-(3,4-dichlorophenyl)-N-hydroxyformamido]prop-1-en-1-yl]phosphonate (14b). ^{1}H NMR (400 MHz, CD_3OD) δ 7.78 – 7.71 (m, 1H), 7.00 – 6.89 (m, 2H), 6.28 – 6.14 (m, 1H), 5.88 – 5.76 (m, 1H), 4.23 (s, 2H). ^{13}C NMR (101 MHz, DMSO) δ 168.29, 135.51, 130.79, 129.49, 128.36, 126.75, 114.14, 113.48, 112.97, 55.29. HRMS (ESI$^+$) calculated for $C_{10}H_{11}Cl_2NO_5P$: 325.9752; found 325.9745 $[M+H]^+$.

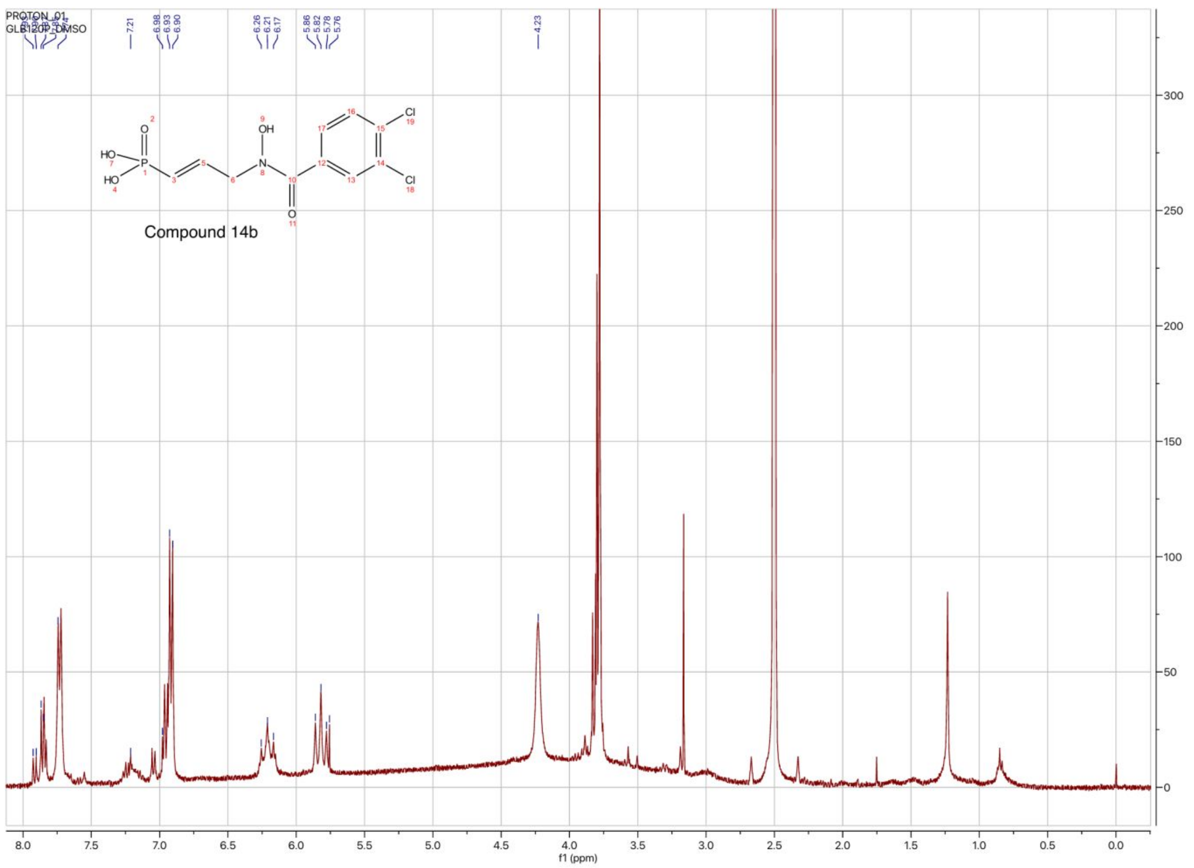
PROTON_01
GLB120P_DMSO
7.21
6.98
6.93
6.90
6.26
6.21
6.17
5.86
5.82
5.78
5.76
4.23
Compound 14b
f1 (ppm)

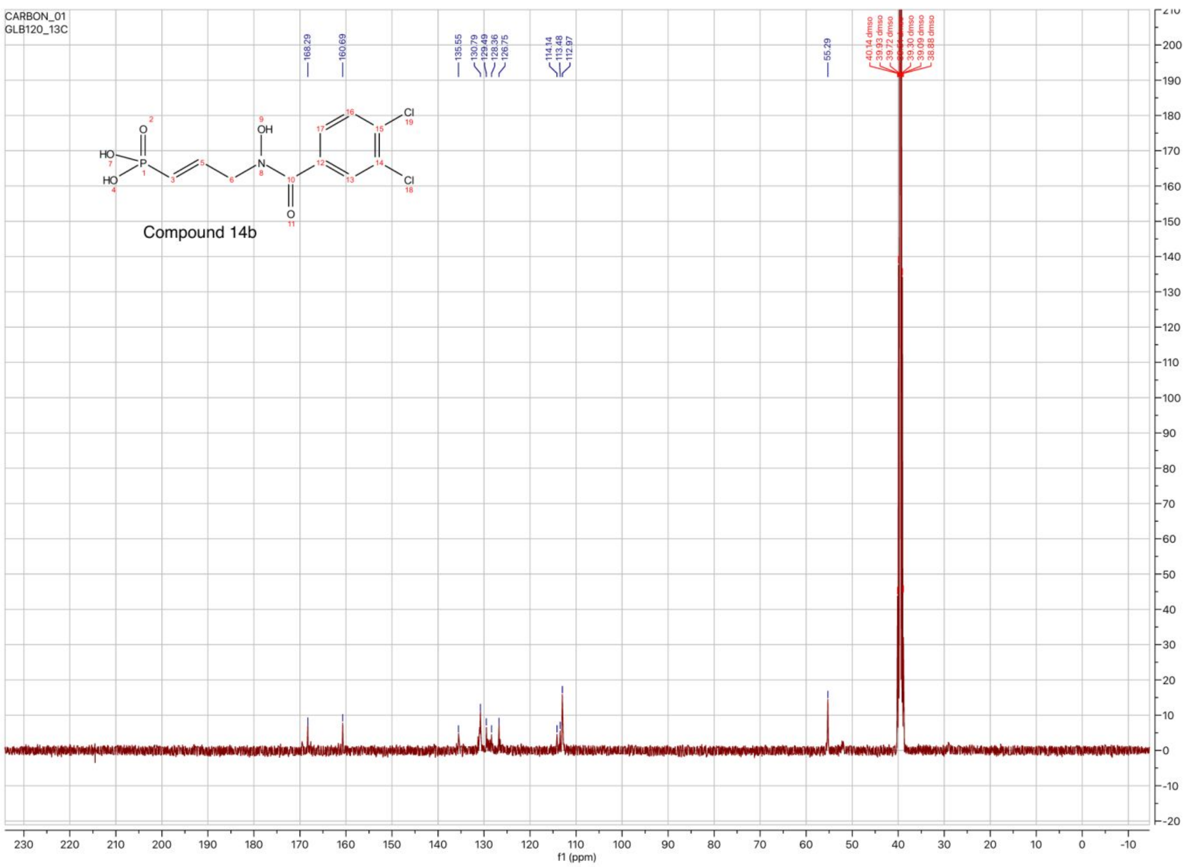

CARBON_01
GLB120_13C
168.29
160.69
135.55
130.79
129.49
128.36
126.75
114.14
113.48
112.97
55.29
40.14 dmso
39.93 dmso
39.72 dmso
39.30 dmso
39.09 dmso
38.88 dmso
Compound 14b
f1 (ppm)

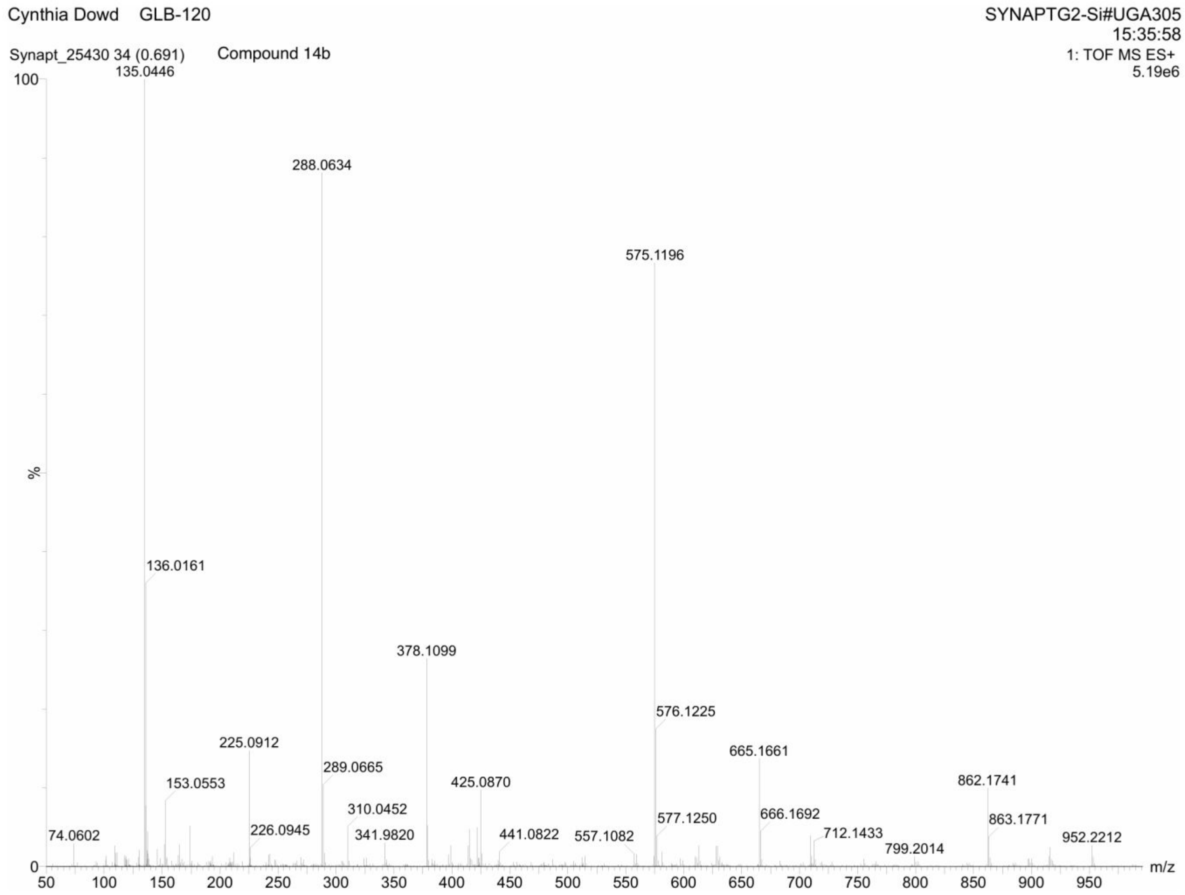

Diammonium [(1E)-3-[1-(3-fluorophenyl)-N-hydroxyformamido]prop-1-en-1-yl]phosphonate (15b). ^{1}H NMR (400 MHz, DMSO) δ 7.55 – 7.38 (m, 3H), 7.24 (td, J = 8.7, 2.6 Hz, 1H), 6.29 – 6.14 (m, 1H), 5.84 (t, J = 16.2 Hz, 1H), 4.45 – 4.10 (m, 2H). HRMS (ESI^{+}) calculated for $C_{10}H_{12}FNO_5P$: 276.0437; found 276.0435 $[M+H]^{+}$.

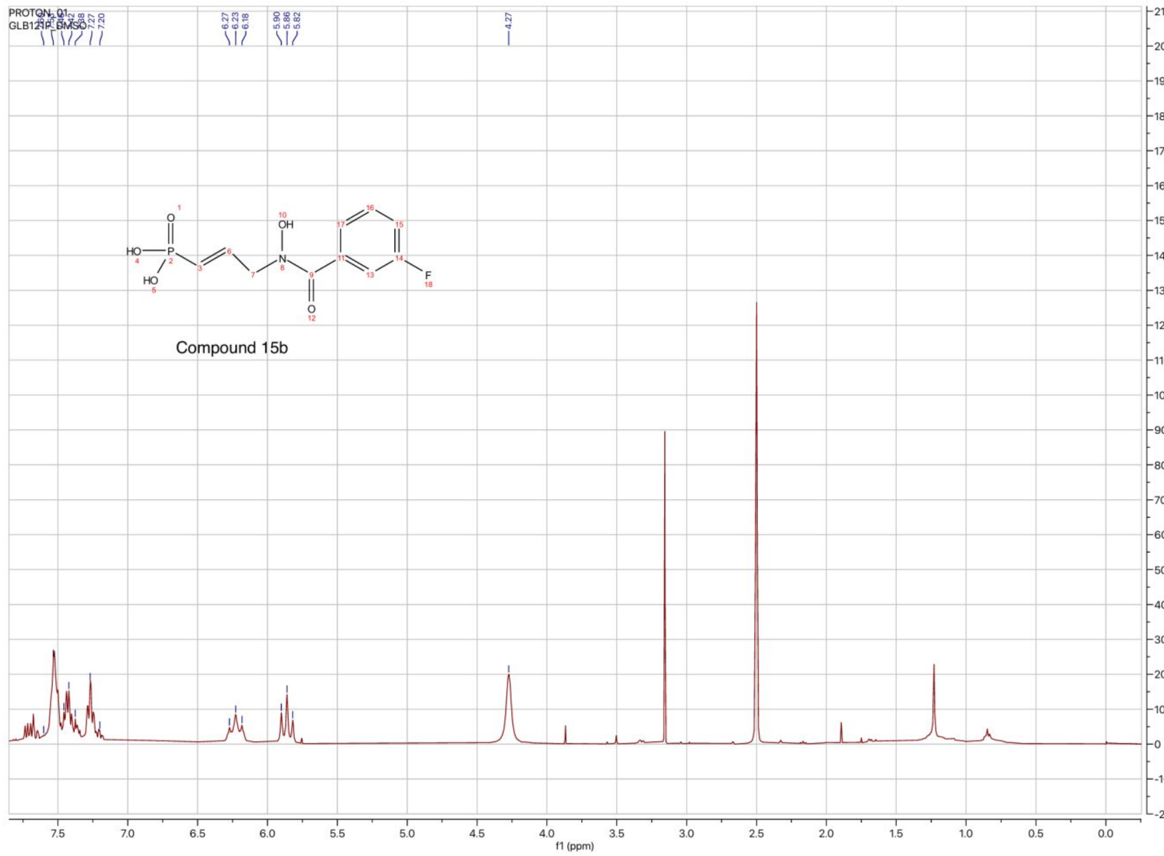
PROTON_01
GLB121F_DMSO
6.27
6.23
6.18
5.90
5.86
5.82
4.27
Compound 15b
f1 (ppm)

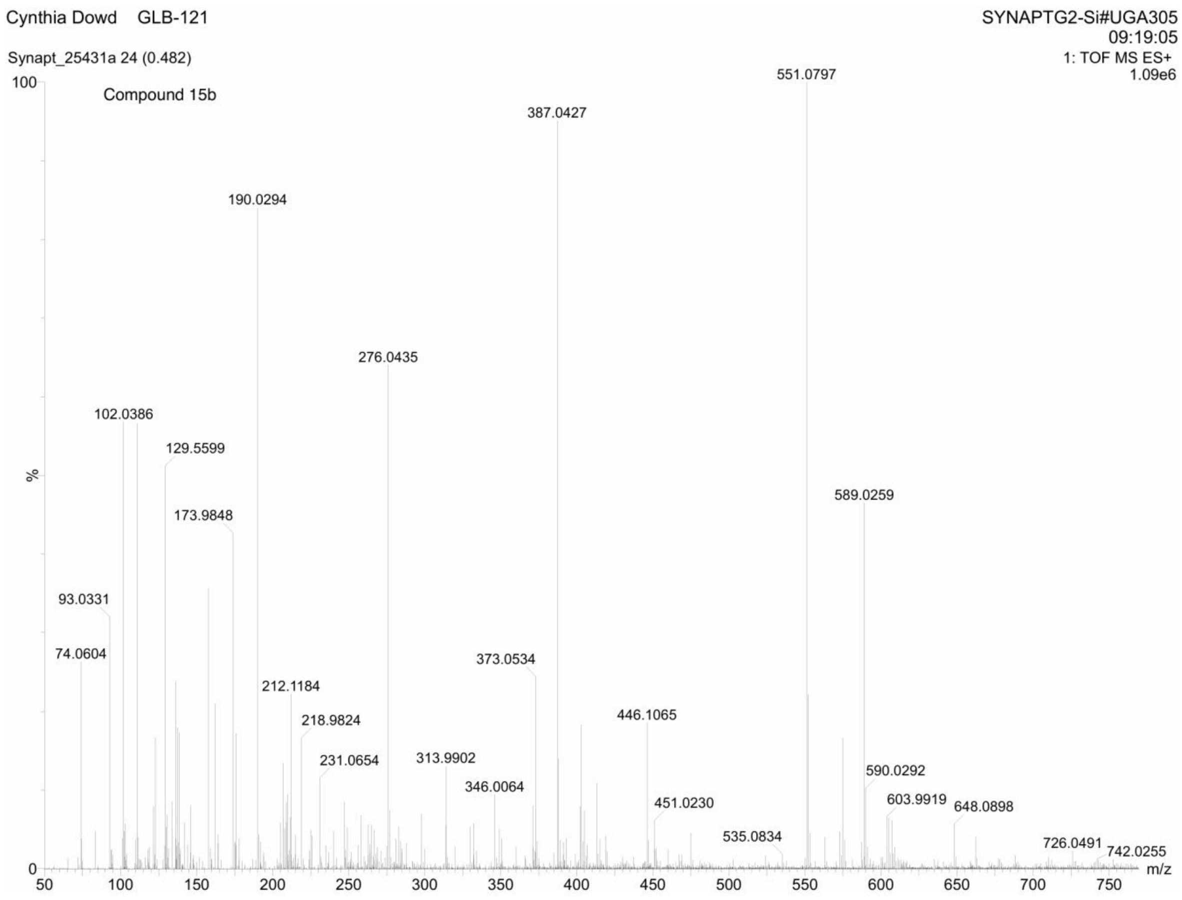

Diammonium [(1E)–3–[N–hydroxy–1–(4–methoxyphenyl)formamido]prop–1–en–1–yl] phosphonate (16b). ^{1}H NMR (400 MHz, DMSO) δ 7.24 – 6.91 (m, 2H), 6.82– 6.68 (m, 2H), 5.51 (t, J = 17.9 Hz, 1H), 5.22 (m, 1H), 4.28 (s, 2H), 3.54 (s, 3H). HRMS (ESI+) calculated for C11H15NO6P 288.0632, found 288.0634 [M+H].

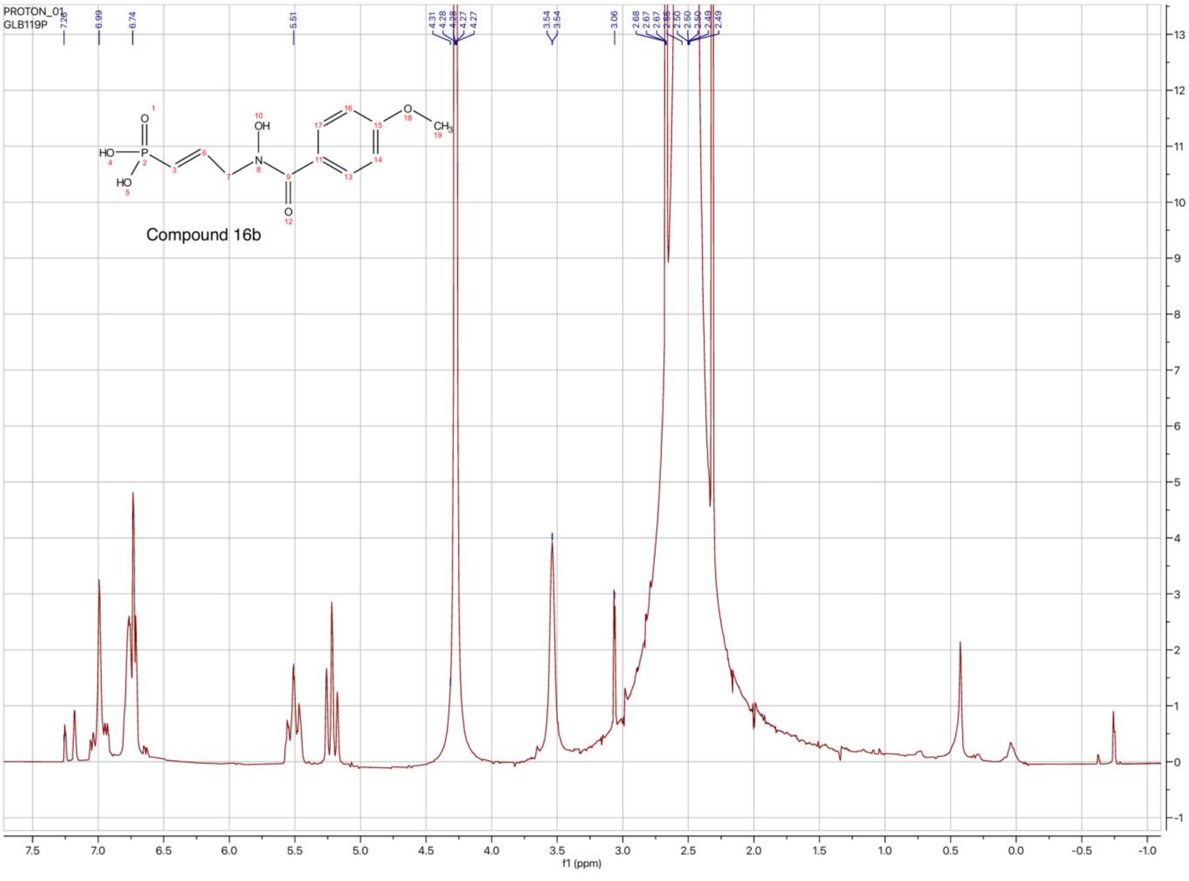
PROTON_01
GLB119P
Compound 16b
f1 (ppm)

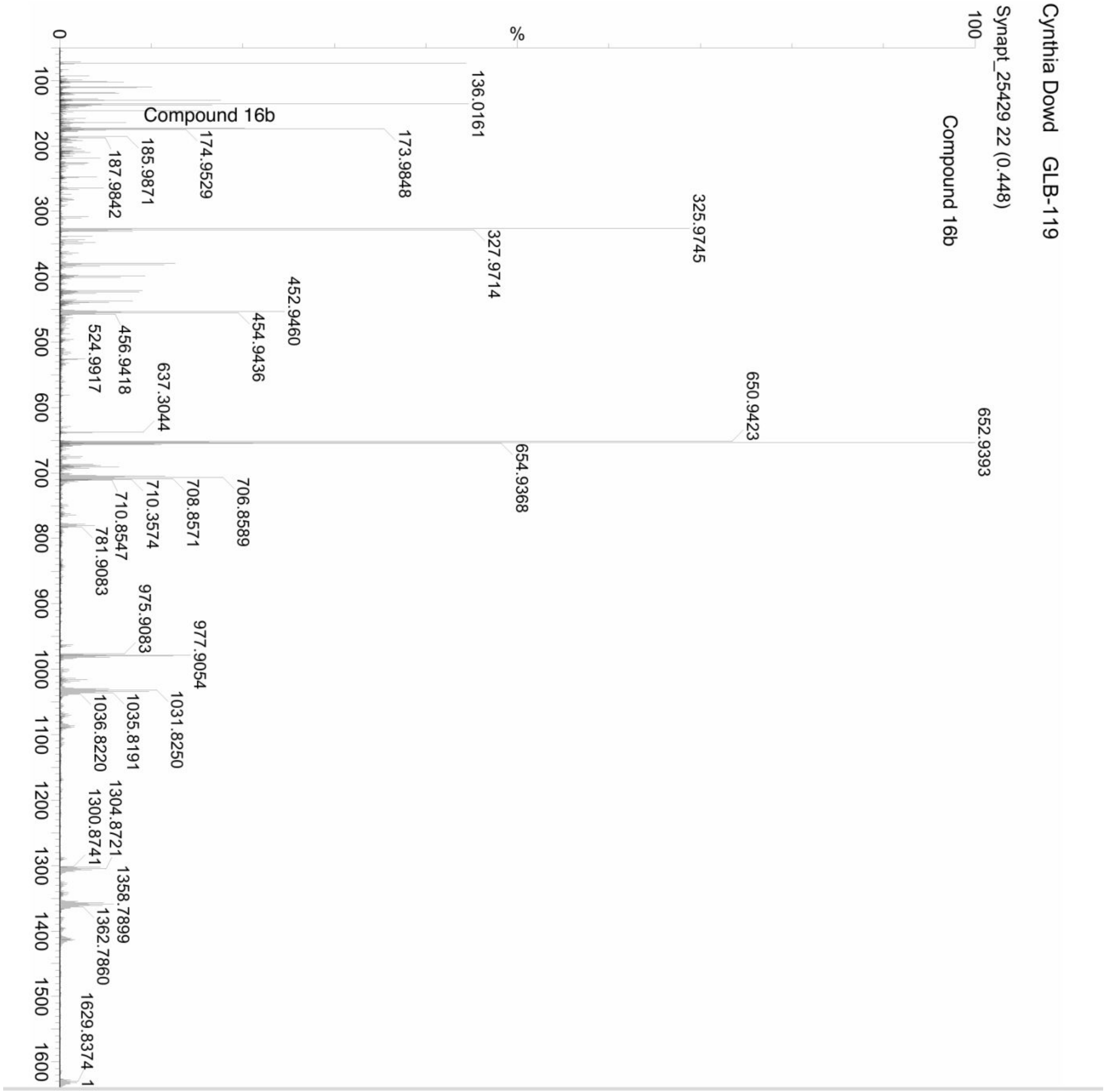

Diammonium [(1E)–3–{N–hydroxy–1–[4–(methylsulfanyl)phenyl] formamido}prop–1–en–1–yl] phosphonate (17b). ^{1}H NMR (400 MHz, DMSO) δ 7.88 – 7.63 (m, 2H), 7.31 – 7.15 (m, 2H), 6.21 (t, J = 17.9 Hz, 1H), 5.81 (t, J = 16.3 Hz, 1H), 4.37 – 4.09 (m, 2H), 2.47 (s, 3H). ^{13}C NMR (101 MHz, DMSO) δ 168.02, 140.98, 130.89, 129.69, 129.29, 124.78, 124.73, 124.36, 14.19. HRMS (ESI^{+}) calculated for $C_{11}H_{15}NO_5PS$: 304.0409; found 304.0402 $[M+H]^{+}$.

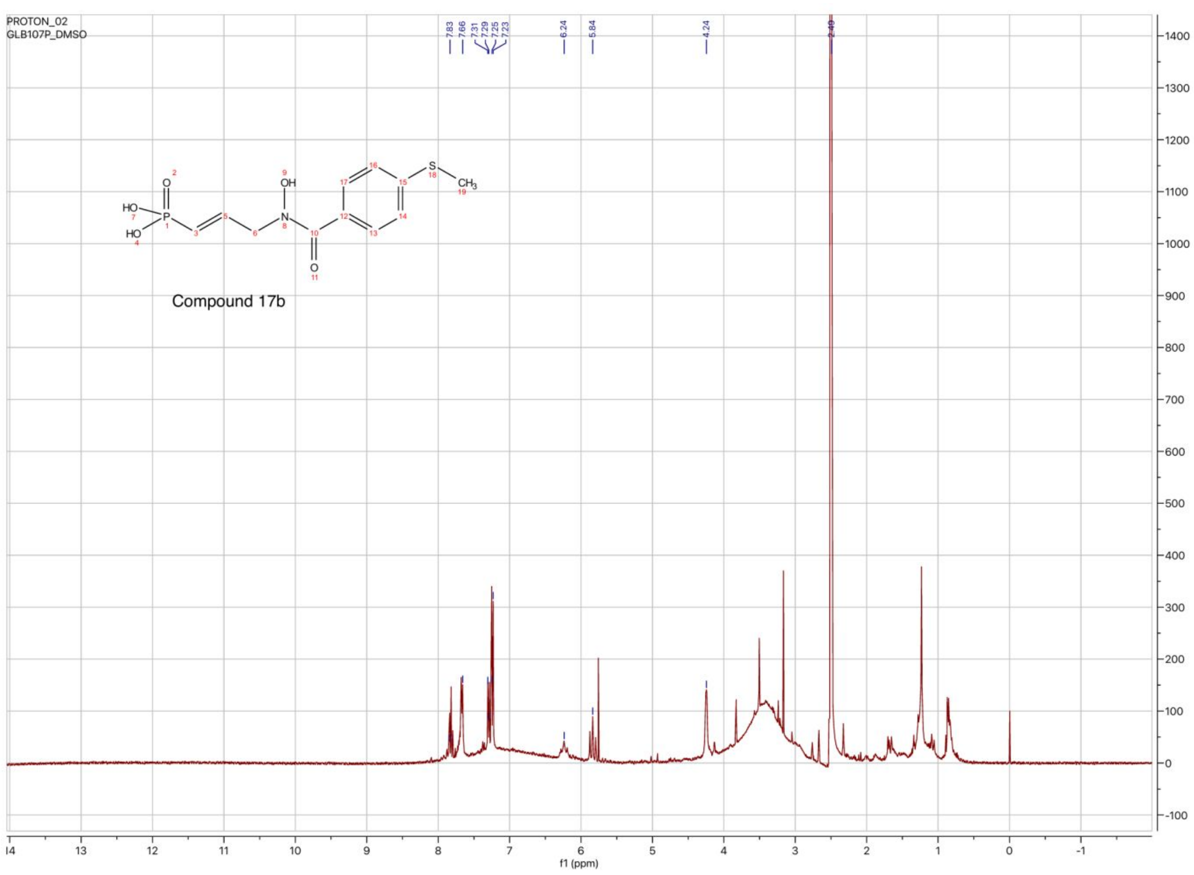

PROTON_02
GLB107P_DMSO
7.83
7.66
7.31
7.29
7.25
7.23
6.24
5.84
4.24
2.49
Compound 17b
f1 (ppm)

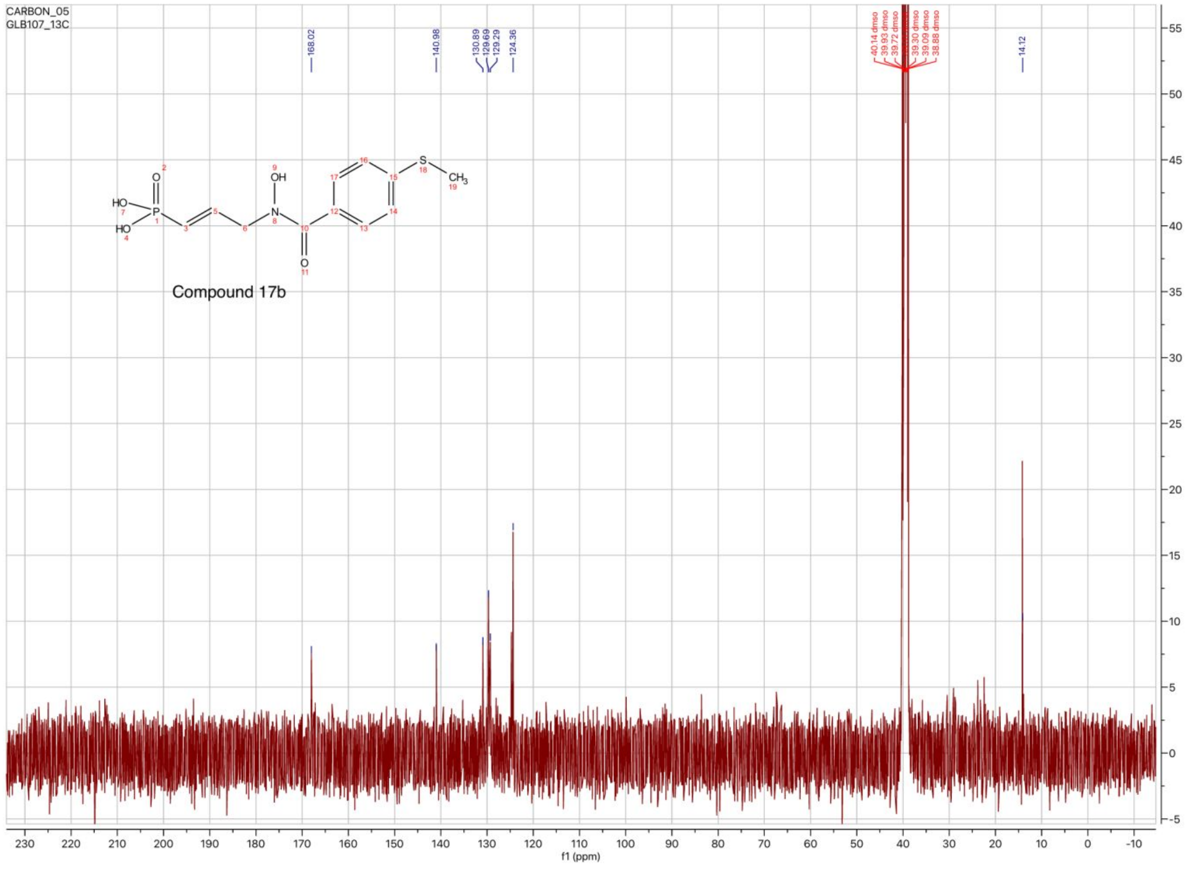
CARBON_05
GLB107_13C
168.02
140.98
130.89
129.69
129.29
124.36
40.14 dmso
39.93 dmso
39.72 dmso
39.30 dmso
39.09 dmso
38.88 dmso
14.12
Compound 17b
f1 (ppm)

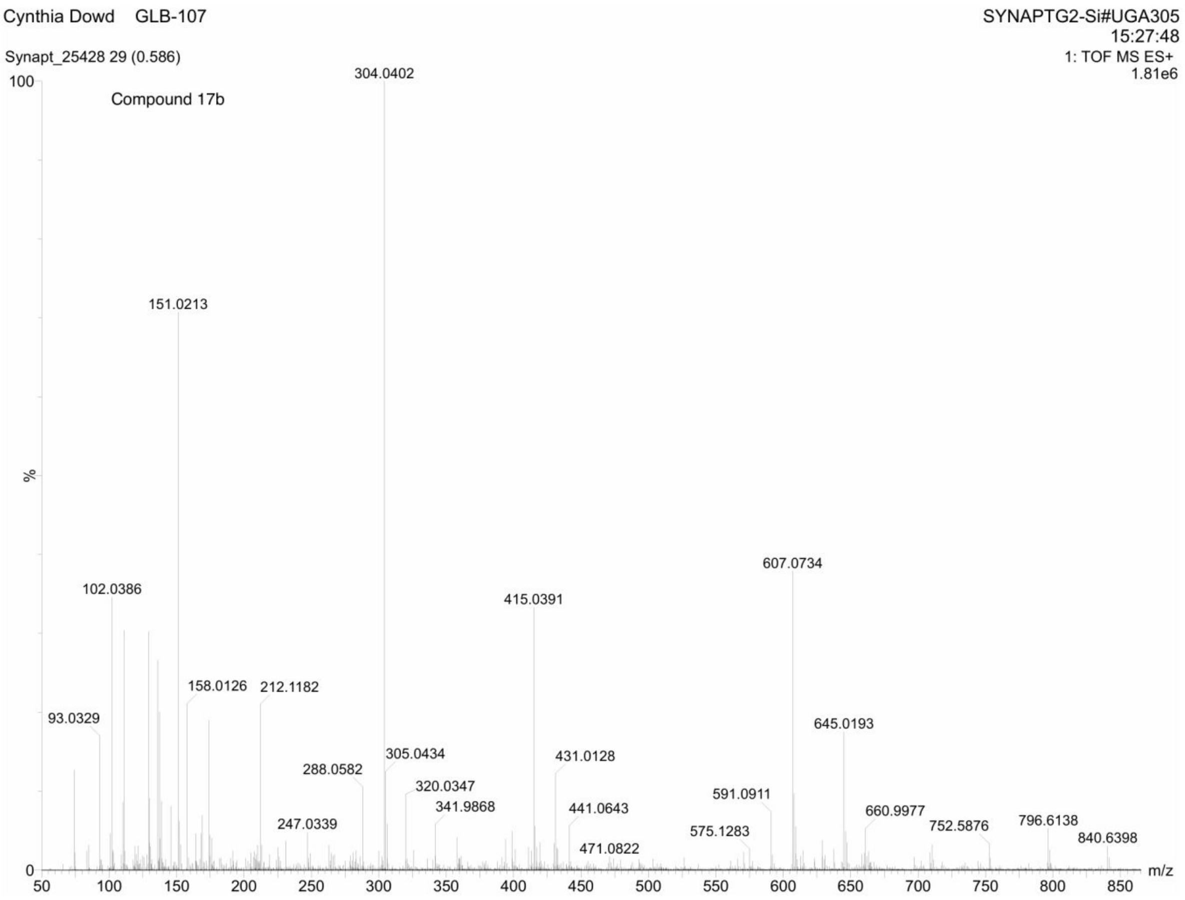

Primary Sequence Alignment of select IspC Homologs.

```
PfIspC    MKKYIYIYFFFITITINDLVINNTSKCVSIERRKNNAYINYGIGYNGPDNKITKSRRCKR   60
MtIspC    ------------------------------------------------------------   0
FtIspC    ------------------------------------------------------------   0
AbIspC    ------------------------------------------------------------   0
YpIspC    ------------------------------------------------------------   0
KpIspC    ------------------------------------------------------------   0
EcIspC    ------------------------------------------------------------   0

PfIspC    IKLCKKDLIDIGAIKKPINVAIFGSTGSIGTNALNIIRECNKIENVFNVKALYVNKSVN    119
MtIspC    -----MTNSTDGRADGRLRVVVLGSTGSIGTQALQVIADNPD---RFEVVGLAAGGAHLD   52
FtIspC    -------------MFKKTKITILGATGSIGDSTLAVIRET-N---DFEVFALTAFSNV-E   42
AbIspC    ---------------MTQSVCILGVTGSIGRSTLKILGQHPD---KYSVFAVSAHSRI-S   41
YpIspC    ----------------MKQLTILGSTGSIGNSTLSVVRANPE---LFKVTALVAGRNV-R   40
KpIspC    ----------------MKQLTVLGSTGSIGCSTLDVVRHNPG---RFSVAALVAGKNV-D   40
EcIspC    ----------------MKQLTILGSTGSIGCSTLDVVRHNPE---HFRVVALVAGKNV-T   40
                             : ::* ***** .:* ::         : * .: .
```

```
PfIspC   ELYEQAREFLPEYLCIHDKSVYEELKELVKNIKDYKPIILCGDEGMKEICSSNSIDKIVI   179
MtIspC   TLLRQRAQTGVTNIAVADEHAAQRV----------GDIPYHGSDAATRLVEQTEADVVLN   102
FtIspC   KLAELCQEFKPKFAVVPDLSKKQK----LQS-LVTDVEVLVGESGLEKVSSLAEIDIVMS   97
AbIspC   ELVEICKQFRPKVVVVPEQKIAELKTLFAQQ-NISDIDVLAGQEGLVDIASHTDVDIVMA   100
YpIspC   EMAQQCLEFSPRYAAMSDEHSAKSLRLLLAE-QGSDTEVYSGETAACELAALDDVDQVMA   99
KpIspC   RMVEQCLEFTPRYAVMDDAQSAERLRTRLHE-HGSRTEVLSGQQAAAEVAALDEVDQVMA   99
EcIspC   RMVEQCLEFSPRYAVMDDEASAKLLKTMLQQ-QGSRTEVLSGQQAACDMAALEDVDQVMA   99
         : .   :       : :    :                    *. .   :     . * ::

PfIspC   GIDSFQGLYSTMYAIMNNKIVALANKESIVSAGFFLKKLLNIHKNAKIIPVDSEHSAIFQ   239
MtIspC   ALVGALGLRPTLAALKTGARLALANKESLVAGGSLVLRAAR-P--GQIVPVDSEHSALAQ   159
FtIspC   AIVGIAGLKPTFAAAKAGKKILLANKESLVTAGHLLIDEVV-KNNAQLIPVDSEHNAIFQ   156
AbIspC   AIVGAAGLLPTLAAVKAGKRVLLANKEALVMSGEIMMQAAR-DHQALLLPVDSEHNAIFQ   159
YpIspC   AIVGIAGLPSTLAAIRAGKQVLLANKESLITCGKLFMDEVK-RSRAQLLPIDSEHNAIFQ   158
KpIspC   AIVGAAGLVPTLAAIRAGKTVLLANKESLVTCGRLFMEAVQ-QSGARLLPVDSEHNAIFQ   158
EcIspC   AIVGAAGLLPTLAAIRAGKTILLANKESLVTCGRLFMDAVK-QSKAQLLPVDSEHNAIFQ   158
          .: .  **  *: *    .  : *****:::  *  :.          . ::*:****.*: *

PfIspC   CLDNNKVLKTKCLQDNFSKINNINKIFLCSSGGPFQNLTMDELKNVTSENALKHPKWKMG   299
MtIspC   CLRGGT-------------PDEVAKLVLTASGGPFRGWSAADLEHVTPEQAGAHPTWSMG   206
FtIspC   CIDNHDK---KCL-------PEIDKIILTASGGPFRDKQLHELTDVTPEQACNHPNWQMG   206
AbIspC   SLPHNYL---QA-DRTGQPQLGVSKILLTASGGPFLNHSLEQLVHVTPQQACKHPNWSMG   215
YpIspC   SLPERIQ---RQLGYSSLNENGVSRIILTGSGGPFRETPLSQFSDVTPDQACAHPNWSMG   215
KpIspC   SMPETIQ---QHLGYADLAQNGVSSILLTGSGGPFRETAVAELAAMTPDQACRHPNWSMG   215
EcIspC   SLPQPIQ---HNLGYADLEQNGVVSILLTGSGGPFRETPLRDLATMTPDQACRHPNWSMG   215
          .:                         :  :.* .*****        ::  :* ::*  **.*.**

PfIspC   KKITIDSATMMNKGLEVIETHFLFDVDYNDIEVIVHKECIIHSCVEFIDKSVISQMYYPD   359
MtIspC   PMNTLNSASLVNKGLEVIETHLLFGIPYDRIDVVVHPQSIIHSMVTFIDGSTIAQASPPD   266
FtIspC   RKISVDSSTMVNKALEVIEAYWLFSVSADKIGVLIHPQSVTHSMVRYVDGSYIAQLGVPD   266
AbIspC   QKISVDSATLMNKGLELIEACHLFSISEHFVTVVVHPQSIIHSMVQYVDGSTLAQMGNPD   275
YpIspC   RKISVDSATMMNKGLEYIEARWLFNASAEQIEVVLHPQSVIHSMVRYHDGSILAQMGTPD   275
KpIspC   RKISVDSATMMNKGLEYIEARWLFNASAQQMEVLIHPQSVIHSMVRYQDGSVLAQLGEPD   275
EcIspC   RKISVDSATMMNKGLEYIEARWLFNASASQMEVLIHPQSVIHSMVRYQDGSVLAQLGEPD   275
             :::*::::**.** **:  **.        : *::* :.: ** * : * * ::*   **

PfIspC   LNASNEIANNLFLNNKIKYFDISSIISQVLESFNSQKVSENSEDLMKQILQIHSWAKDKA   478
MtIspC   YNAANEEAAAAFLAGRIGFPAIVGIIADVLHAADQWAVE---PATVDDVLDAQRWARERA   382
FtIspC   FNAANEELVAAFLNKKIKYLEIIEVNKKVTKELN--FEN---PKNIEEVFEIDRKTREYV   380
AbIspC   LNAANEIAVEAFLMERIGFTSIPQVVEHTLEKLE--NAA---AESIECILDKDKVARSVA   389
YpIspC   LNAANEISVMAFLDSKIRFTDIEVINRTVVEGLL--LSE---PTSVEEVLVIDRKARDVA   389
KpIspC   LNAANEESVAAFLHGDIRFTDIAAVNLAVLDKMD--LQE---PQSIDDVLVIDAEARAIA   389
EcIspC   LNAANEITVAAFLAQQIRFTDIAALNLSVLEKMD--MRE---PQCVDDVLSVDANAREVA   389
          **:**     **   * :  *  :    . .                :. ::  .  ::  .

PfIspC   MQIPILYSLTWPDRIKTNLKPLDLAQVSTLTFHKPSLEHFPCIKLAYQAGIK-GNFYPTV   418
MtIspC   MKLPISLALGWPRRVSGAAAACDFHTASSWEFEPLDTDVFPAVELARQAGVA-GGCMTAV   325
FtIspC   MKTPIANAMYYPKRGSVNVESLDFTKY-QLTFREACFERFEALKIVFNNLQNKNYAANIV   325
AbIspC   MCTPIAHALAWPERLQTNVPALDLFEYSQLNFQAPDTQKFPALNLARQAMRA-GGLAPTI   334
YpIspC   MRTPIAHAMAYPMRVSSGVAPLDFCKVGALTFTTPDYQRYPCLKLAIDACNA-GQAATTA   334
KpIspC   MRTPIAHTMGWPQRLNSGVKPLDFCQLSNLSFSAPDYTRYPCLKLAMDAFDV-GQAATTT   334
EcIspC   MRTPIAHTMAWPNRVNSGVKPLDFCKLSALTFAAPDYDRYPCLKLAMEAFEQ-GQAATTA   334
         *  **  :: :* * .        *:         *          : .:::. :        .

PfIspC   TDIYNKHNS----------------------   487
MtIspC   QRAVSGMASVAIASTAKPGAAGRHASTLERS   413
FtIspC   DSVLG--------------------------   385
AbIspC   QQYISSIGG----------------------   398
YpIspC   AQVIAKLNN----------------------   398
KpIspC   HQQLQRLVAQA--------------------   400
EcIspC   RKEVMRLAS----------------------   398
```

Figure S1. Multiple sequence alignment (MSA) of the amino acid residues of IspC homologs. The MSA was performed using Clustal Omega.[1] In the AbIspC crystal

structure, we reported previously (PDB ID: 7S04)[2], Asp151, Glu153, and Glu231 are seen to directly coordinate the active site Mg^{2+} (numbering follows AbIspC, Uniprot ID: B7H1U5). The equivalent residues are 100% conserved across all the IspC homologs compared (highlighted in cyan). In AbIspC (PDB ID: 7S04), the FR (**2a**) phosphonate group forms hydrogen bonds with Ser186, Ser222, Asn227, Lys228, and two water molecules. The equivalent residues across all aligned IspC homologs are 100% conserved (highlighted in green).[2] Trp 212, Met214 (highlighted in yellow), and Met276 (framed in purple) residues that protect FR(**2a**) from the bulk solvent[3,4] are 100 % conserved among the IspC homologs compared. Ala185 of AbIspC, the residue in the general active site of AbIspC, which was not interacting with FR(**2a**) in the structure we reported earlier (7S04)[2], is replaced by Ser in PfIspC (highlighted in red). Interestingly, this serine was indicated to form a hydrogen bond with the phosphonate moiety of FR(**2a**).[3,4] Name and UniProt IDs of the enzymes: PfIspC = *Plasmodium falciparum* IspC: Q8IKG4, MtIspC = *Mycobacterium tuberculosis* IspC: P9WNS1, FtIspC = *Francisella tularensis* IspC: A0A806QMF1, AbIspC = *Acinetobacter baumannii* IspC: B7H1U5, YpIspC = *Yersinia pestis* IspC: Q8ZH62, KpIspC = *Klebsiella pneumoniae* IspC: A6T4X5, EcIspC = *Escherichia coli* IspC: P45568**.**

Table S1. The Percent Identity Matrix for the IspC Homologs from the Multiple Sequence Alignment Performed Using Clustal Omega. AbIspC showed relatively moderate amino acid sequence similarity with KpIspC (49.62%), FtIspC (45.14%), and YpIspC (48.87%), while its similarity to EcIspC is slightly higher at 50.38%. In contrast, AbIspC showed lower amino acid sequence similarity with MtIspC (42.33%) and PfIspC (35.93%). KpIspC showed relatively high amino acid sequence similarity with YpIspC (71.86%) and EcIspC (80.90%). The Uniprot IDs of the IspC enzymes are as described in Figure S1 above.

	PfIspC	MtIspC	FtIspC	AbIspC	YpIspC	KpIspC	EcIspC
1. PfIspC	-	34.62	37.24	35.93	36.43	35.68	35.93
2. MtIspC	34.62	-	37.37	42.33	40.05	40.90	41.91
3. FtIspC	37.24	37.37	-	45.14	51.18	49.34	49.61
4. AbIspC	35.93	42.33	45.14	-	48.87	49.62	50.38
5. YpIspC	36.43	40.05	51.18	48.87	-	69.35	71.86
6. KpIspC	35.68	40.90	49.34	49.62	69.35	-	80.90
7. EcIspC	35.93	41.91	49.61	50.38	71.86	80.90	-

Representative IC_{50} Curves of FOS (1a)/FR(2a) analogs generated against purified *A. baumannii I*spC (AbIspC) and *K. pneumoniae* IspC (KpIspC).

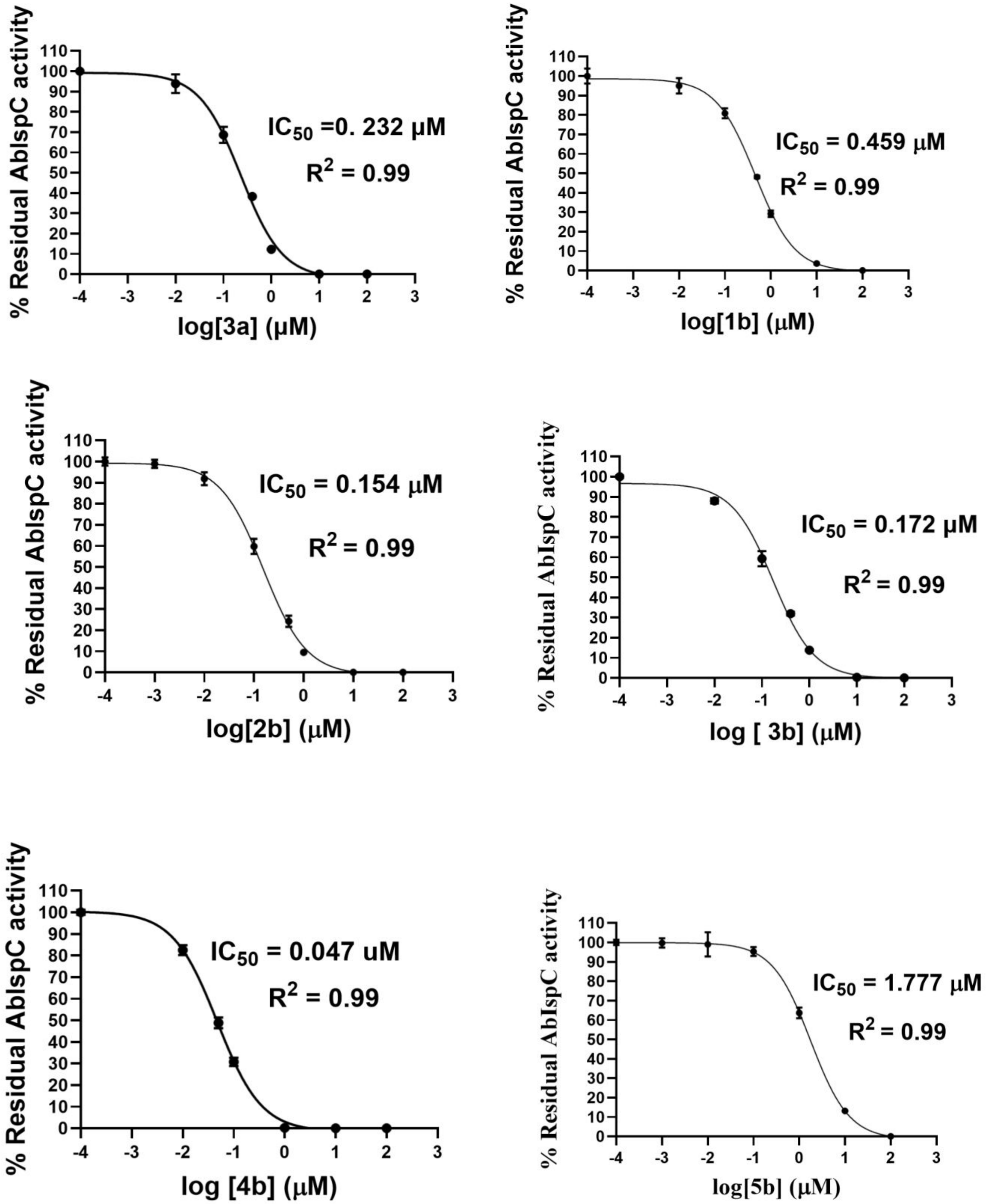

Figure S2. Half-maximal inhibitory concentrations (IC_{50}s) of *N*-acyl FOS(**1a**)/FR(**2a**) analogs determined against *A. baumannii* IspC (AbIspC).

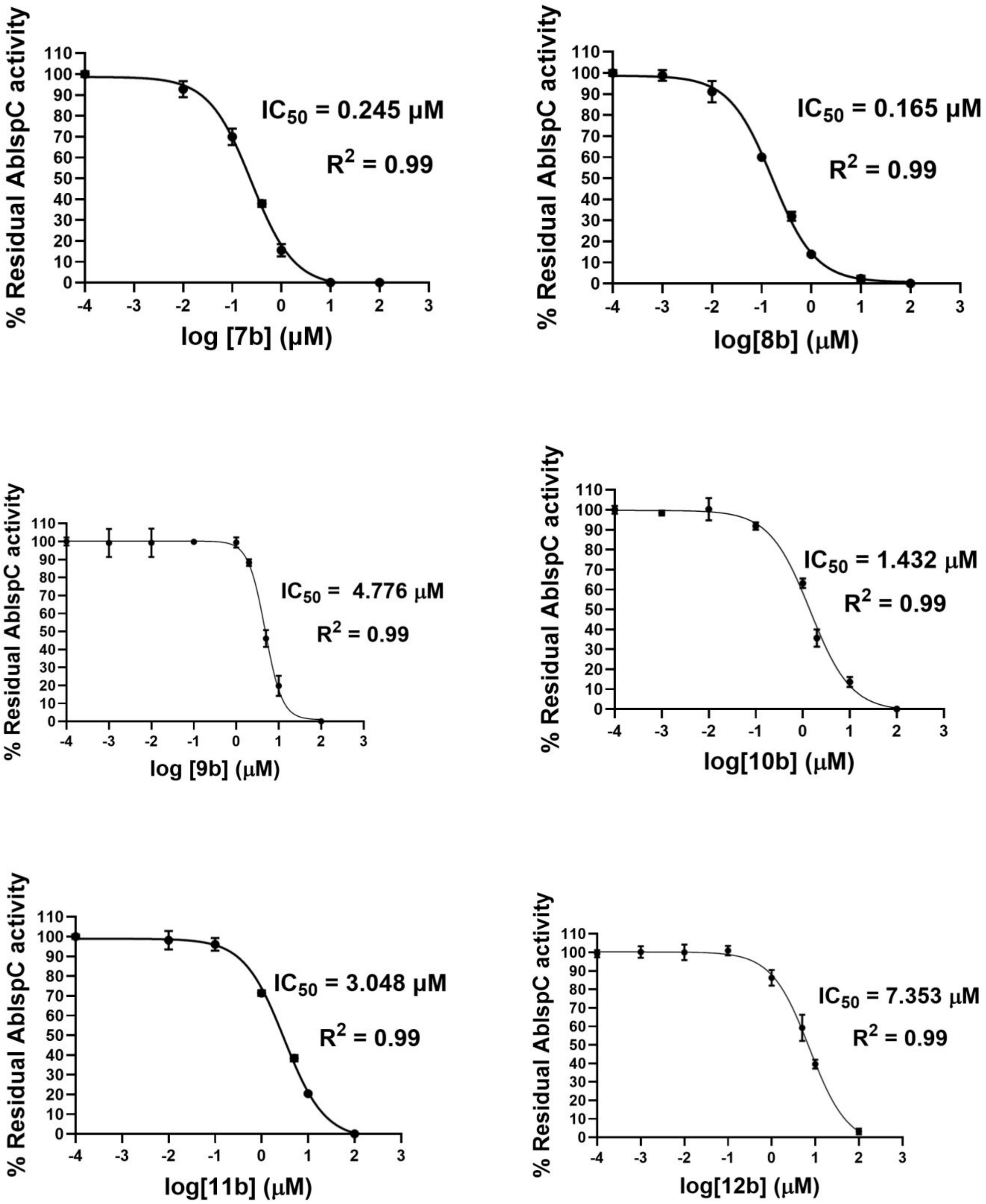

Figure S2 continued. Half-maximal inhibitory concentrations (IC_{50}s) of *N*-acyl FOS(**1a**)/FR(**2a**) analogs determined against *A. baumannii* IspC (AbIspC).

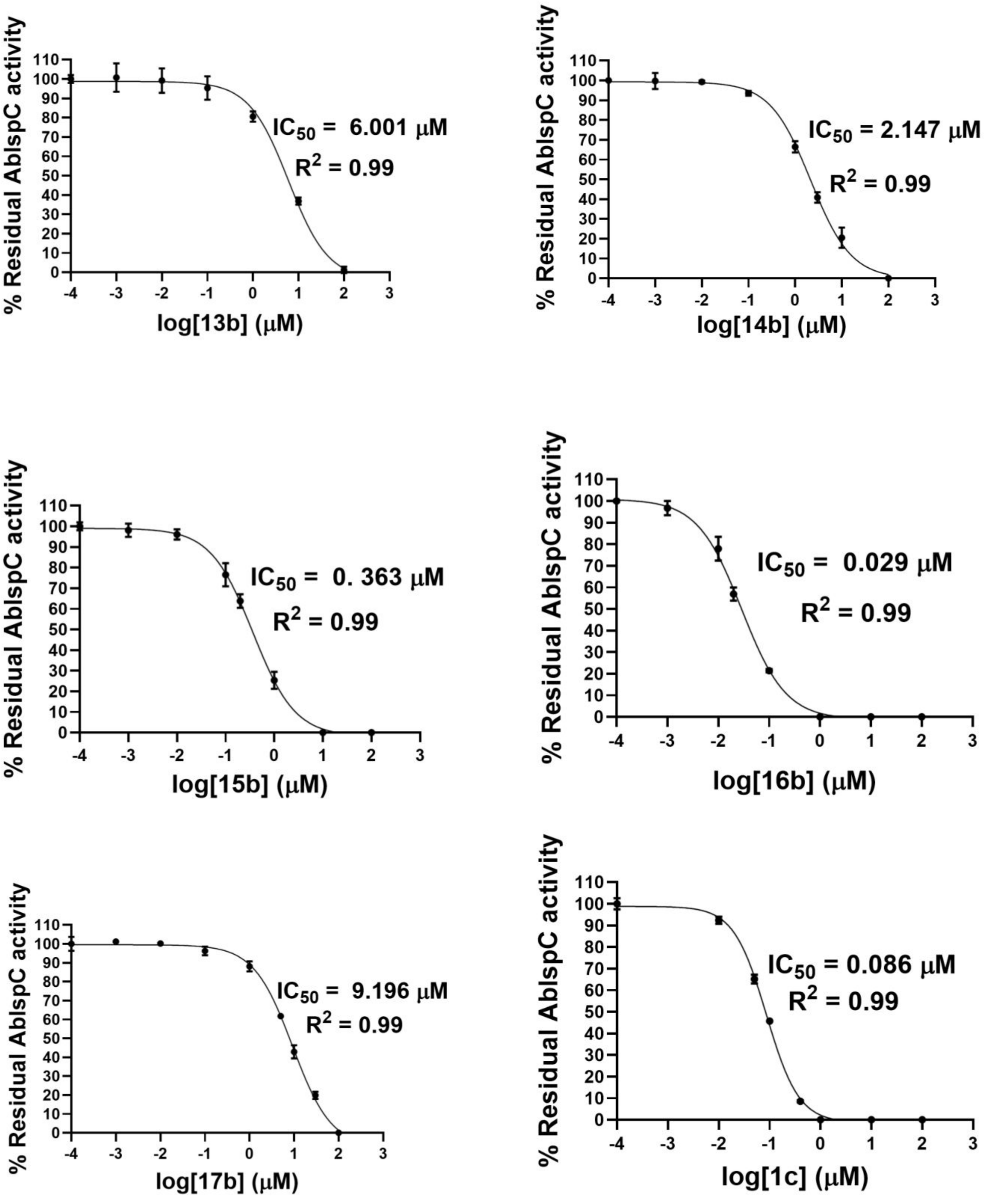

Figure S2 continued. Half-maximal inhibitory concentrations (IC_{50}s) of *N*-acyl FOS(**1a**)/FR(**2a**) analogs determined against *A. baumannii* IspC (AbIspC).

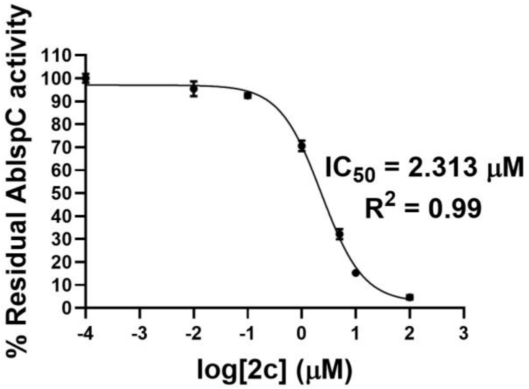

Figure S2 continued. Half-maximal inhibitory concentrations (IC_{50}s) of *N*-acyl FOS(**1a**)/FR(**2a**) analogs determined against *A. baumannii* IspC (AbIspC).

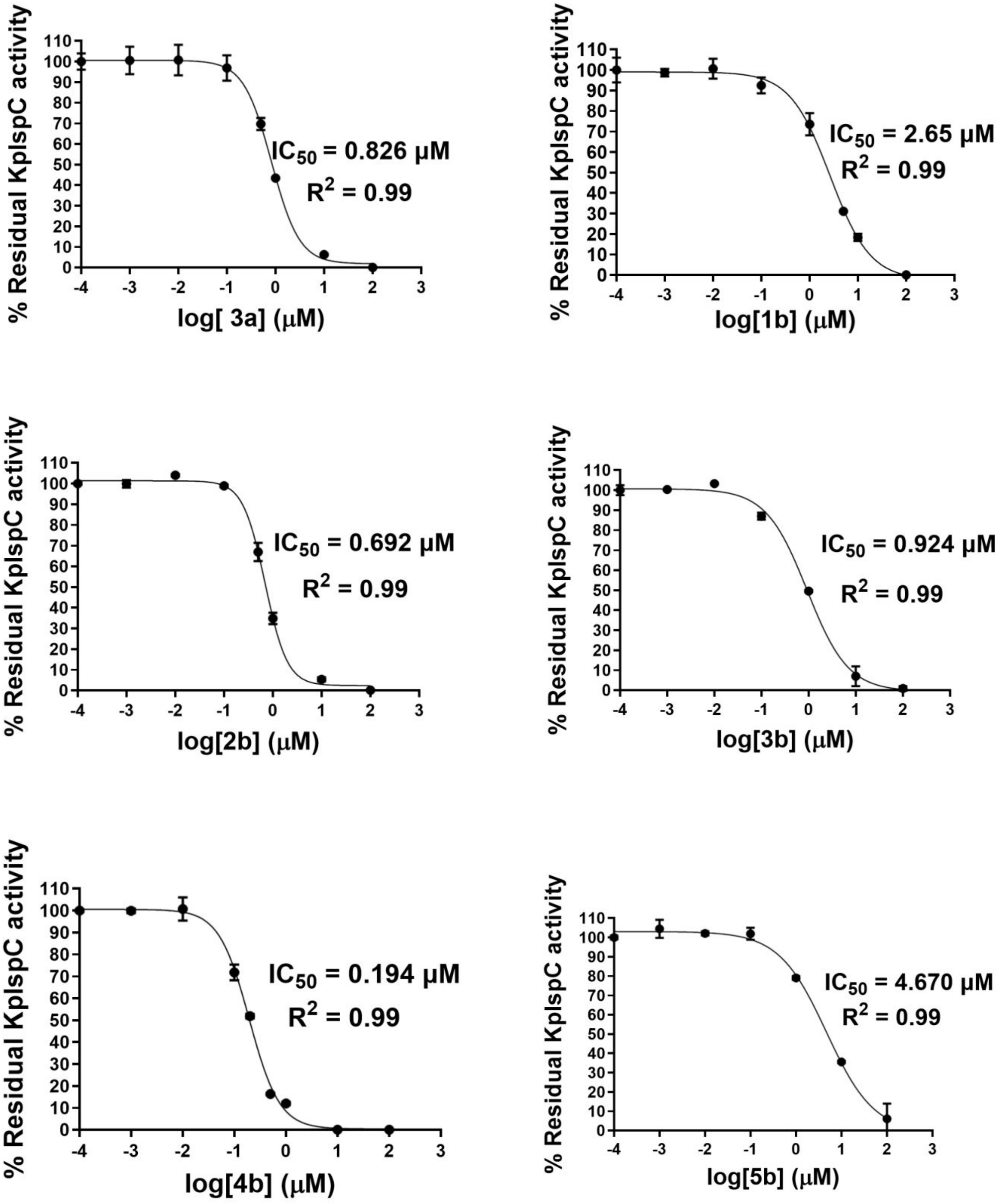

Figure S3. Half-maximal inhibitory concentrations (IC_{50}s) of *N*-acyl FOS(**1a**)/FR(**2a**) analogs determined against *K. pneumoniae* IspC (KpIspC).

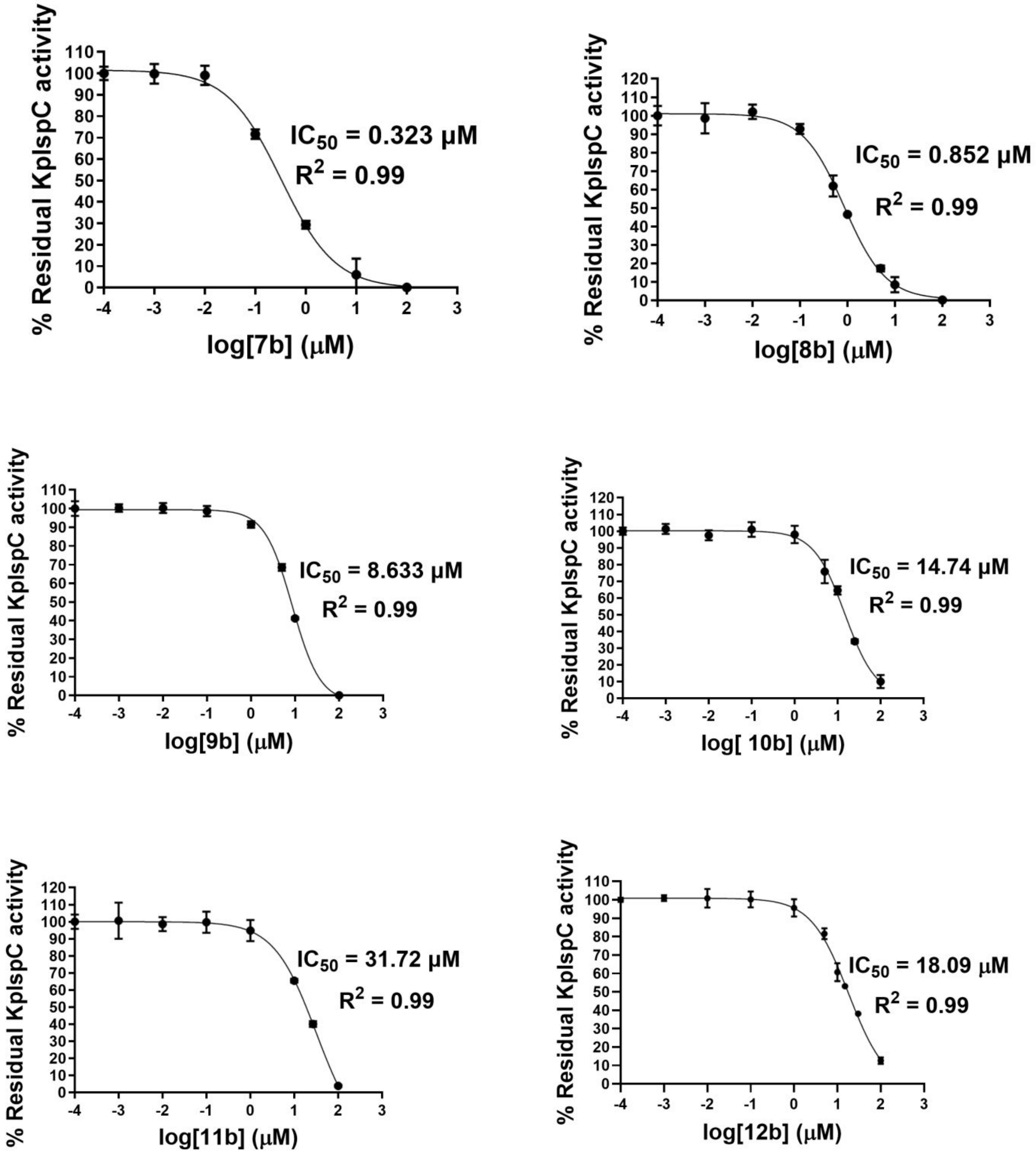

Figure S3 continued. Half-maximal inhibitory concentrations (IC_{50}s) of *N*-acyl FOS(**1a**)/FR(**2a**) analogs determined against *K. pneumoniae* IspC (KpIspC).

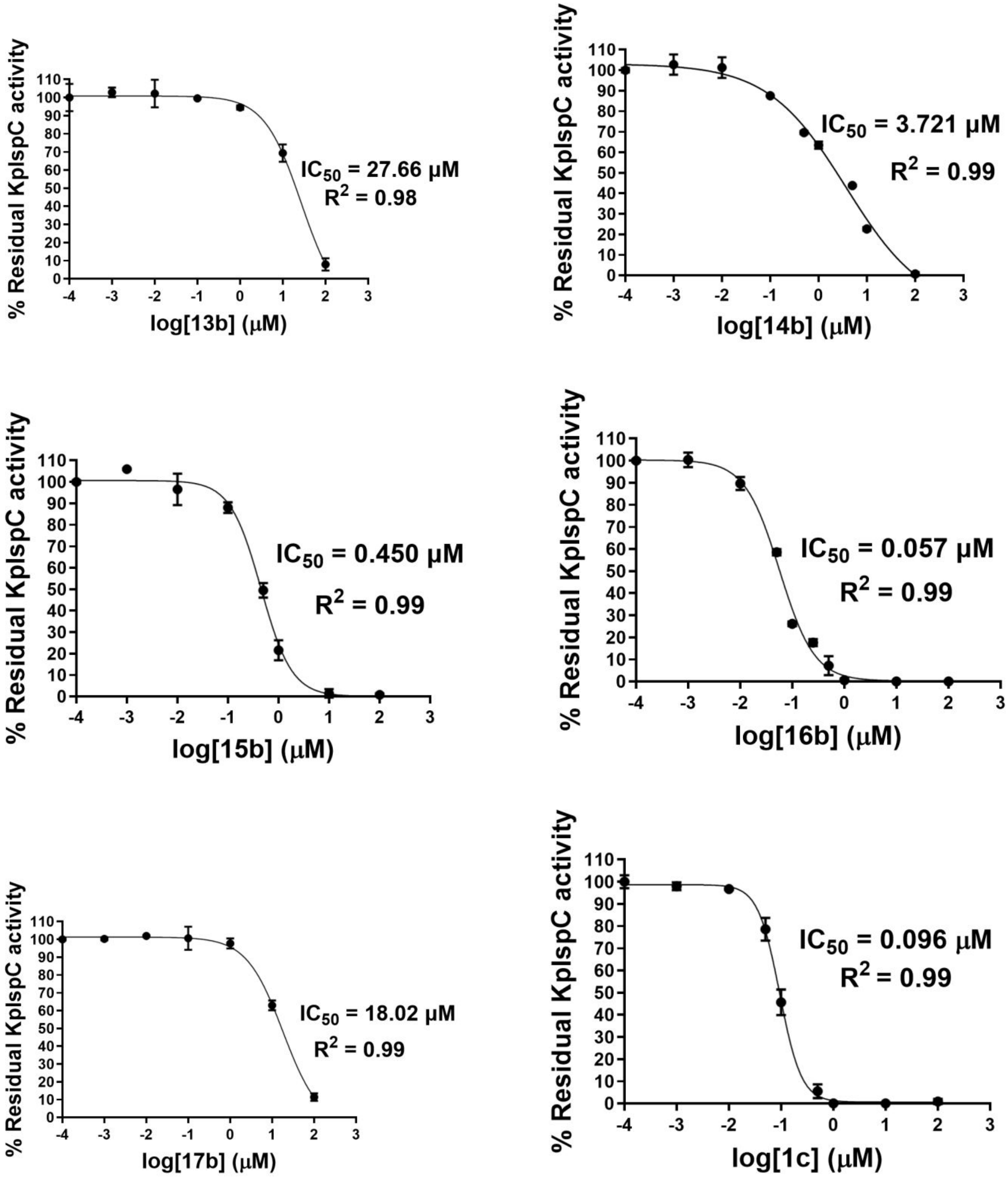

Figure S3 continued. Half-maximal inhibitory concentrations (IC_{50}s) of *N*-acyl FOS(**1a**)/FR(**2a**) analogs determined against *K. pneumoniae* IspC (KpIspC).

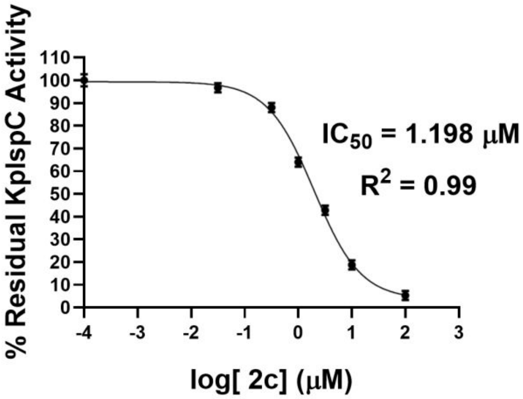

Figure S3 continued. Half-maximal inhibitory concentrations (IC_{50}s) of *N*-acyl FOS(**1a**)/FR(**2a**) analogs determined against *K. pneumoniae* IspC (KpIspC).

REFERENCES

(1) *Clustal Omega < Multiple Sequence Alignment < EMBL-EBI*. https://www.ebi.ac.uk/Tools/msa/clustalo/ (accessed 2023-07-16).

(2) Ball, H. S.; Girma, M. B.; Zainab, M.; Soojhawon, I.; Couch, R. D.; Noble, S. M. Characterization and Inhibition of 1-Deoxy-d-Xylulose 5-Phosphate Reductoisomerase: A Promising Drug Target in *Acinetobacter baumannii* and *Klebsiella pneumoniae*. *ACS Infect. Dis.* **2021**, *7* (11), 2987–2998. https://doi.org/10.1021/acsinfecdis.1c00132.

(3) Umeda, T.; Tanaka, N.; Kusakabe, Y.; Nakanishi, M.; Kitade, Y.; Nakamura, K. T. Molecular Basis of Fosmidomycin's Action on the Human Malaria Parasite *Plasmodium falciparum*. *Sci Rep* **2011**, *1*, 9. https://doi.org/10.1038/srep00009.

(4) Björkelid, C.; Bergfors, T.; Unge, T.; Mowbray, S. L.; Jones, T. A. Structural Studies on *Mycobacterium tuberculosis* DXR in Complex with the Antibiotic FR-900098. *Acta Crystallogr D Biol Crystallogr* **2012**, *68* (Pt 2), 134–143. https://doi.org/10.1107/S0907444911052231.

Table S1. TABLE OF CONTENTS

Analytical data for tested compounds.	S2 – S59
**Figure S1**. Primary sequence alignment of the IspC homologs.	S59 - S61
**Table S1**. The percent identity matrix for the IspC homologs from the multiple sequence alignment performed using Clustal Omega.	S61
**Figure S2.** Half-maximal inhibitory concentrations (IC_{50}s) of *N*-acyl FOS(**1a**)/FR(**2a**) analogs determined against *A. baumannii* IspC (AbIspC).	S62 – S65
**Figure S3.** Half-maximal inhibitory concentrations (IC_{50}s) of *N*-acyl FOS(**1a**)/FR(**2a**) analogs determined against *K. pneumoniae* IspC (KpIspC).	S66 – S69
**References**	S69
